# Isolation, Structure Elucidation, and Antiproliferative Activity of Butanolides and Lignan Glycosides from the Fruit of *Hernandia nymphaeifolia*

**DOI:** 10.3390/molecules24214005

**Published:** 2019-11-05

**Authors:** Simayijiang Aimaiti, Yohei Saito, Shuichi Fukuyoshi, Masuo Goto, Katsunori Miyake, David J. Newman, Barry R. O’Keefe, Kuo-Hsiung Lee, Kyoko Nakagawa-Goto

**Affiliations:** 1 School of Pharmaceutical Sciences, College of Medical, Pharmaceutical and Health Sciences, Kanazawa University, Kanazawa 920-1192, Japan; ismayil507@stu.kanazawa-u.ac.jp (S.A.); saito-y@staff.kanazawa-u.ac.jp (Y.S.); fukuyosi@p.kanazawa-u.ac.jp (S.F.); 2 Natural Products Research Laboratories, UNC Eshelman School of Pharmacy, University of North Carolina at Chapel Hill, Chapel Hill, NC 27599-7568, USA; goto@med.unc.edu (M.G.); khlee@unc.edu (K.-H.L.); 3 Tokyo University of Pharmacy and Life Sciences, Hachioji, Tokyo 192-0392, Japan; miyake@toyaku.ac.jp; 4 NIH Special Volunteer, Wayne, PA 19087, USA; newmand@mail.nih.gov; 5 Natural Products Branch, Developmental Therapeutics Program, Division of Cancer Treatment and Diagnosis, National Cancer Institute, NCI at Frederick, Frederick, MD 21702-1201, USA; okeefeba@mail.nih.gov; 6 Molecular Targets Program, Center for Cancer Research, National Cancer Institute, NCI at Frederick, Frederick, MD 21702-1201, USA; 7 Chinese Medicine Research and Development Center, China Medical University and Hospital, Taichung 40447, Taiwan

**Keywords:** *Hernandia nymphaeifolia*, butanolides, lignan glycosides, antiproliferative activity

## Abstract

Seven new butanolides, peltanolides A–G (**1**–**7**), and two lignan glucosides, peltasides A (**8**) and B (**9**), along with eleven known compounds, **10**–**20**, were isolated from a crude CH_3_OH/CH_2_Cl_2_ (1:1) extract of the fruit of *Hernandia nymphaeifolia* (Hernandiaceae). The structures of **1**–**9** were characterized by extensive 1D and 2D NMR spectroscopic and HRMS analysis. The absolute configurations of newly isolated compounds **1**–**9** were determined from data obtained by optical rotation and electronic circular dichroism (ECD) exciton chirality methods. Butanolides and lignan glucosides have not been isolated previously from this genus. Several isolated compounds were evaluated for antiproliferative activity against human tumor cell lines. Lignans **15** and **16** were slightly active against chemosensitive tumor cell lines A549 and MCF-7, respectively. Furthermore, both compounds displayed significant activity (IC_50_ = 5 µM) against a P-glycoprotein overexpressing multidrug-resistant tumor cell line (KB-VIN) but were less active against its parent chemosensitive cell line (KB).

## 1. Introduction

**Figure 1:**
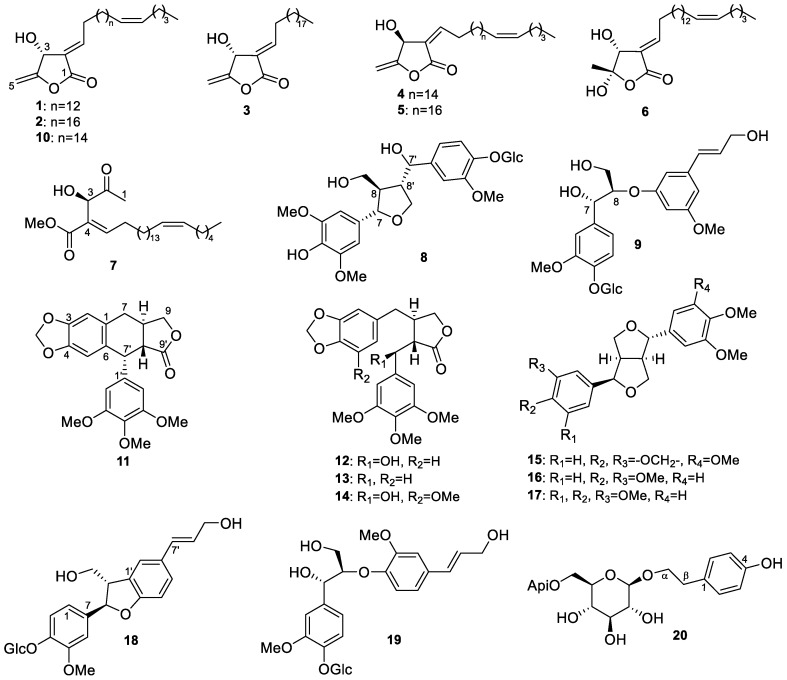
Isolated compounds (**1**−**20**) from *H. nymphaeifolia*.

Plants in the genus *Hernandia* (Hernandiaceae) are found in subtropical and tropical areas [1]. They contain diverse bioactive secondary metabolites, especially lignans, including podophyllotoxin analogues [2,3], and benzylisoquinolines [4], including aporphines [5,6,7]. These compounds exhibit various biological activities, including significant cytotoxic [8,9], antiplasmodial [9,10], and antibacterial activities [2]. *H. nymphaeifolia* (C.Presl) Kubitzki (synonym: *H. peltata* Meisn.) is a common coastal tree and grows to 12–20 m in height. This plant has been used for the treatment of abdominal pains, boils, cough, diarrhea, eye problems, and convulsions as a traditional medicine in western Samoa [11]. A CH_3_OH/CH_2_Cl_2_ (1:1) extract of *H. nymphaeifolia* (N053499, originally described as *H. peltata*) provided by the U.S. National Cancer Institute Natural Products Branch (NCI, Frederick, MD, USA) exhibited broad cytotoxicity in the NCI-60 human tumor cell line (HTCL) assay, possibly due to the above or similar cytotoxic constituents. To supplement the reported phytochemical research on *H. nymphaeifolia* [2,4,9,12,13,14], we conducted a thorough study to identify new chemical compounds as part of our continuing investigation of rainforest plants. Accordingly, the extract of N053499 yielded seven new butanolides, peltanolides A–G (**1**–**7**), and two new lignan glycosides, peltasides A (**8**) and B (**9**), as well as eleven known compounds **10**–**20** (Figure 1). Herein, we report the details of isolation, structure elucidation, and cytotoxicity of isolated compounds from *H. nymphaeifolia*.

## 2. Results and Discussion

### 2.1. Structure Elucidation of Isolated Compounds from H. nymphaeifolia

The CH_3_OH/CH_2_Cl_2_ (1:1) extract of *H. nymphaeifolia* (fruit, N053499) was firstly partitioned with water and *n*-hexane. The water fraction was further partitioned with EtOAc and *n*-BuOH. All fractions were subjected to a combination of column chromatography, preparative HPLC, and preparative TLC using silica gel and octadecylsilyl (ODS) to give seven new butanolides, peltanolides A–G (**1**–**7**), and two new lignan glycosides, peltasides A (**8**) and B (**9**), as well as eleven known compounds, tambouranolide (**10**) [15], deoxypodophyllotoxin (**11**) [16], podorhizol (**12**) [17], bursehernin (**13**) [18], (2*S*,3*S*)-(+)-5′-methoxyyatein (**14**) [19], epiashantin (**15**) [20], epieudesmin (**16**) [21], (1*S*,3a*R*,4*R*,6a*R*)-1-(3,4-dimethoxyphenyl)-4-(3′,4′,5′-trimethoxyphenyl)tetrahydro-1*H*,3*H*-furo-[3–*c*]furan (**17**) [20], (7*R*,8*S*)-dehydrodiconiferyl alcohol-4-*O*-β-d-glucoside (**18**) [22], alaschanioside A (**19**) [23], and osmanthuside H (**20**) [24]. The structures of all known compounds were identified by comparison of their spectroscopic data with reported values.

**Figure 2:**
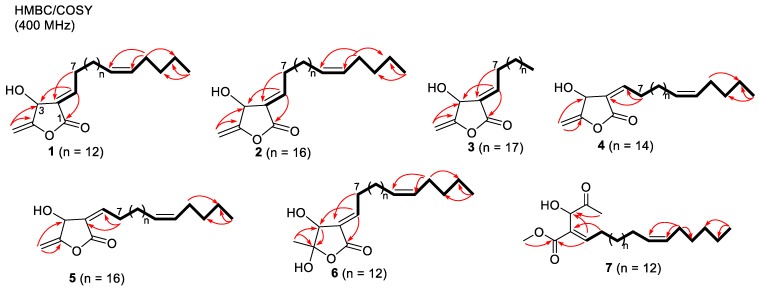
Selected HMBC correlations (arrows in red), COSY connectivities (bold lines) for compounds **1−7**.

**Figure 3:**
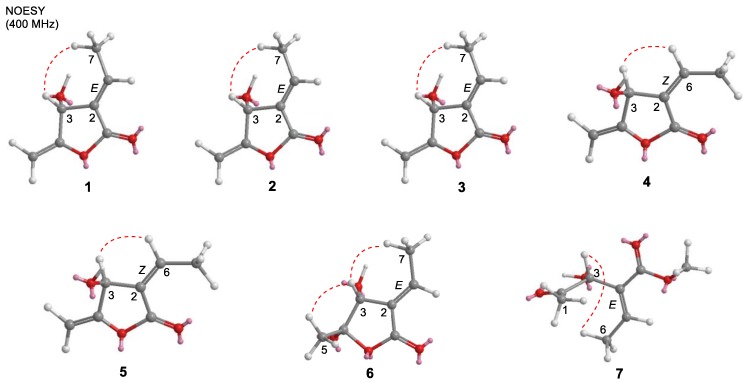
Key NOESY (red dashed lines) correlations for compounds **1**–**7**.

Compound **1** was obtained as a yellow amorphous solid: [α]^25^_D_ + 27.3 (*c* 0.075, CHCl_3_). It gave a [M]^+^ peak at *m/z* 390.3137, appropriate for a molecular formula of C_25_H_42_O_3_. The ^1^H and ^13^C NMR spectra (Table 1 and Table 2) contained signals attributed to oxymethine [δ_H_ 5.26 (1H, brs); δ_C_ 66.5, C-3], methylidene [δ_H_ 4.96 (1H, dd, *J* = 2.8, 1.4 Hz), 4.72 (1H, dd, *J* = 2.8, 1.4 Hz); δ_C_ 91.4, 157.6, C-4,5], vinyl [δ_H_ 7.09 (1H, td, *J* = 7.8, 2.2 Hz); δ_C_ 127.3, 150.3, C-2,6], and carbonyl (δ_C_ 166.5, C-1) groups, consistent with a β-hydroxy-γ-methylene-α,β-unsaturated-γ-lactone. The chemical shifts of H-6 (δ_H_ 7.09) and H-7 (δ_H_ 2.48) as well as allylic carbon C-3 (δ_C_ 66.5) and olefinic carbon C-6 (δ_C_ 150.3) were identical with those of tambouranolide (**10**) [15] and related linderanolides and isolinderanolides [25,26] with an *E*-configured double bond [Δ^2(6)^]. This assignment was also supported by a cross-peak between H-3 and H-7 in the NOESY spectrum (Figure 3). The presence of a long aliphatic chain containing a double bond was suggested by NMR resonances for olefinic and multiple methylene carbons. The allylic (δ_C_ 27.0, 27.2) and olefinic (δ_C_ 129.8, 129.9) carbon signals in the ^13^C NMR spectrum of **1** suggested that the internal olefin has the typical *Z*-configuration, comparable with those of **10** as well as the abovementioned linderanolides and isolinderanolides with *Z*-double bonds in the side chain. In a related *E*-isomer, the allylic and olefinic carbons appeared at 32.6 and 25.6 ppm and at 131.9 and 129.3 ppm, respectively [25]. The location of the olefinic bond at Δ^20^ was based on HMBC and COSY correlations (Figure 2). From the NMR and HREIMS data, compounds **10** and **1** differ only in the number of methylene groups (16 in **10**, 14 in **1**) in the long aliphatic chain. The absolute configuration of **1** was determined from its optical rotation, which was the same as that of **10**. Furthermore, the total synthesis of peumusolide A analogues clearly proved that the optical rotation is positive for 3*R* compounds and negative for 3*S* [27,28]. Therefore, compound **1** (peltanolide A) was assigned as (2*E*,3*R*)-3-hydroxy-4-methylidene-2-[(15*Z*)-15-icosenylidene]butanolide.

Compound **2** was isolated as a yellow solid, [α]^25^_D_ +26.1 (*c* 0.12, CHCl_3_). The HREIMS data supported a molecular formula of C_29_H_51_O_3_ from the peak at *m/z* 446.3743 [M]^+^. The MS data and NMR spectra indicated four additional methylene units compared with **1**, and the optical rotation suggested the same configuration as that of **1**. Thus, compound **2** (peltanolide B) was defined as (2*E*,3*R*)-3-hydroxy-4-methylidene-2-[(19*Z*)-19-tetracosenylidene]butanolide.

Compound **3** was obtained as a colorless oil: [α]^25^_D_ +26.0 (*c* 0.07, CHCl_3_). The HREIMS data indicated a molecular formula of C_25_H_44_O_3_ from the peak at *m/z* 392.3302 [M]^+^, which was identical to that of miaolinolide [29]. One dimensional NMR spectra of **3** also displayed the similar signal pattern with one exception: the chemical shift of H-6 is δ_H_ 7.10 (1H, td, *J* = 7.8, 2.2 Hz) in **3** and δ_H_ 6.70 (1H, td, *J* = 8.0, 2.0 Hz) in miaolinolide. Thus, the Δ^2(6)^ double bond has an *E* configuration in **3**, rather than the *Z* configuration in miaolinolide [29]. This assignment was also proved that the chemical shift of H-6 in **3** was close to that of related butanolides with an *E* configuration of the Δ^2(6)^ double bond [15,25,26,30], including compounds **1** and **2**. A NOESY correlation between H-3 and H-7 (Figure 3) supported this conclusion. Based on their optical rotations, compound **3** and miaolinolide have the same absolute configuration. Hence, the structure of **3** (peltanolide C) was established as (2*E*,3*S*)-3-hydroxy-4-methylidene-2-icosylidenebutanolide.

HRFABMS of compound **4** showed a molecular formula C_27_H_46_O_3_ with a molecular ion at *m/z* 441.3357 [M + Na]^+^. The ^1^H and ^13^C NMR spectra of **4** (Table 1 and Table 2) were comparable to those of **10** but suggested different double bond [Δ^2(6)^] configurations and C-3 stereochemistries. For **4**, the Δ^2(6)^ configuration was determined as *Z* from a NOESY correlation between H-3 and H-6 (Figure 3) and the chemical shift of H-6 at 6.69 ppm rather than ca. 7.10 ppm for the *E* form. The C-3 stereochemistry was determined as *S* by comparison of optical rotations, [α]^25^_D_ −29.7 (*c* 0.02, CHCl_3_) for **4** and [α]^25^_D_ + 18.0 (*c* 0.03, CHCl_3_) for **10** with 3*R*. Thus, compound **4** (peltanolide D) was determined as (2*Z*,3*S*)-3-hydroxy-4-methylidene-2-[(17*Z*)-17-docosenylidene]butanolide.

Compound **5** was obtained as a yellow solid and displayed a peak at *m/z* 446.3749 [M]^+^ in the HREIMS spectrum, which agreed with a molecular formula of C_29_H_50_O_3_ and two additional methylene units (C_2_H_4_) compared with **4**. This finding was also supported by the two NMR spectra. Both compounds also have the same absolute configurations based on their optical rotations, [α]^25^_D_ −21.1 (*c* 0.015, CHCl_3_) for **5** and [α]^25^_D_ −29.7 (*c* 0.02, CHCl_3_) for **4**. Thus, compound **5** (peltanolide E) was defined as (2*Z*,3*S*)-3-hydroxy-4-methylidene-2-[(15*Z*)-15-icosenylidene]butanolid.

**Figure 4:**
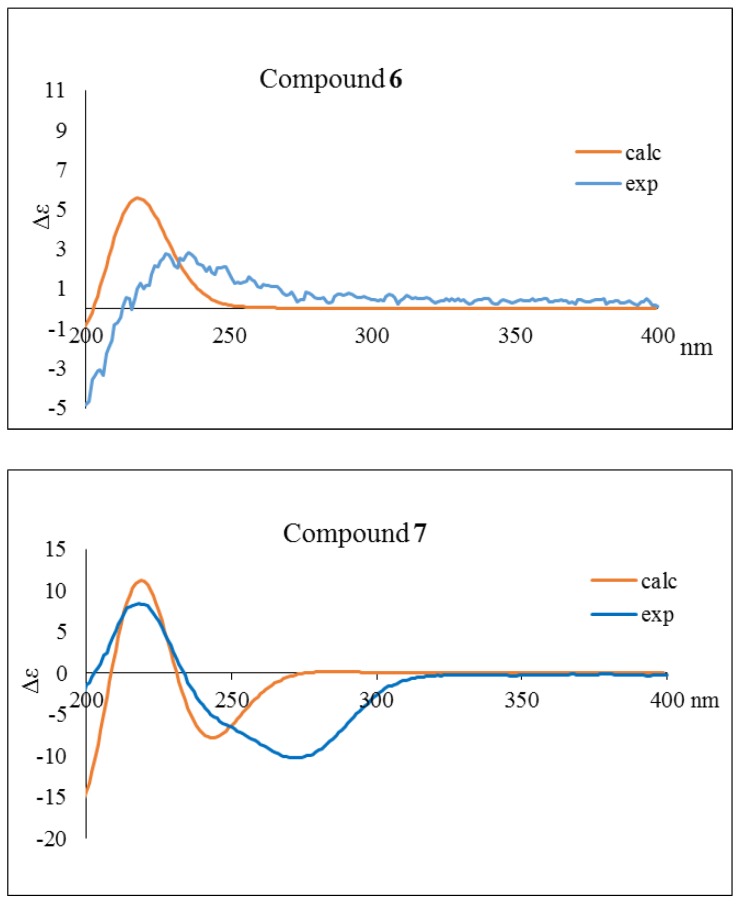
Experimental and calculated ECD spectra of compounds **6** and **7**.

Compound **6** was isolated as a colorless oil. The HREIMS data indicated a molecular formula of C_25_H_44_O_4_ from the peak at *m/z* 408.3230 [M]^+^. Compared with **1**, the ^1^H and ^13^C NMR spectra of **6** (Table 1 and Table 2) showed the absence of signals for a methylidene group and the presence of signals for a methyl group [δ_H_ 1.62 (3H, s)/δ_C_ 26.8] and a doubly oxygenated carbon (δ_C_ 100.1). The doubly oxygenated carbon was assigned as C-4 with an attached methyl group; these assignments were confirmed by HMBC correlations (Figure 2). A NOESY correlation between H-7 and H-3 as well as the chemical shift of H-6 at 7.04 ppm were consistent with Δ^2(6)^ being the *E*-isomer. The stereochemistry of C-3 was determined as *R* based on the optical rotation [α]^25^_D_ +116.0 (*c* 0.015, CHCl_3_) by comparison with related 4-hydroxybutanolides [31,32,33]. The NOESY correlation between H-3 and H-5 supported the 4*S* stereochemistry (Figure 3). TDDFT-ECD calculation was also sorted the (3*R*,4*S*) absolute configuration (Figure 4). Therefore, compound **6** (peltanolide F) was assigned as (2*E*,3*R*,4*S*)-3,4-dihydroxy-5-methyl-2-[(15*Z*)-15-icosenylidene]butanolide.

**Figure 5:**
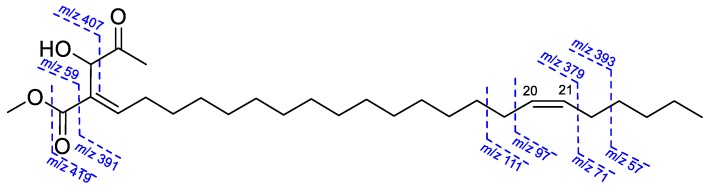
EIMS fragmentation of **7**.

Compound **7** has the molecular formula, C_28_H_50_O_4_, based on the peak at *m/z* 450.3702 [M]^+^ in the HREIMS. All NMR data and EIMS fragment peaks (Figure 5) of **7** were identical to those of illigerone A [34]. However, the ECD spectrum of **7** exhibited a different Cotton effect from that of illigerone A, and TDDFT-ECD calculation was indicated the 3*R* absolute configuration (Figure 4). In addition, the experimental optical rotation, [α]^25^_D_ −78.5 (*c* 0.03, acetonitrile), of **7** had a negative (levorotary) rather than positive (dextrorotary) value, as found with illigerone A [34]. We concluded that compound **7** (peltanolide G) is (3*R*,4*E*,20*Z*)-3-hydroxy-4-(2-methoxy-2-oxo)hexacosa-4,20-dien-2-one, the enantiomer of illigerone A.

**Figure 6:**
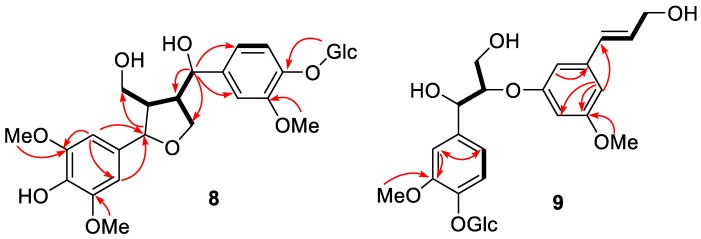
Selected HMBC correlations (arrows in red), COSY connectivities (bold lines) for **8** and **9**.

**Figure 7:**
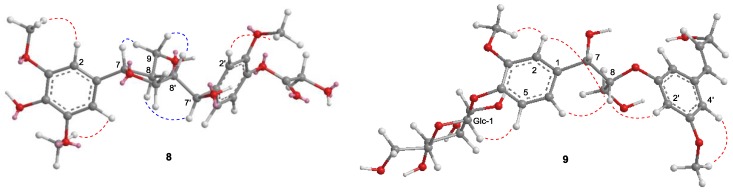
Key NOESY correlations (red lines) and Key ROESY correlations (blue lines) for **8** and **9**.

**Figure 8:**
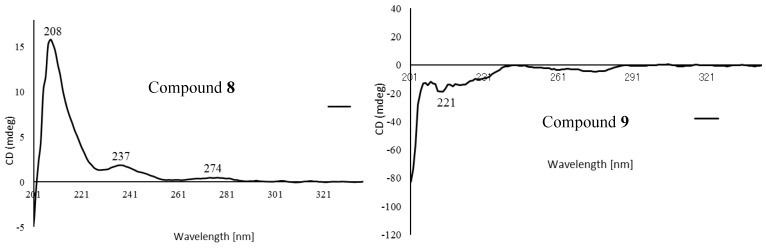
Experimental ECD spectra of compounds **8** and **9**.

Compound **8** was obtained as a yellow solid, and its molecular formula was determined to be C_27_H_36_O_13_ on the HRFABMS ion at *m/z* 591.2022 [M + Na]^+^. The ^1^H NMR data displayed five aromatic [δ_H_ 7.11 (1H, d, *J* = 8.2 Hz), 6.92 (1H, d, *J* = 1.8 Hz), 6.82 (1H, dd, *J* = 8.2, 1.8 Hz), and 6.52 (2H, s, overlap)], two oxymethine [δ_H_ 4.63 (1H, d, *J* = 7.3 Hz), 4.52 (1H, d, *J* = 8.2 Hz)], four oxymethylene protons [δ_H_ 4.26 (1H, dd (*J* = 9.0, 4.6 Hz), 3.92 (1H, m), 3.87 (1H, m), and 3.63 (1H, m)], three methoxy groups [δ_H_ 3.84 (6H, s), and 3.83 (3H, s)], and two methine protons [δ_H_ 2.53 (1H, m), 1.89 (1H, m)]. In addition, a glucopyranosyl anomeric proton was observed at δ_H_ 4.84 (1H, m). The ^13^C NMR spectrum showed 27 carbon signals, six from a glucose unit and three methoxy groups and the remaining 18 carbons from the lignan skeleton. The spectroscopic data of **8** resembled those of the known compound, (7*S*,8*R*,7′*S*,8′*S*)-4,9,7′-trihydroxy-3,3′-dimethoxy-7,9′-epoxylignan-4′-*O*-β-d-glucopyrano-side [35], except for the absence of the H-3 aromatic proton in the ^1^H NMR spectrum and the presence of an additional methoxy group in **8**. The HMBC (Figure 6) and NOESY (Figure 7) spectra agreed with this structure, and the observed ROESY correlations between H-7/H-9, H-8/H-7′, and H-8′/H-9 (Figure 7) strongly suggested trans configurations of H-7/H-8 and H-8/H-8′. The CD spectrum of **8** showed positive Cotton effects at 237 nm and 274 nm (Figure 8), which were identical with those of the known compound [35]. Thus, the structure of **8** (peltaside A) was determined as (7*S*,8*R*,7′*S*,8′*S*)-4,9,7′-trihydroxy-3,5,3′-trimethoxy-7,9′-epoxylignan-4′-*O*-β-d-glucopyranoside.

Compound **9** was obtained as a yellow solid and its HRFABMS (*m/z* 561.1958 [M + Na]^+^) indicated the molecular formula C_26_H_34_O_12_. The 1H NMR spectrum of **9** displayed the signals for a trans-olefinic, two oxygenated methine, two oxygenated methylene, and six aromatic protons, as well as a β-glucose and two methoxy groups (Table 3). In addition, its ^13^C-NMR spectrum showed the signals for 26 carbons, including 12 aromatic, two methoxy, two olefinic, and six glucopyranosyl carbons (Table 3). The COSY, HMQC, HMBC, and NOESY spectra suggested that the glucopyranosyl moiety was attached to C-4 (Figure 5 and Figure 6). The relative configuration between C-7 and C-8 was assigned as erythro based on the small coupling constant (*J* = 4.8 Hz) in ^1^H NMR (Figure S57). The absolute configuration of **9**, which showed a negative Cotton effect at 221 nm in the CD spectrum (Figure 8), was determined to be 7*S*,8*R* via a comparison with that of reported analogues [36,37,38]. Hence, compound **9** (peltaside B) is (7*S*,8*R*,7′*E*)-7,9,9′-trihydroxy-3,5′-dimethoxy-8-3′-oxyneolign-7′-ene-4-*O*-β-d-glucopyranoside.

### 2.2. Antiproliferative Activity of Isolated Compounds from H. nymphaeifolia

Compounds **1**, **2**, **8**, **10**, and **13**–**18** were evaluated for antiproliferative effects against five human tumor cell lines, A549 (lung carcinoma), MCF-7 (estrogen receptor-positive and HER2-negative breast cancer), MDA-MB-231 (triple negative breast cancer), KB (cervical cancer cell line HeLa derivative), and P-glycoprotein (P-gp)-overexpressing multidrug-resistant (MDR) KB subline, KB-VIN (Table 4). The remaining compounds were not tested due to insufficient quantities. Butanolide **10** slightly inhibited MCF-7 and KB-VIN tumor cell growth with an IC_50_ value of **9** μM. Both lignans **15** and **16** showed antiproliferative activity against chemosensitive A549 and MCF-7 tumor cell lines, while **16** was also active against MDA-MB-231. Interestingly, compounds **15** and **16** also displayed moderate activity against the MDR cell line (KB-VIN) with an IC_50_ value of 5 μM but were less active against its parent chemosensitive cell line (KB). Compounds **1**, **2**, **8**, **14** and **18** exhibited no activity against all tested cell lines. These results demonstrated that the CH_3_OH/CH_2_Cl_2_ (1:1) extract of *H. nymphaeifolia* contained antiproliferative natural products, which showed broad spectrum against HTCLs including MDR cells and could also work synergistically against MDR cells.

## 3. Materials and Methods

### 3.1. General Experimental Procedures

Infrared spectra (IR) were obtained with a Thermo Fisher Scientific (Waltham, MA, USA) NICOLET iS5 FT-TR spectrometer from samples in CHCl_3_ and MeOH. NMR spectra were measured on JEOL (Akishima, Tokyo, Japan) JNM-ECA600 and JNM-ECS400 spectrometers with tetramethylsilane as an internal standard, and chemical shifts are stated as δ values. HRMS data were recorded on a JEOL JMS-700 Mstation (FAB or EI) mass spectrometer. Analytical and preparative TLC were carried out on precoated silica gel 60F254 and RP-18F254 plates (0.25 or 0.50 mm thickness; Merck, Darmstadt, Germany). MPLC was performed on a Combiflash *R*_f_ (Teledyne Isco, Lincoln, NE, USA) with silica gel and C18 cartridges (Biotage, Uppsala Sweden). Preparative HPLC was carried out with a GL Science (Shinjuku, Tokyo, Japan) recycling system (PU714 pump and UV702 UV-Vis detector) using an InertSustain C18 column (5 µM, 20 × 250 mm).

### 3.2. Plant Material

The crude CH_3_OH/CH_2_Cl_2_ (1:1) extract (#N053499) from fruit of *H. nymphaeifolia* (Presl) Kubitzki (originally identified as *H. peltata*), collected in Java (Indonesia) was provided by NCI/NIH. The plant was collected on May 25, 1992 in a sandy habitat in the Ujung Kulon Reserve by A. McDonald. A voucher specimen for the plant collection was deposited at the Smithsonian Institution (Washington, WA, USA) and voucher extracts were deposited at the NCI (Frederick, MD, USA) and Kanazawa University (Kanazawa, Ishikawa, Japan).

### 3.3. Extraction and Isolation

The crude extract N053499 (25.0 g) was dissolved in CH_3_OH/H_2_O (9:1) then partitioned with n-hexane, EtOAc, and *n*-BuOH, yielding *n*-hexane (17.4 g), EtOAc (3.94 g), *n*-BuOH (1.72 g), and H_2_O (0.997 g) fractions. The EtOAc-soluble fraction was subjected to silica gel column chromatography (CC) with a gradient system [*n*-hexane/EtOAc 100:0 (500 mL)→90:10 (500 mL)→70:30 (1000 mL)→50:50 (1000 mL)→30:70 (1000 mL)→10:90 (1000 mL)→0:100 (500 mL)→EtOAc/MeOH 50:50 (500 mL)→MeOH (1000 mL)] to yield nine fractions, F1–F9. F3 (123 mg) was subjected to silica gel MPLC (RediSep Rf GOLD High Performance 4 g) eluted with *n*-hexane/EtOAc (9:1 to 0:1) to afford five subfractions 3a–e. Subfraction 3b (21.6 mg) was purified by repeated recycling reversed-phase preparative HPLC with H_2_O/MeOH (1:19) to provide compounds **2** (2.2 mg), **3** (1.4 mg), and **10** (1.2 mg). F4 (77.9 mg) was subjected to silica gel CC eluted with CH_2_Cl_2_ followed by MeOH to yield eight subfractions 4a–h. Subfraction 4d (1.0 mg) was further separated by preparative normal-phase TLC with CH_2_Cl_2_ to afford compound **5** (0.4 mg). Subfraction 4f (4.6 mg) was purified by repeated recycling preparative HPLC with H_2_O/MeOH (1:2) to afford compound **7** (0.6 mg). Subfraction 4h (54.0 mg) was purified by preparative normal-phase TLC with CH_2_Cl_2_/EtOAc (19:1) to afford compounds 13 (13.2 mg) and 15 (2.1 mg). F6 was subjected to silica gel CC eluted with CH_2_Cl_2_/EtOAc (19:1 to 0:1) followed by MeOH to obtain six subfractions, 6a–f. Subfraction 6b (37.5 mg) was purified by repeated recycling preparative HPLC with H_2_O/MeOH (1:2) to afford compounds **12** (7.1 mg) and **16** (20.3 mg). Subfraction 6c (15.9 mg) was purified by repeated recycling preparative HPLC with H_2_O/MeOH (1:2), to provide compound **17** (4.1 mg). The *n*-hexane fraction (12.0 g) was subjected to silica gel MPLC (RediSep *R*_f_ GOLD High Performance 120 g) with a gradient system [*n*-hexane/CH_2_Cl_2_ 1:1 (600 mL)→2:3 (1400 mL)→3:7 (1200 mL)→4:1 (1400 mL)→CH_2_Cl_2_ (1200 mL)→CH_2_Cl_2_/EtOAc 1:1 (1000 mL)→EtOAc (1000 mL)→MeOH (1400 mL)] to yield 15 fractions, F1–F15. F6 (695 mg) was applied to silica gel MPLC (RediSep Rf GOLD High Performance 24 g) eluted with *n*-hexane/EtOAc (9:1 to 0:1) followed by MeOH to yield ten subfractions 6a–j. Subfraction 6e (197 mg) was subjected to silica gel CC eluted with *n*-hexane/EtOAc (2:3 to 0:1) followed by MeOH to yield 11 subfractions 6e1–11. Subfraction 6e5 (4.9 mg) was purified by preparative normal-phase TLC with *n*-hexane/CH_2_Cl_2_ (3:1) to afford compound 6 (0.4 mg). F11 (1.12 g) was applied to silica gel MPLC (RediSep Rf GOLD High Performance 24 g) with *n*-hexane/EtOAc (9:1 to 0:1) followed by MeOH to yield seven subfractions 11a–g. Subfraction 11f (535 mg) was purified by MPLC on ODS-25 (YMC-DispoPack AT 12 g) with H_2_O/CH_3_OH (1:3), followed by recycling preparative HPLC with H_2_O/MeOH (1:2) to afford compounds **13** (0.4 mg) and **14** (0.2 mg). F13 (1.35 g) was subjected to silica gel MPLC (RediSep Rf GOLD High Performance 24 g) with *n*-hexane/CH_2_Cl_2_/EtOAc (1:1:0 to 0:0:1) followed by MeOH to yield ten subfractions 13a–j. Subfraction 13e (48.2 mg) was purified by ODS preparative TLC eluted three times using MeOH to afford compounds **1** (3.2 mg), **2** (1.1 mg), and **10** (1.0 mg). The *n*-BuOH-soluble fraction (1.72 g) was subjected to silica gel MPLC (RediSep *R*_f_ GOLD High Performance 120 g) with a gradient system [CHCl_3_/MeOH 1:0 (1000 mL)→10:1 (1400 mL)→5:1 (1200 mL)→1:1 (1800 mL)→MeOH (1400 mL)] to yield nine fractions, F1–F9. F1 (147 mg) was subjected to silica gel CC eluted with CH_2_Cl_2_/EtOAc (1:0 to 0:1) followed by MeOH to obtain 14 subfractions, 1a–n. Compound **4** (0.3 mg) was obtained from subfraction 1e. Subfraction 1g (3.3 mg) was purified by preparative normal-phase TLC with CH_2_Cl_2_/EtOAc (95:5) to afford compound **10** (1.3 mg). Subfraction 1k was purified by recycling preparative HPLC with H_2_O/MeOH (1:3) to afford compounds **11** (1.8 mg), **14** (0.3 mg), and **17** (0.4 mg). F2 (44.3 mg) was subjected to silica gel CC eluted with CH_2_Cl_2_/EtOAc (9:0 to 0:1) followed by MeOH to obtain nine subfractions, 2a–i. Subfraction 2b (1.1 mg) was purified by ODS preparative TLC eluted three times using H_2_O/MeOH (1:8) to yield compound **13** (0.6 mg). F3 (33.8 mg) was purified by preparative normal-phase TLC with CHCl_3_/MeOH (9:1) to afford compound **8** (1.0 mg). F5 (149 mg) was subjected to silica gel CC eluted with CH_2_Cl_2_/MeOH (10:1 to 1:1) followed by MeOH to obtain seven subfractions, 5a–g. Subfraction 5d (75.6 mg) was purified by MPLC on ODS-25 (YMC-DispoPack AT 12 g) with H_2_O/MeOH (1:3), followed by recycling preparative HPLC with H_2_O/MeOH (2:3) to afford compounds **9** (1.3 mg), **18** (2.3 mg), **19** (1.0 mg), and **20** (1.2 mg).

#### 3.3.1. Peltanolide A (**1**)

Yellow amorphous solid; [α]^25^_D_ +27.3 (*c* 0.075, CHCl_3_); IR ν_max_ (CHCl_3_) cm^−1^ 2923, 2853, 2017, 1733, 1457, 1278, 1219; ^1^H and ^13^C NMR, Table 1 and Table 2; HREIMS *m/z* 390.3137 [M]^+^ (calcd for C_25_H_42_O_3_, 390.3134).

#### 3.3.2. Peltanolide B (**2**)

Yellow amorphous solid; [α]^25^_D_ +26.1 (*c* 0.12, CHCl_3_); IR ν_max_ (CHCl_3_) cm^−1^ 2923, 2852, 1731, 1464, 1265, 1074; ^1^H and ^13^C NMR, Table 1 and Table 2; HREIMS *m/z* 446.3743 [M]^+^ (calcd for C_29_H_51_O_3_, 446.3760).

#### 3.3.3. Peltanolide C (**3**)

Colorless oil; [α]^25^_D_ +26.0 (*c* 0.07, CHCl_3_); IR ν_max_ (CHCl_3_) cm^−1^ 2916, 2849, 2016, 1750, 1678, 1470, 1278, 1184; ^1^H and ^13^C NMR, Table 1 and Table 2; HREIMS *m/z* 392.3302 [M]^+^ (calcd for C_25_H_44_O_3_, 392.3290).

#### 3.3.4. Peltanolide D (**4**)

Yellow amorphous solid; [α]^25^_D_ −29.7 (*c* 0.02, CHCl_3_); IR ν_max_ (CHCl_3_) cm^−1^ 2923, 2852, 1783, 1733, 1465, 1373, 1287; ^1^H and ^13^C NMR, Table 1 and Table 2; HRFABMS *m/z* 441.3357 [M + Na]^+^ (calcd for C_27_H_46_O_3_Na, 441.3345).

#### 3.3.5. Peltanolide E (**5**)

Yellow amorphous solid; [α]^25^_D_ −21.1 (*c* 0.015, CHCl_3_); IR ν_max_ (CHCl_3_) cm^−1^ 2922, 2852, 2017, 1770, 1731, 1557, 1458, 1375, 1287; ^1^H and ^13^C NMR, Table 1 and Table 2; HREIMS *m/z* 446.3749 [M]^+^ (calcd for C_29_H_50_O_3_, 446.3760).

#### 3.3.6. Peltanolide F (**6**)

Colorless oil; [α]^25^_D_ +116.0 (*c* 0.015, CHCl_3_); IR ν_max_ (CHCl_3_) cm^−1^ 2923, 2852, 1733, 1558, 1540, 1456, 1287; ^1^H and ^13^C NMR, Table 1 and Table 2; HREIMS *m/z* 408.3230 [M]^+^ (calcd for C_25_H_44_O_4_, 408.3240).

#### 3.3.7. Peltanolide G (**7**)

Colorless oil; [α]^25^_D_ −78.5 (*c* 0.03, acetonitrile); IR ν_max_ (CHCl_3_) cm^-1^ 2922, 2852, 2016, 1717, 1669, 1558, 1456, 1436; ^1^H and ^13^C NMR, Table 1 and Table 2; HREIMS *m/z* 450.3702 [M]^+^ (calcd for C_28_H_50_O_4_, 450.3709).

#### 3.3.8. Peltaside A (**8**)

Yellow solid; [α]^25^_D_ +5.6 (*c* 0.055, MeOH); IR ν_max_ (CHCl_3_) cm^−1^ 3330, 2945, 2833, 1645, 1514, 1450, 1112; ^1^H and ^13^C NMR, Table 3; HRFABMS *m/z* 591.2022 [M + Na]^+^ (calcd for C_27_H_36_O_13_Na, 591.2054).

#### 3.3.9. Peltaside B (**9**)

Yellow solid; [α]^25^_D_ −71.8 (*c* 0.065, MeOH); IR ν_max_ (CHCl_3_) cm^−1^ 3386, 3293, 1657, 1511, 1265; ^1^H and ^13^C NMR, Table 3; HRFABMS *m/z* 561.1958 [M + Na]^+^ (calcd for C_26_H_34_O_12_Na, 561.1948).

### 3.4. Calculation of ECD Spectra

Preliminary conformational analysis for each compound was carried out by using CONFLEX8 with the MMFF94 force field. The conformers were further optimized in MeCN by density functional theory (DFT) method with the B3LYP functional and 6–31(d) basis set. The ECD spectrum was calculated by the time-dependent DFT (TDDFT) method with the CAM-B3LYP functional and TZVP basis set. The calculation was completed by the use of conformers within 2 kcal/mol predicted in MeCN. The solvent effect was introduced by the conductor-like polarizable continuum model (CPCM). The DFT optimization and TDDFT-ECD calculation were performed using Gaussian09 (Gaussian, Inc., Wallingford, CT, USA). The calculated spectrum was displayed by GaussView 5.0.920 with the peak half-width at half height being 0.333 eV. The Boltzmann-averaged spectrum at 298.15K was calculated using Excel 2016 (Microsoft Co., Redmond, WA, USA). The calculations were re-optimized according to the literature [39].

### 3.5. Assay for Antiproliferative Activity

Antiproliferative activity of the compounds was determined by the sulforhodamine B (SRB) assay as described previously [40]. Briefly, cell suspensions were seeded on 96-well microtiter plates at a density of 4000–12,000 cells per well and cultured for 72 h with test compound. The cells were fixed in 10% trichloroacetic acid and then stained with 0.04% SRB. The absorbance at 515 nm of 10 mM Tris base-solubilized protein-bound dye was measured using a microplate reader (ELx800, BioTek, Winooski, VT, U.S) operated by Gen5 software (BioTek). IC_50_ data were calculated statistically (MS Excel) from at least three independent experiments performed with duplication (*n* = 6). All human tumor cell lines, except KB-VIN, were obtained from the Lineberger Comprehensive Cancer Center (UNC-CH, Chapel Hill, NC, USA) or from ATCC (Manassas, VA, USA). KB-VIN was a generous gift from Professor Y.-C. Cheng of Yale University (New Haven, CT, USA).

## 4. Conclusions

As part of our continuing investigation of rainforest plants, we conducted a thorough study to identify new chemical compounds to supplement the reported phytochemical research on *H. nymphaeifolia*. Consequently, a CH_3_OH/CH_2_Cl_2_ (1:1) extract of *H. nymphaeifolia* (N053499) provided by NCI yielded seven new butanolides, peltanolides A–G (**1**–**7**), and two new lignan glycosides, peltasides A (**8**) and B (**9**), as well as eleven known compounds **10**–**20**. This is the first report to identify butanolides and lignan glucosides from this genus. The evaluation of antiproliferative activity against human tumor cell lines revealed that lignans **15** and **16** were slightly active against chemosensitive tumor cell lines A549 and MCF-7, respectively. Interestingly, both compounds displayed significant activity with IC_50_ valued of 5 µM against a P-glycoprotein overexpressing MDR tumor cell line (KB-VIN) although they were less active against its parent chemosensitive cell line (KB).

## Figures and Tables

**Table 1:** ^1^H NMR Spectroscopic data of compounds **1**−**7**.

	1*^a^* (CDCl_3_)	2*^b^* (CDCl_3_)	3*^b^* (CDCl_3_)	4*^b^* (CDCl_3_)	5*^b^* (CDCl_3_)	6*^a^* (CDCl_3_)	7*^b^* (CDCl_3_)
Position	*δ*_H_ (*J* in Hz)	*δ*_H_ (*J* in Hz)	*δ*_H_ (*J* in Hz)	*δ*_H_ (*J* in Hz)	*δ*_H_ (*J* in Hz)	*δ*_H_ (*J* in Hz)	*δ*_H_ (*J* in Hz)
1							2.15 s
3	5.26 brs	5.26 brs	5.26 brd (5.6)	5.11 m	5.10 brd (7.3)	4.82 brs	4.89 d (4.2)
5a	4.72 dd (2.8, 1.4)	4.72 dd (2.8, 1.4)	4.72 dd (2.8, 1.4)	4.67 dd (2.8, 1.4)	4.66 dd (2.8, 1.4)	1.62 s	7.07 t (8.0)
5b	4.96 dd (2.8, 1.4)	4.96 dd (2.8, 1.4)	4.96 dd (2.8, 1.4)	4.89 dd (2.8, 1.4)	4.89 dd (2.8, 1.4)		
6	7.09 td (7.8, 2.2)	7.09 td (7.8, 2.2)	7.10 td (7.8, 2.2)	6.69 td (7.8, 2.2)	6.67 td (7.8, 2.2)	7.04 td (7.8, 2.2)	2.34 td (14.8, 8.0)
7	2.48 m	2.48 m	2.48 m, 2.43 m	2.78 m	2.76 m	2.38 m	1.50 m
8	1.52 m	1.52 m	1.53 m	1.46 m	1.45 m	1.52 m	1.25 m^p^
9–18	1.26 m^c^	1.26 m^e^	1.25 m^g^	1.25 m^h^	1.25 m^j^	1.25 m^m^	1.25 m^p^
19	2.00 m	1.26 m^e^	1.25 m^g^	1.25 m^h^	1.25 m^j^	2.01 m^n^	2.00 m^q^
20	5.36 m^d^	1.26 m^e^	1.25 m^g^	1.25 m^h^	1.25 m^j^	5.34 t (4.8)^o^	5.34 t (4.8)^r^
21	5.36 m^d^	1.26 m^e^	1.25 m^g^	2.02 m	1.25 m^j^	5.34 t (4.8)^o^	5.34 t (4.8)^r^
22	2.02 m	1.26 m^e^	1.25 m^g^	5.35 m^i^	1.25 m^j^	2.01 m^n^	2.00 m^q^
23	1.26 m^c^	2.00 m	1.25 m^g^	5.35 m^i^	2.01 m^k^	1.25 m^m^	1.25 m^p^
24	1.32 m	5.36 m^f^	1.25 m^g^	2.02	5.33 m^l^	1.31	1.25 m^p^
25	0.89 t (6.9)	5.36 m^f^	0.89 br t (7.3)	1.25 m^h^	5.33 m^l^	0.89 t (6.0)	1.31 m
26		2.02 m		1.33 m	2.01 m^k^		0.89 t (6.0)
27		1.26 m^e^		0.88 t (6.9)	1.25 m^j^		
28		1.32 m			1.32 m		
29		0.89 t (7.3)			0.87 t (6.9)		
2-OH		2.26 m					
3-OH							4.00 brs
OCH_3_							3.72 s

*^a^* 600 MHz, *^b^* 400 MHz, *^c–r^* Overlapping signals.

**Table 2:** ^13^C NMR Spectroscopic data in CDCl_3_ of compounds **1−7**.

	1*^a^*	2*^b^*	3*^b^*	4*^b^*	5*^b^*	6*^a^*	7*^b^*
Position	*δ*_c_	*δ*_c_	*δ*_c_	*δ*_c_	*δ*_c_	*δ*_c_	*δ*_c_
1	166.5	166.5	166.3	163.1		166.4	24.8
2	127.3	127.3	127.2	127.4	127.3	125.2	206.3
3	66.5	66.5	66.6	68.9	68.9	70.9	73.4
4	157.6	157.6	157.5	160.1	160.2	100.1	129.8
5	91.4	91.4	91.5	90.3	90.4	26.8	149.0
6	150.3	150.3	150.3	151.4	151.4	151.9	28.7
7	29.8	29.8	29.8	29.8	29.8	30.1	29.3
8	28.4	28.4	28.4–29.6^d^	28.4	28.4	29.5–29.9^g^	29.4–30.0^h^
9–18	29.4–30.0	29.4–30.0^c^	28.4–29.6^d^	29.4–30.0^e^	29.4–30.0^f^	29.5–29.9^g^	29.4–30.0^h^
19	27.0	29.4–30.0^c^	28.4–29.6^d^	29.4–30.0^e^	29.4–30.0^f^	27.0	27.2
20	129.8	29.4–30.0^c^	28.4–29.6^d^	29.4–30.0^e^	29.4–30.0^f^	129.8	129.8
21	129.9	29.4–30.0^c^	28.4–29.6^d^	27.0	29.4–30.0^f^	129.9	129.9
22	27.2	29.4–30.0^c^	28.4–29.6^d^	129.8	29.4–30.0^f^	27.7	27.2
23	32.0	27.0	32.0	129.9	27.0	32.1	29.8
24	22.4	129.8	22.8	27.2	129.8	22.5	31.9
25	14.0	129.9	14.1	32.0	129.9	14.1	22.7
26		27.2		22.3	27.2		14.1
27		32.0		14.0	32.0		
28		22.4			22.4		
29		14.0			14.0		
COO							166.5
OCH_3_							52.0

*^a^* 150 MHz, *^b^* 100 MHz, *^c–h^* Overlapping signals.

**Table 3:** ^1^H and ^13^C NMR Spectroscopic Data of Compounds **8** and **9**.

	8 (CD_3_OD)		9 (CD_3_OD)	
Position	*δ*_C_ *^a^*	*δ*_H_ (*J* in Hz)*^b^*	*δ*_C_ *^a^*	*δ*_H_ (*J* in Hz)*^b^*
1	134.1		131.5	
2	104.6^c^	6.52 s^d^	112.3	7.09 brs
3	149.3		147.4	
4	135.9		150.5	
5	149.3		120.6	7.06 d (8.2)
6	104.6^c^	6.52 s^d^	121.1	6.96 dd (2.2, 8.2)
7	85.1	4.63 d (7.3)	74.9	4.85 overlap
8	53.7	1.89 m	85.9	4.36 m
9	62.3	3.87 m	62.2	3.82 m
		3.63 m		3.47 m
1′	139.5		137.8	
2′	112.2	6.92 d (1.8)	118.7^g^	6.86 brs^g^
3′	150.7		149.2	
4′	147.5		118.7^g^	6.86 brs^g^
5′	117.5	7.11 d (8.2)	151.2	
6′	120.9	6.82 dd (8.2, 1.8)	111.3	6.97 d (2.2)
7′	74..8	4.52 d (8.2)	132.9	6.52 d (15.4)
8′	50.7	2.53 m	111.3	6.27 dd (5.7, 15.4)
9′	76.2	4.26 dd (9.0, 4.6)	63.8	4.19 d (5.9)
		3.92 m		
3-OMe	56.9	3.84 s^e^	56.7	3.81 s
5-OMe	56.8	3.84 s^e^	56.5	3.79 s
3′-OMe	56.7	3.83 s		
Glc-1	102.7	4.84 m	103.1	4.81 d (6.9)
Glc-2	74.9	3.4–3.8 m^f^	73.9	3.4–3.8 m^h^
Glc-3	78.2	3.4–3.8 m^f^	78.2	3.4–3.8 m^h^
Glc-4	71.4	3.4–3.8 m^f^	71.4	3.4–3.8 m^h^
Glc-5	77.9	3.4–3.8 m^f^	77.9	3.4–3.8 m^h^
Glc-6	62.5	3.4–3.8 m^f^	62.5	3.4–3.8 m^h^

*^a^* 100 Hz, *^b^* 400 Hz, *^c^*^–*h*^ Overlapping signals.

**Table 4:** Antiproliferative Activity of the Isolated Compounds.

Compounds	Cell Lines*^a^* (IC_50_ μM)*^b^*				
	A549	MDA-MB-231	MCF-7	KB	KB-VIN
**1**	>40	22.5	>40	25.7	31.7
**2**	21.9	24.6	24.6	22.6	21.4
**8**	35.1	35.3	35.7	32.7	21.7
**10**	12.5	10.8	8.8	18.6	8.8
**13**	32.8	37.7	33.5	>40	8.7
**14**	23.2	32.9	32.8	23.2	19.9
**15**	8.1	20.8	6.8	20.3	5.4
**16**	5.7	8.2	8.1	12.6	5.3
**17**	37.8	>40	38.0	>40	8.2
**18**	>40	>40	>40	>40	>40
Paclitaxel (nM)	6.5	8.4	12.1	7.1	2213

*^a^* A549 (lung carcinoma), MDA-MB-231 (triple-negative breast cancer), MCF-7 (estrogen receptor-positive & HER2-negative breast cancer), KB (cervical cancer cell line HeLa derivative), KB-VIN (P-gp-overexpressing multidrug-resistant (MDR) subline of KB). *^b^* Antiproliferative activity expressed as IC_50_ values for each cell line cultured with compound for 72 h, the concentration of compound that caused 50% reduction relative to untreated cells determined by the SRB assay. IC_50_ of all compounds were calculated.

## Supplementary Materials

NMR spectra and HRMS of new compounds are available online at https://www.mdpi.com/1420-3049/24/21/4005/s1.
