# Supplementary material for: Isolation, Structure Elucidation, and Antiproliferative Activity of Butanolides and Lignan Glycosides from the Fruit of Hernandia nymphaeifolia
Source: Molecules. 2019 Nov 5;24(21):4005. doi: 10.3390/molecules24214005 (PMC6864620; doi:10.3390/molecules24214005)
Supplement: Supplementary file 1 [file molecules-24-04005-s001.pdf]

Isolation, Structure Elucidation, and Antiproliferative Activity of Butanolides and Lignan Glycosides from the Fruit of *Hernandia nymphaeifolia*

Simayijiang Aimaiti,[1] Yohei Saito,[1] Shuichi Fukuyoshi,[1] Masuo Goto,[2] Katsunori Miyake,[3] David J. Newman,[4] Barry R. O'Keefe,[5,6] Kuo-Hsiung Leeb,[2] and Kyoko Nakagawa-Goto[1,2,]*

[1] School of Pharmaceutical Sciences, College of Medical, Pharmaceutical and Health Sciences, Kanazawa University, Kanazawa, 920-1192, Japan

[2] Natural Products Research Laboratories, UNC Eshelman School of Pharmacy, University of North Carolina at Chapel Hill, Chapel Hill, North Carolina 27599-7568, United States

[3] Tokyo University of Pharmacy and Life Sciences, Hachioji, Tokyo, 192-0392, Japan

[4] NIH Special Volunteer, Wayne, Pennsylvania 19087, United States

[5] Natural Products Branch, Developmental Therapeutics Program, Division of Cancer Treatment and Diagnosis, National Cancer Institute, NCI at Frederick, Frederick, Maryland 21702-1201, United States

[6] Molecular Targets Program, Center for Cancer Research, National Cancer Institute, NCI at Frederick, Frederick, Maryland 21702-1201, United States

[7] Chinese Medicine Research and Development Center, China Medical University and Hospital, Taichung, 40447, Taiwan

You created this PDF from an application that is not licensed to print to novaPDF printer (http://www.novapdf.com)

Table of Contents

Figure S1. ^{1}H NMR spectrum of **1** (600MHz, in $CDCl_3$). 1
Figure S2. ^{13}C NMR spectrum of **1** (150MHz, in $CDCl_3$). 2
Figure S3. H-H COSY experiment of **1** (600MHz, in $CDCl_3$) 3
Figure S4. HMQC experiment of **1** (600MHz, in $CDCl_3$). 4
Figure S5. HMBC experiment of **1** (600MHz, in $CDCl_3$)... 5
Figure S6. NOESY experiment of **1** (600MHz, in $CDCl_3$). 6
Figure S7. ^{1}H NMR spectrum of **2** (400MHz, in $CDCl_3$). 7
Figure S8. ^{13}C NMR spectrum of **2** (100MHz, in $CDCl_3$). 8
Figure S9. H-H COSY experiment of **2** (400MHz, in $CDCl_3$). 9
Figure S10. HMQC experiment of **2** (400MHz, in $CDCl_3$)...................................... 10
Figure S11. HMBC experiment of **2** (400MHz, in $CDCl_3$)...................................... 11
Figure S12. NOESY experiment of **2** (400MHz, in $CDCl_3$)..................................... 12
Figure S13. ^{1}H NMR spectrum of **3** (400MHz, in $CDCl_3$)....................................... 13
Figure S14. ^{13}C NMR spectrum of **3** (100MHz, in $CDCl_3$) 14
Figure S15. H-H COSY experiment of **3** (400MHz, in $CDCl_3$)................................ 15
Figure S16. HMQC experiment of **3** (400MHz, in $CDCl_3$)...................................... 16
Figure S17. HMBC experiment of **3** (400MHz, in $CDCl_3$)...................................... 17
Figure S18. NOESY experiment of **3** (400MHz, in $CDCl_3$) 18
Figure S19. ^{1}H NMR spectrum of **4** (400MHz, in $CDCl_3$)....................................... 19
Figure S20.^{13}C NMR spectrum of **4** (100MHz, in $CDCl_3$). 20
Figure S21. COSY experiment of **4** (400MHz, in $CDCl_3$).. 21
Figure S22 HMQC experiment of **4** (400MHz, in $CDCl_3$)....................................... 22
Figure S23. HMBC experiment of **4** (400MHz, in $CDCl_3$)...................................... 23
Figure S24. NOESY experiment of **4** (400MHz, in $CDCl_3$)..................................... 24
Figure S25. ^{1}H NMR spectrum of **5** (400MHz, in $CDCl_3$)....................................... 25
Figure S26. ^{13}C NMR spectrum of **5** (100MHz, in $CDCl_3$)...................................... 26
Figure S27. H-H COSY experiment of **5** (400MHz, in $CDCl_3$)................................ 27
Figure S28. HMQC experiment of **5** (400MHz, in $CDCl_3$)...................................... 28
Figure S29. HMBC experiment of **5** (400MHz, in $CDCl_3$)...................................... 29
Figure S30. NOESY experiment of **5** (400MHz, in $CDCl_3$) 30
Figure S31. ^{1}H NMR spectrum of **6** (600MHz, in $CDCl_3$) 31
Figure S32. ^{13}C NMR spectrum of **6** (150MHz, in $CDCl_3$) 32
Figure S33. H-H COSY experiment of **6** (600MHz, in $CDCl_3$)................................ 33
Figure S34. HMQC experiment of **6** (600MHz, in $CDCl_3$)...................................... 34

You created this PDF from an application that is not licensed to print to novaPDF printer (http://www.novapdf.com)

Figure S35. HMBC experiment of **6** (600MHz, in $CDCl_3$)...................................... 35
Figure S36. NOESY experiment of **6** (600MHz, in $CDCl_3$)..................................... 36
Figure S37. ^{1}H NMR spectrum of **7** (400MHz, in $CDCl_3$)...................................... 37
Figure S38. ^{13}C NMR spectrum of **7** (100MHz, in $CDCl_3$)..................................... 38
Figure S39. H-H COSY experiment of **7** (400MHz, in $CDCl_3$)................................ 39
Figure S40. HMQC experiment of **7** (400MHz, in $CDCl_3$)..................................... 40
Figure S41. HMBC experiment of **7** (400MHz, in $CDCl_3$)...................................... 41
Figure S42. NOESY experiment of **7** (400MHz, in $CDCl_3$)..................................... 42
Figure S43. EIMS spectrum for **7** 43
Figure S44. ^{1}H MNR spectrum of **8** (400MHz, in CD_3OD).................................... 44
Figure S45. ^{13}C MNR spectrum of **8** (100MHz, in CD_3OD)................................... 45
Figure S46. H-H COSY experiment of **8** (400MHz, in CD_3OD).............................. 46
Figure S47. HMQC experiment of **8** (400MHz, in CD_3OD).................................... 47
Figure S48. HMBC experiment of **8** (400MHz, in CD_3OD) 48
Figure S49. NOESY experiment of **8** (400MHz, in CD_3OD)................................... 49
Figure S50. ROESY experiment of **8** (400MHz, in CD_3OD) 50
Figure S51. ^{1}H MNR spectrum of **9** (400MHz, in CD_3OD).................................... 51
Figure S52. ^{13}C MNR spectrum of **9** (100MHz, in CD_3OD)................................... 52
Figure S53. H-H COSY experiment of **9** (400MHz, in CD_3OD).............................. 53
Figure S54. HMQC experiment of **9** (400MHz, in CD_3OD).................................... 54
Figure S56. NOESY experiment of **9** (400MHz, in CD_3OD).................................. 56
Figure S57. ^{1}H NMR spectrum of **9** (400MHz, in DMSO-d_6)................................ 57

Figure S58. HRFABMS data of **1**.. 58
Figure S59. HRFABMS data of **2**.. 58
Figure S60. HRFABMS data of **3**.. 59
Figure S61. HRFABMS data of **4**.. 59
Figure S62. HRFABMS data of **5**.. 60
Figure S63. HRFABMS data of **6**.. 60
Figure S64. HRFABMS data of **7**.. 61
Figure S65. HRFABMS data of **8**.. 61
Figure S66. HRFABMS data of **9**.. 62

You created this PDF from an application that is not licensed to print to novaPDF printer (http://www.novapdf.com)

Figure S1. ^{1}H NMR spectrum of **1** (600MHz, in $CDCl_3$).

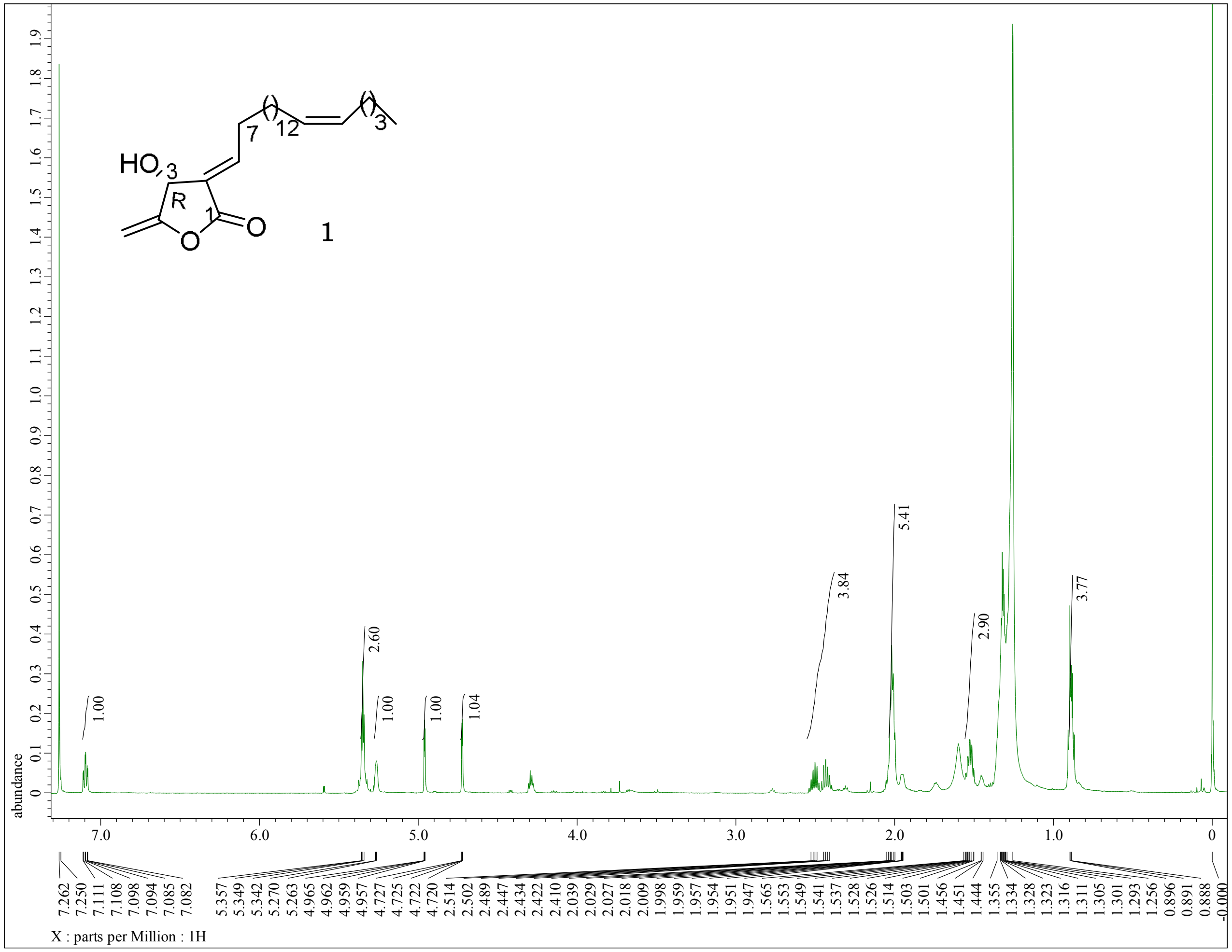

You created this PDF from an application that is not licensed to print to novaPDF printer (http://www.novapdf.com)

Figure S2. ^{13}C NMR spectrum of **1** (150MHz, in $CDCl_3$).

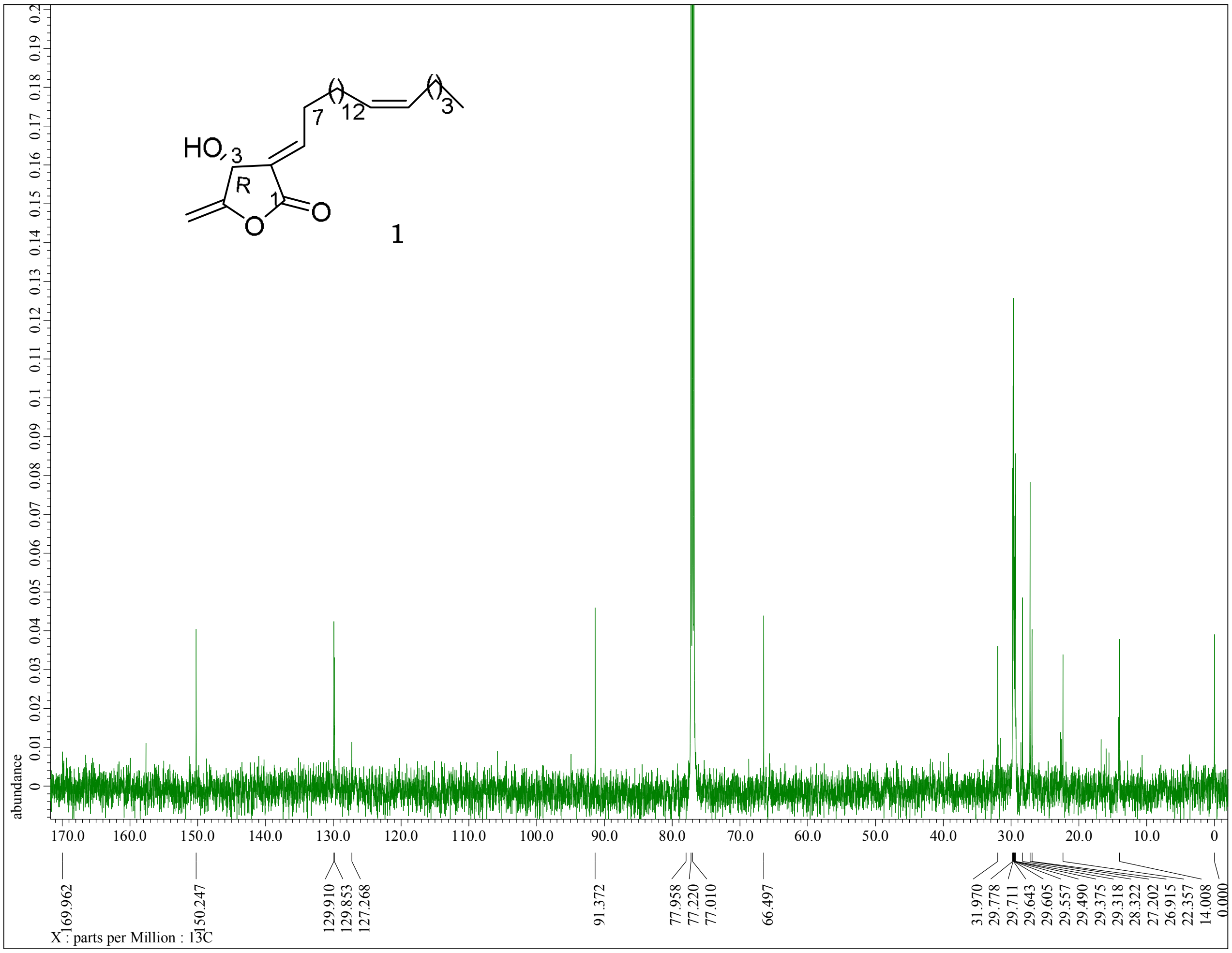

You created this PDF from an application that is not licensed to print to novaPDF printer (http://www.novapdf.com)

Figure S3. H-H COSY experiment of **1** (600MHz, in $CDCl_3$).

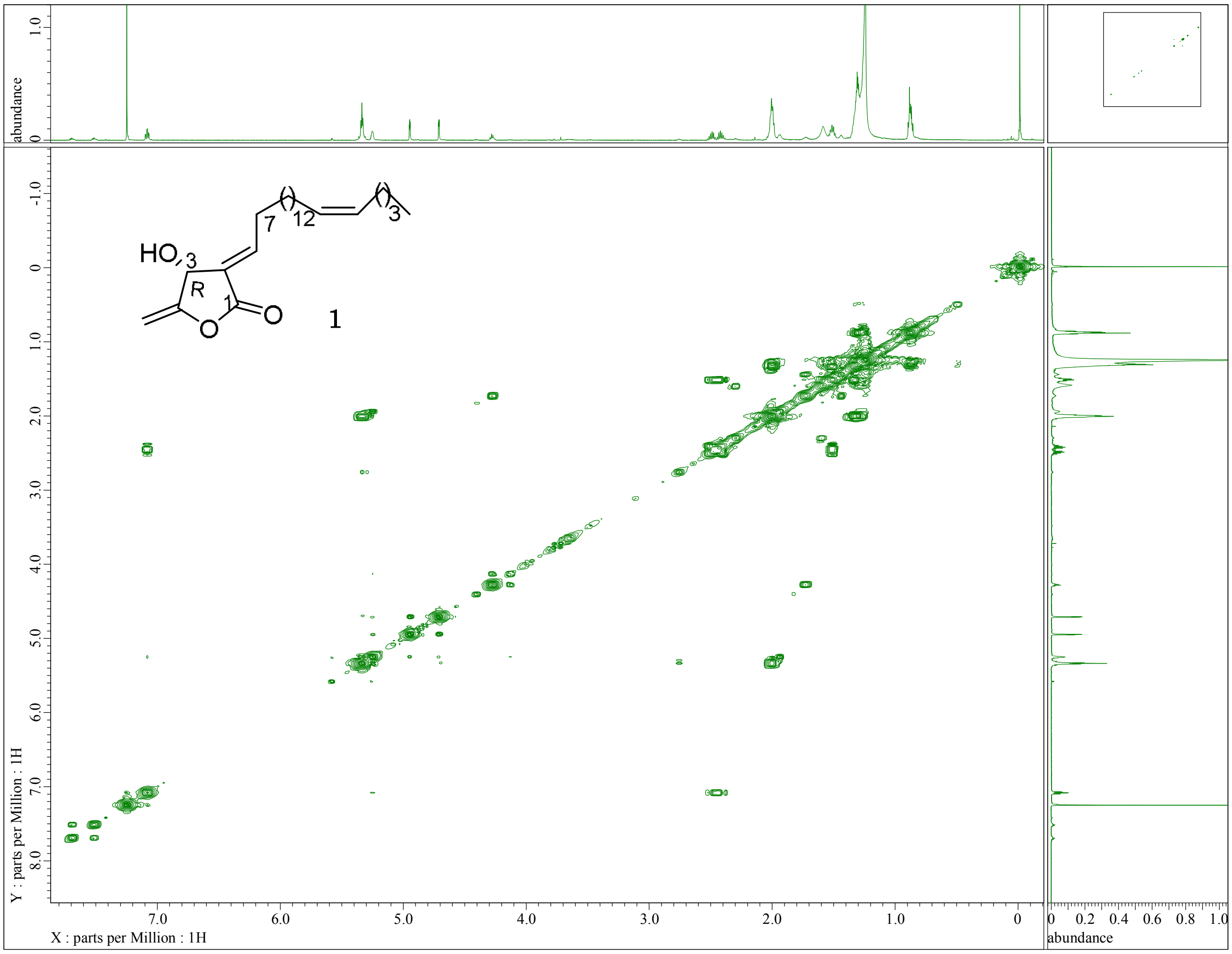

You created this PDF from an application that is not licensed to print to novaPDF printer (http://www.novapdf.com)

Figure S4. HMQC experiment of **1** (600MHz, in $CDCl_3$).

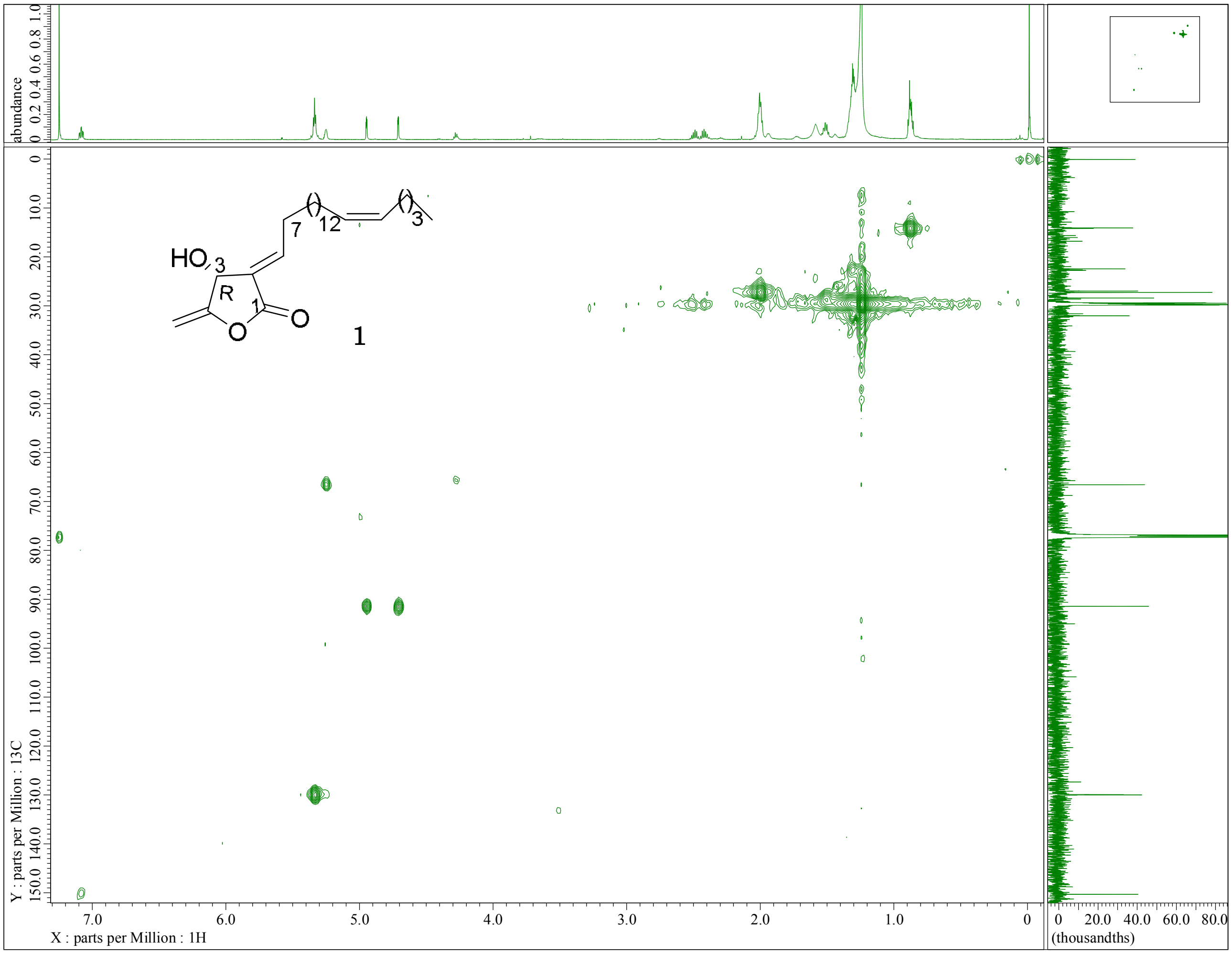

You created this PDF from an application that is not licensed to print to novaPDF printer (http://www.novapdf.com)

Figure S5. HMBC experiment of **1** (600MHz, in $CDCl_3$).

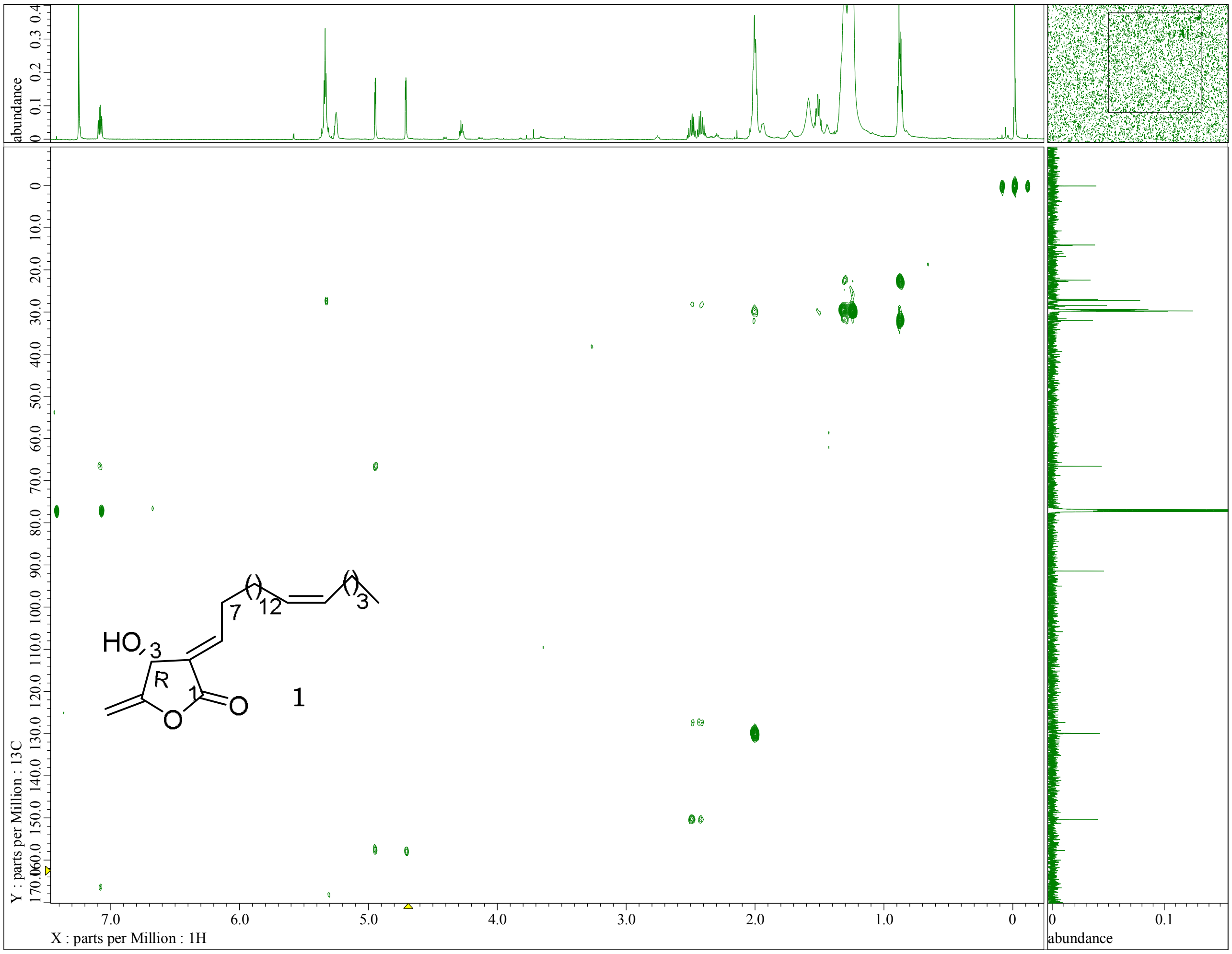

You created this PDF from an application that is not licensed to print to novaPDF printer (http://www.novapdf.com)

Figure S6. NOESY experiment of **1** (600MHz, in $CDCl_3$).

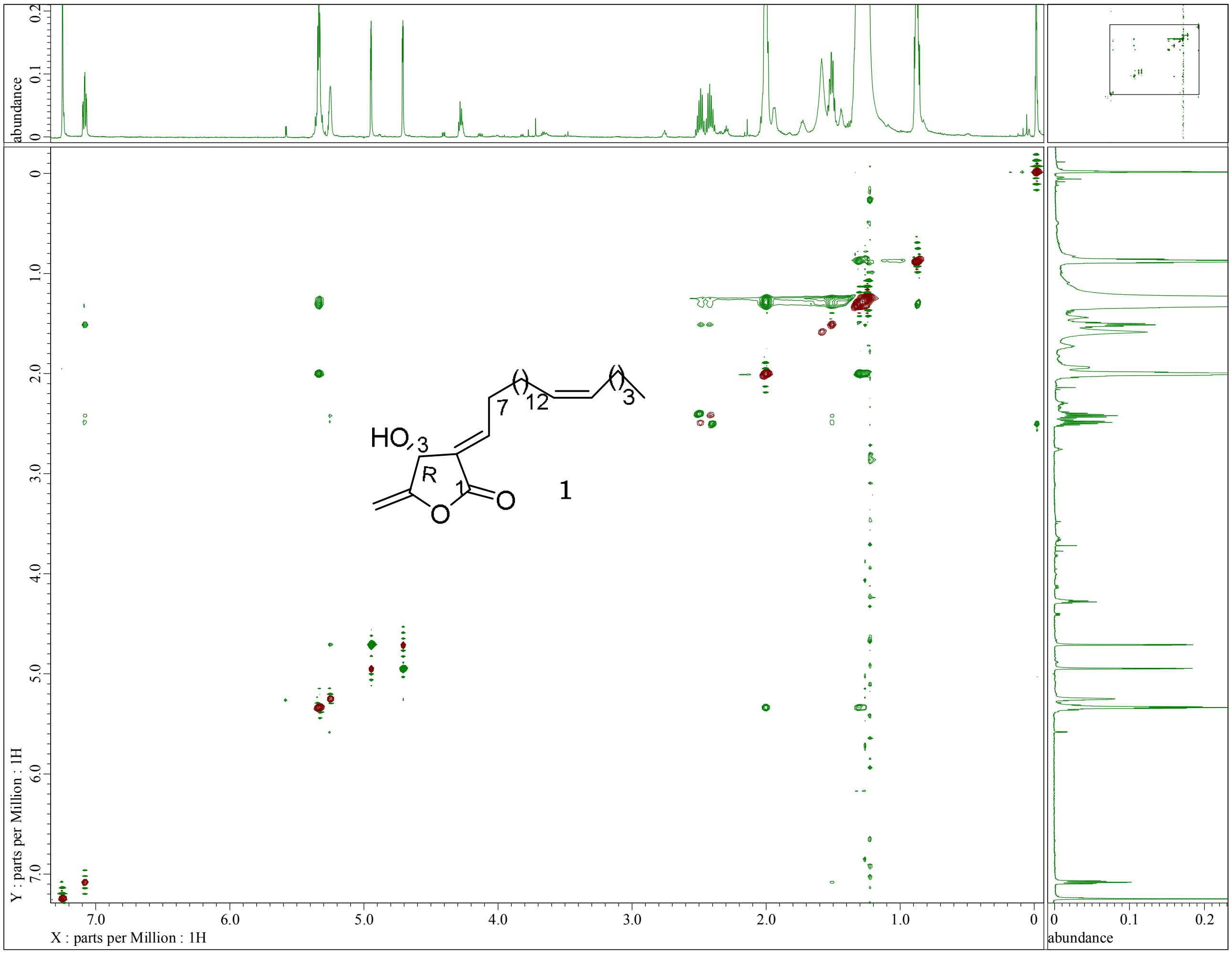

You created this PDF from an application that is not licensed to print to novaPDF printer (http://www.novapdf.com)

Figure S7. ^{1}H NMR spectrum of **2** (400MHz, in $CDCl_3$).

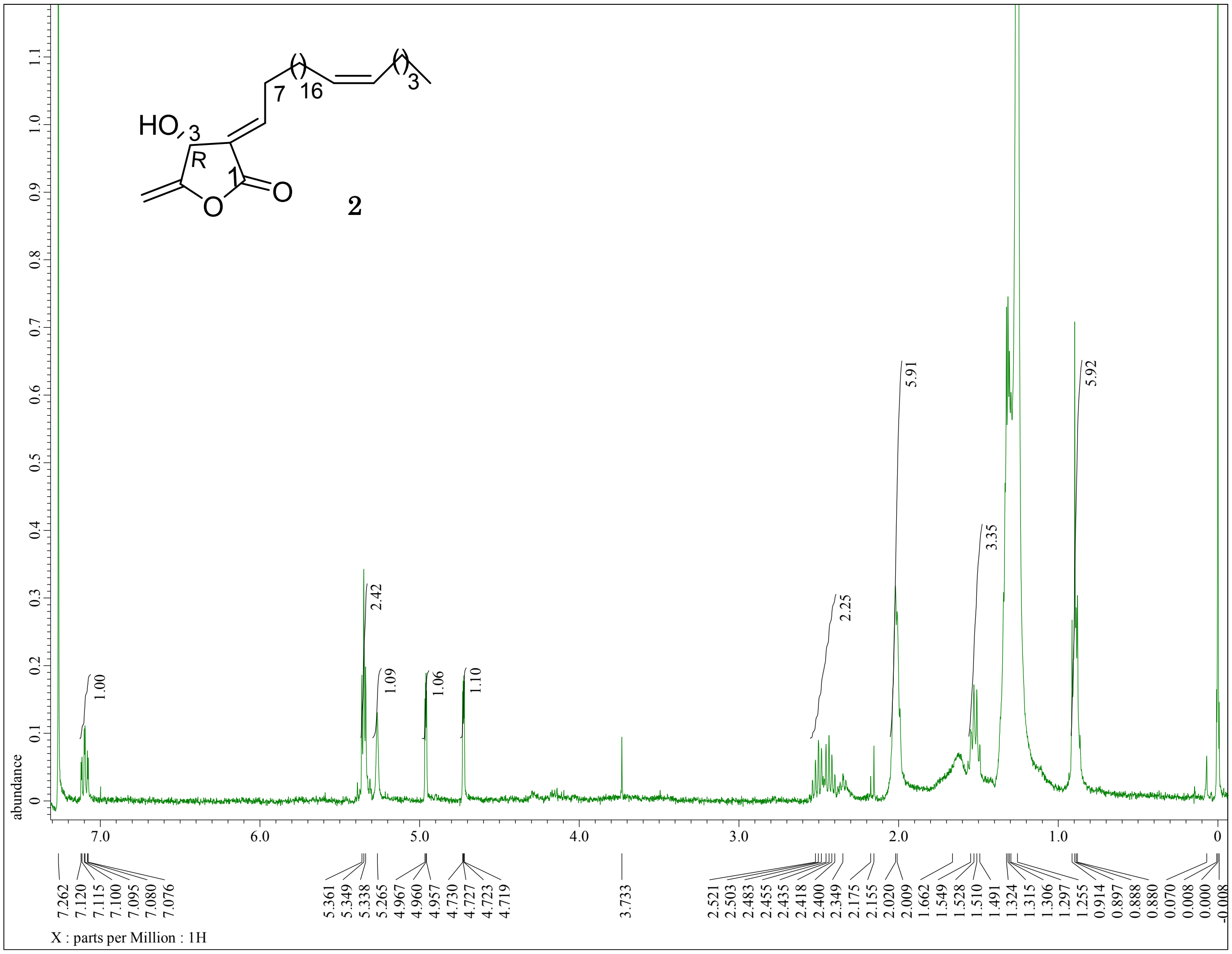

You created this PDF from an application that is not licensed to print to novaPDF printer (http://www.novapdf.com)

Figure S8. ^{13}C NMR spectrum of **2** (100MHz, in $CDCl_3$).

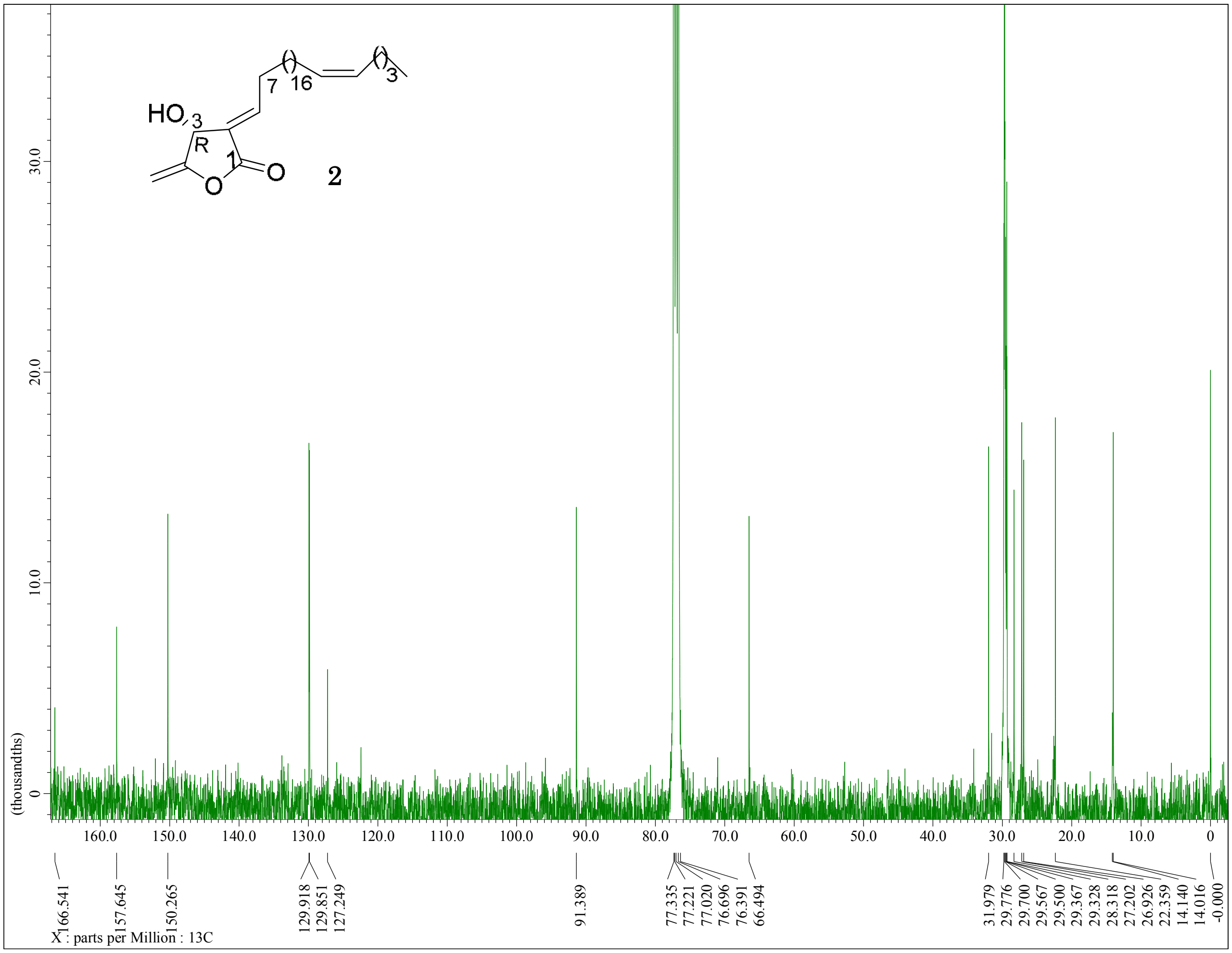

You created this PDF from an application that is not licensed to print to novaPDF printer (http://www.novapdf.com)

Figure S9. H-H COSY experiment of **2** (400MHz, in $CDCl_3$).

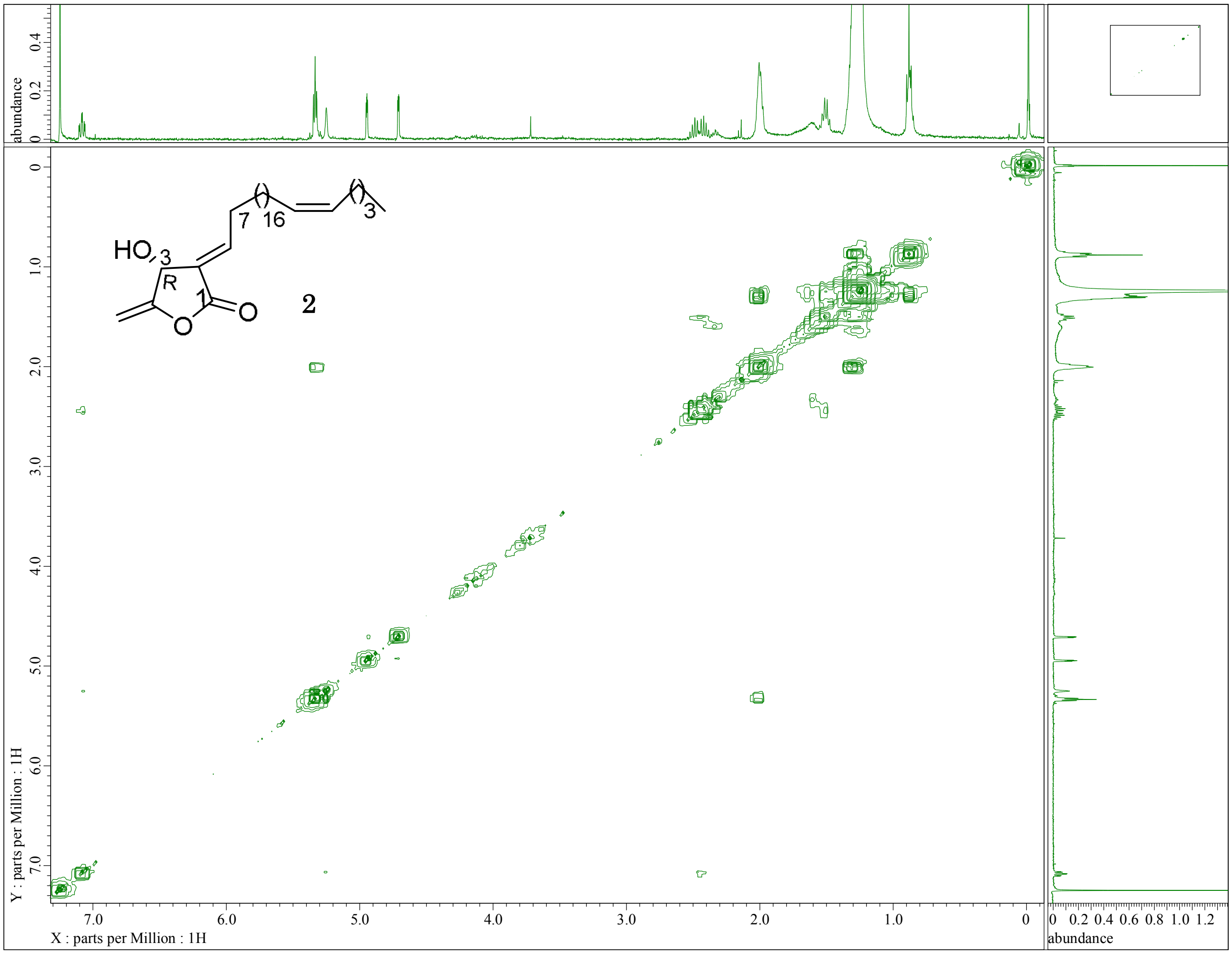

You created this PDF from an application that is not licensed to print to novaPDF printer (http://www.novapdf.com)

Figure S10. HMQC experiment of **2** (400MHz, in $CDCl_3$).

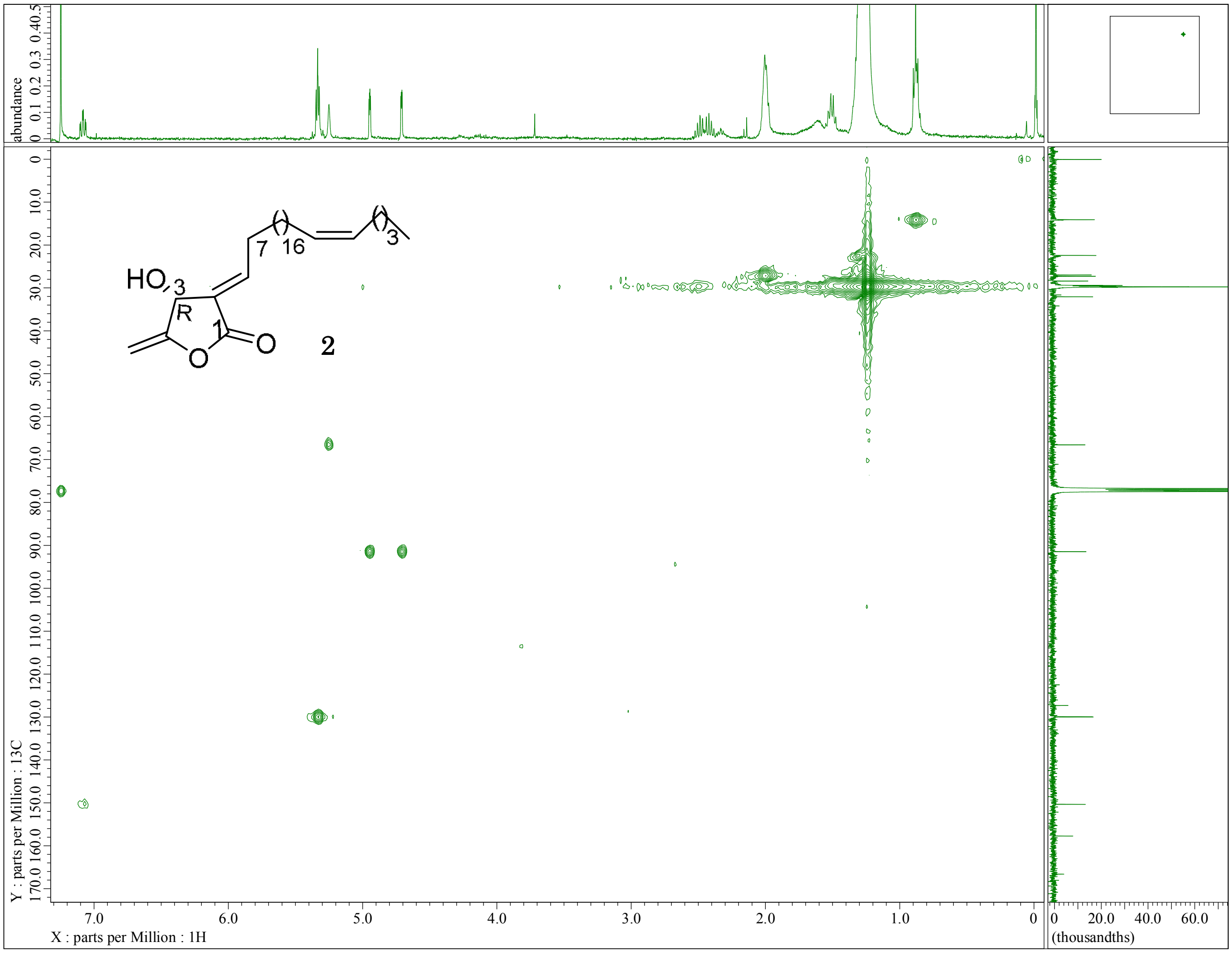

You created this PDF from an application that is not licensed to print to novaPDF printer (http://www.novapdf.com)

Figure S11. HMBC experiment of **2** (400MHz, in $CDCl_3$).

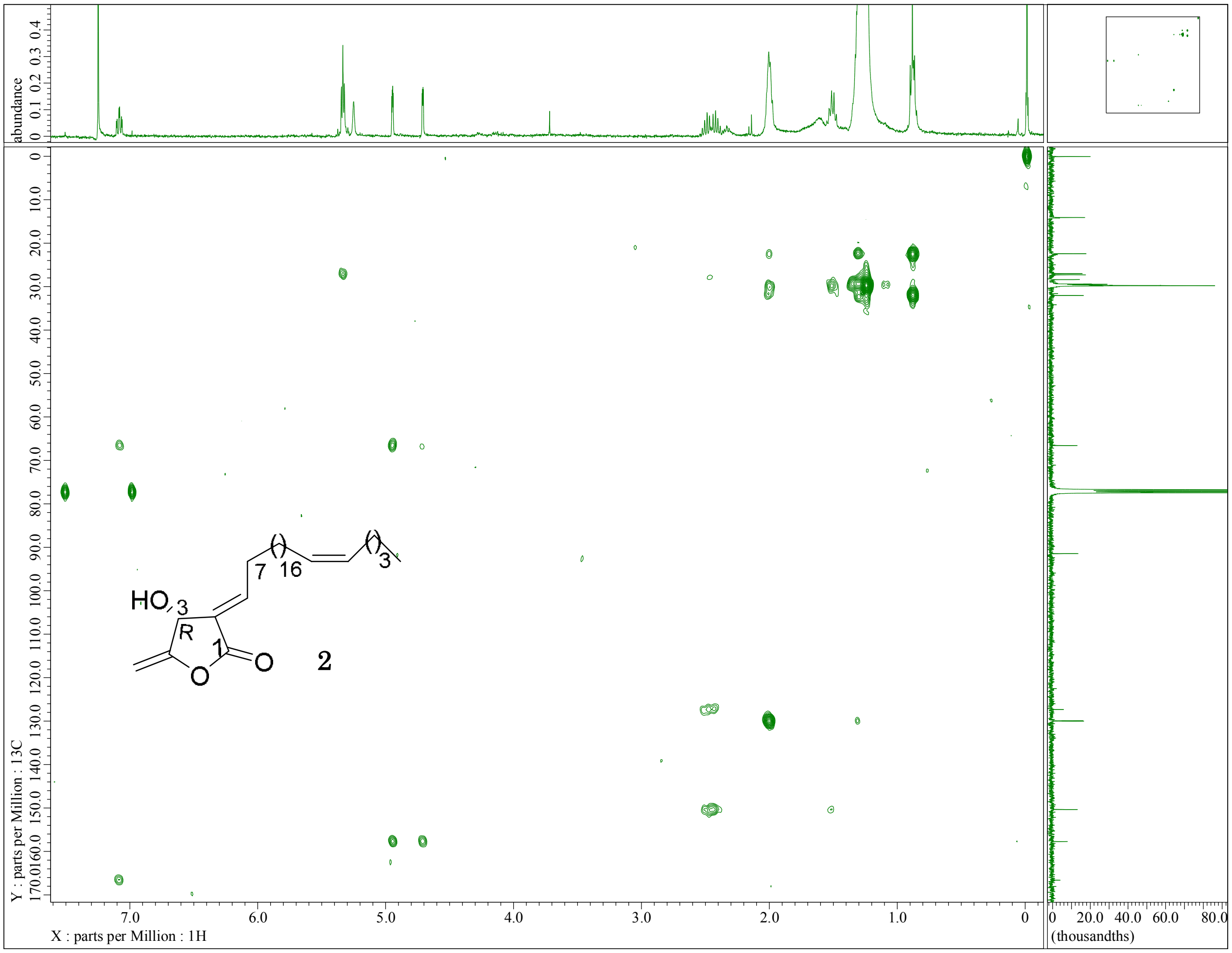

You created this PDF from an application that is not licensed to print to novaPDF printer (http://www.novapdf.com)

Figure S12. NOESY experiment of **2** (400MHz, in $CDCl_3$).

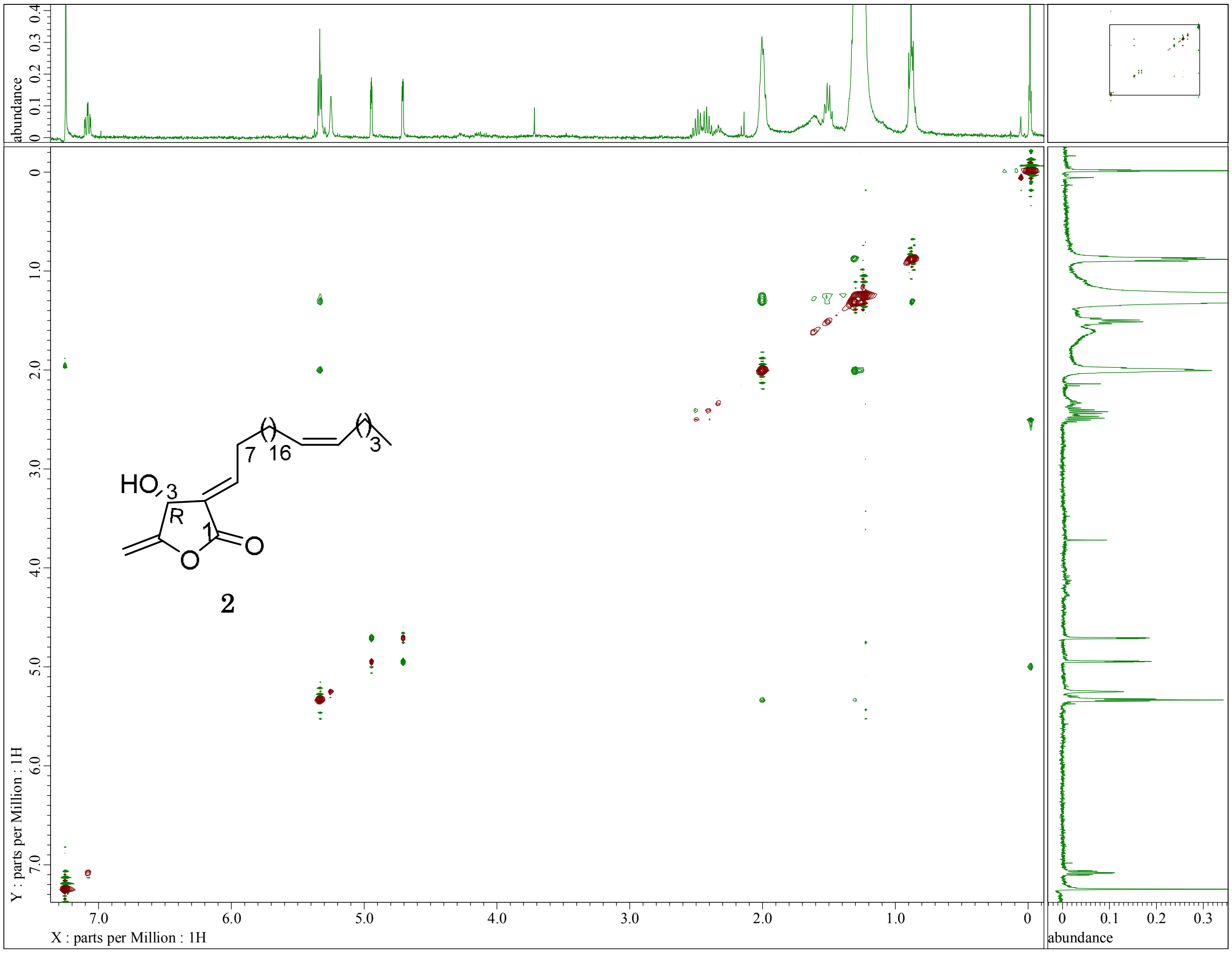

You created this PDF from an application that is not licensed to print to novaPDF printer (http://www.novapdf.com)

Figure S13. ^{1}H NMR spectrum of **3** (400MHz, in CDCl$_3$).

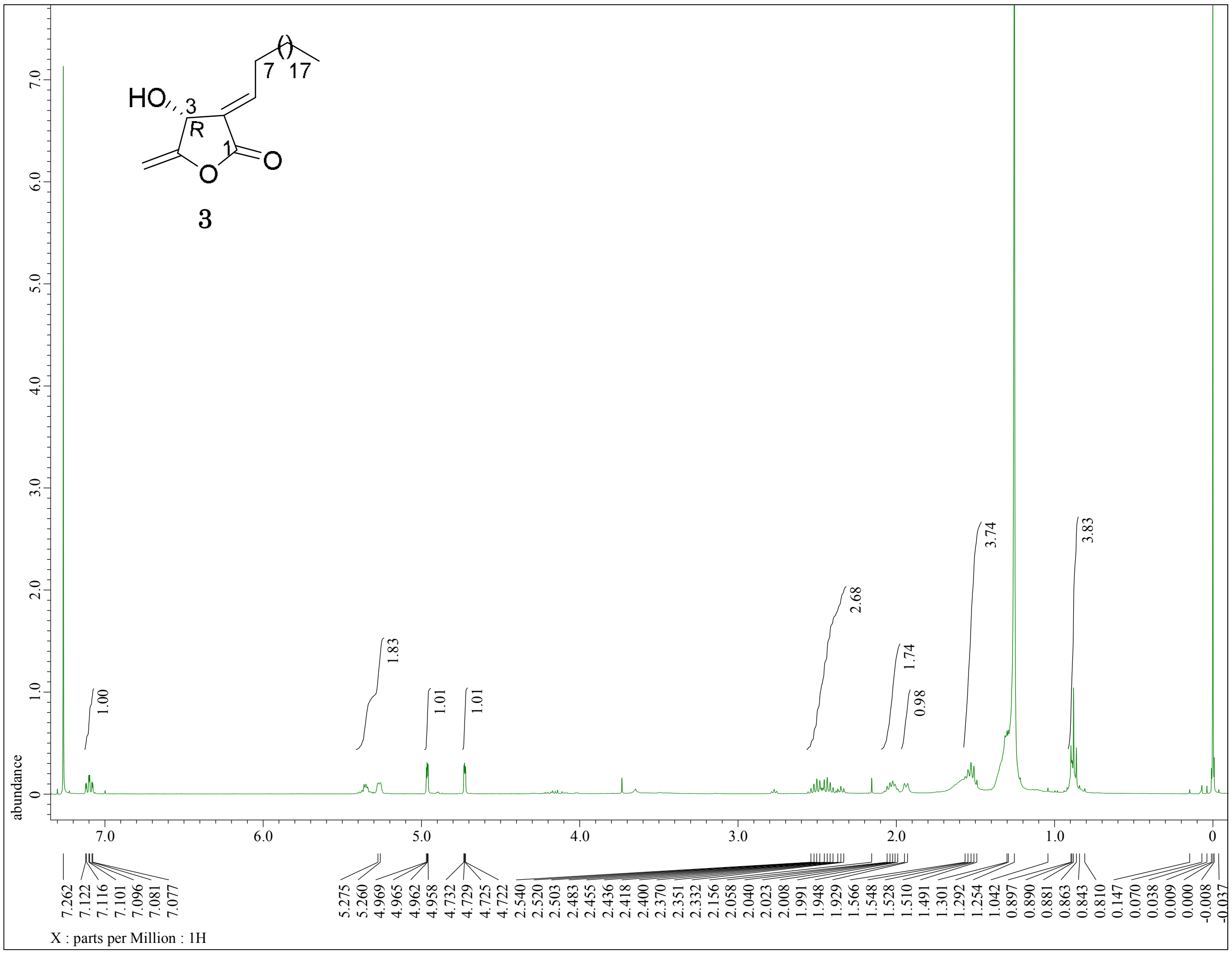

You created this PDF from an application that is not licensed to print to novaPDF printer (http://www.novapdf.com)

Figure S14. ^{13}C NMR spectrum of **3** (100MHz, in $CDCl_3$)

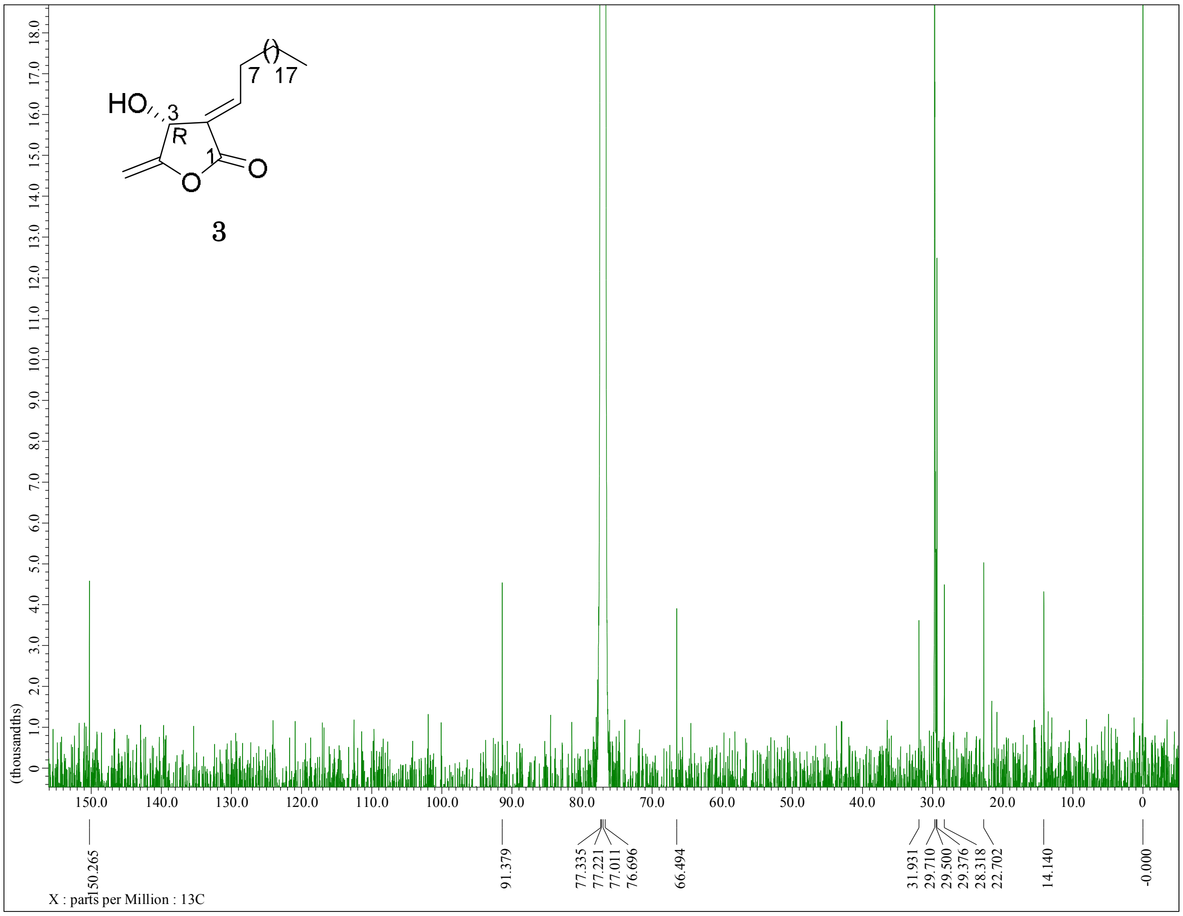

You created this PDF from an application that is not licensed to print to novaPDF printer (http://www.novapdf.com)

Figure S15. H-H COSY experiment of **3** (400MHz, in $CDCl_3$)

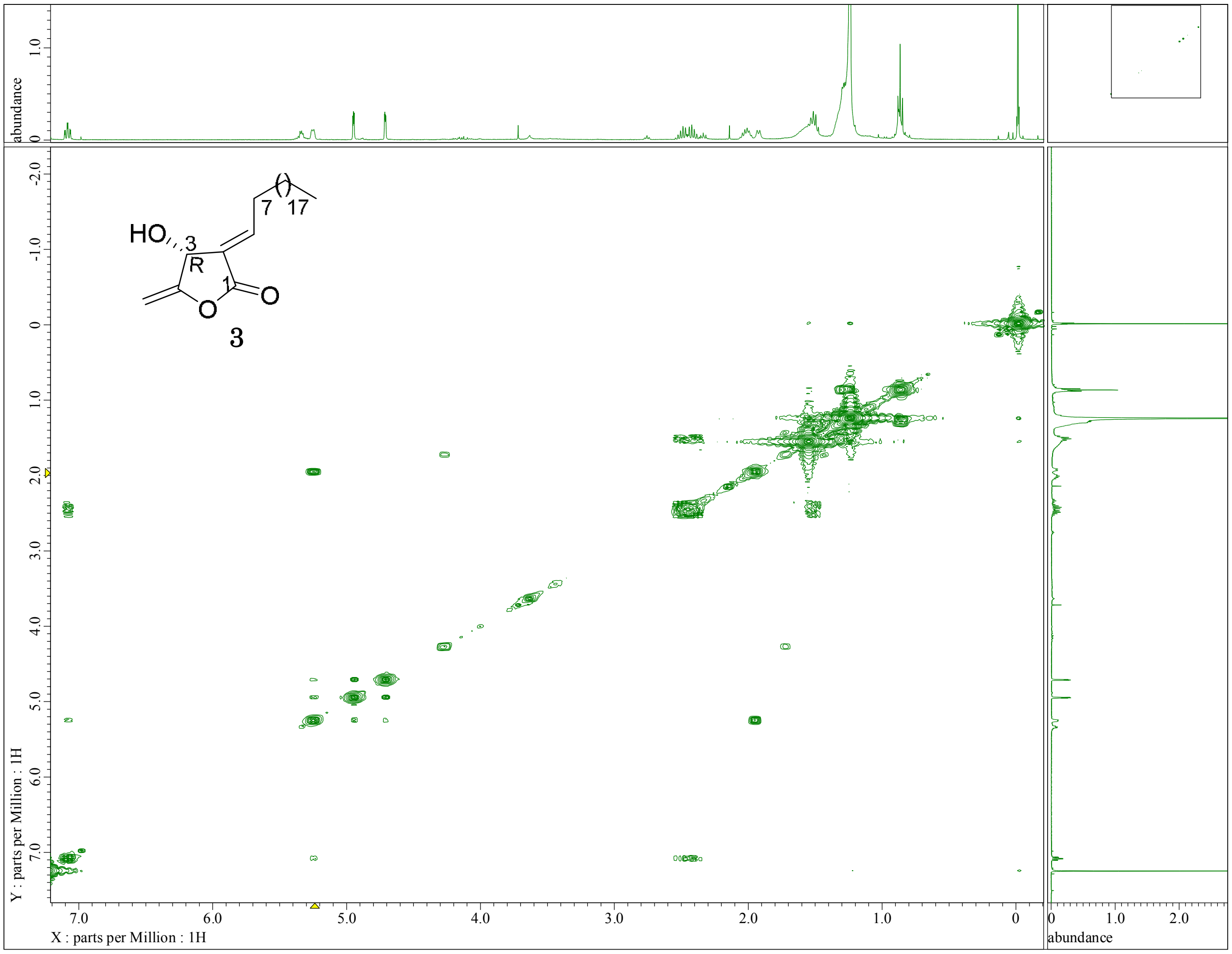

You created this PDF from an application that is not licensed to print to novaPDF printer (http://www.novapdf.com)

Figure S16. HMQC experiment of **3** (400MHz, in $CDCl_3$)

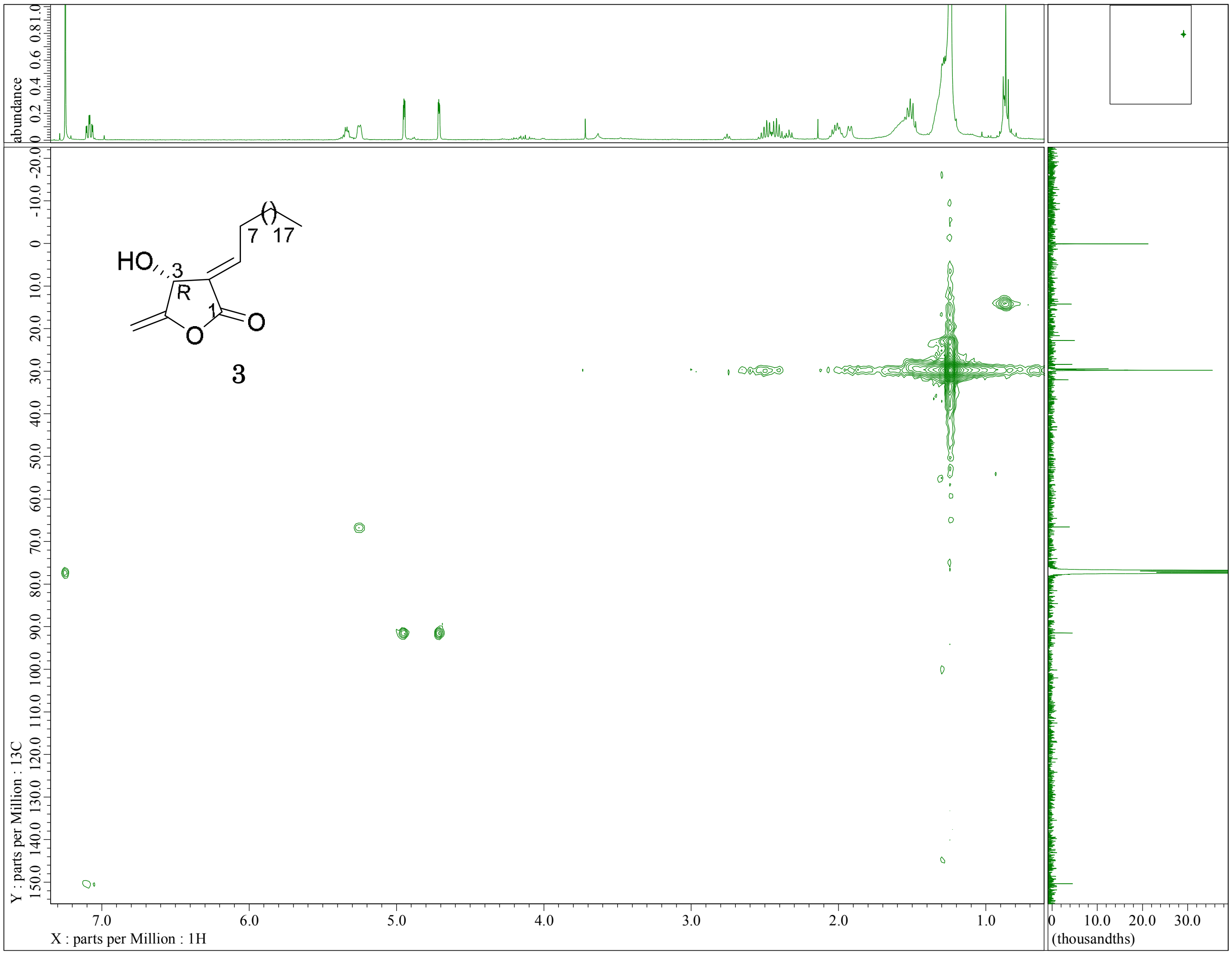

You created this PDF from an application that is not licensed to print to novaPDF printer (http://www.novapdf.com)

Figure S17. HMBC experiment of **3** (400MHz, in $CDCl_3$)

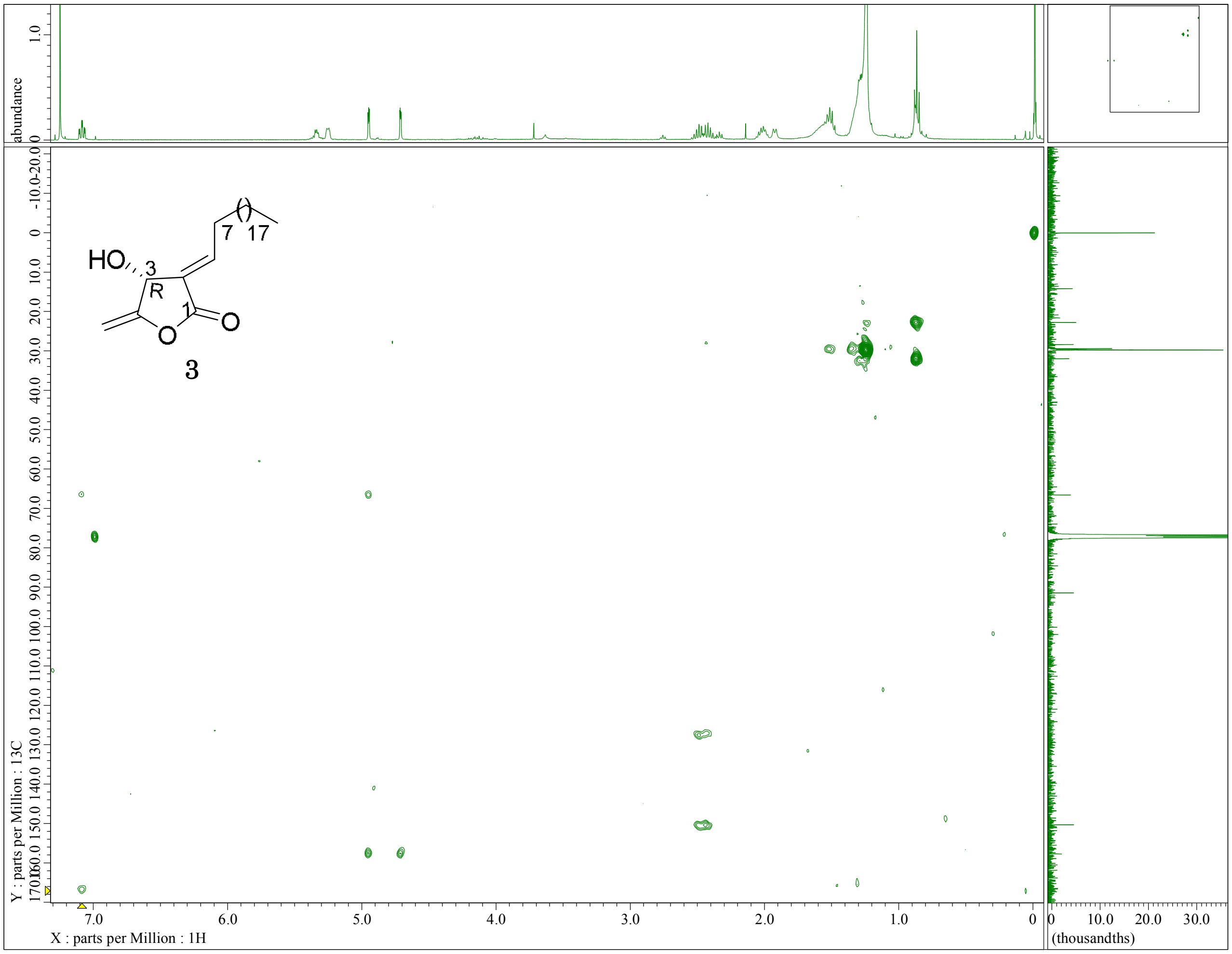

You created this PDF from an application that is not licensed to print to novaPDF printer (http://www.novapdf.com)

Figure S18. NOESY experiment of **3** (400MHz, in $CDCl_3$)

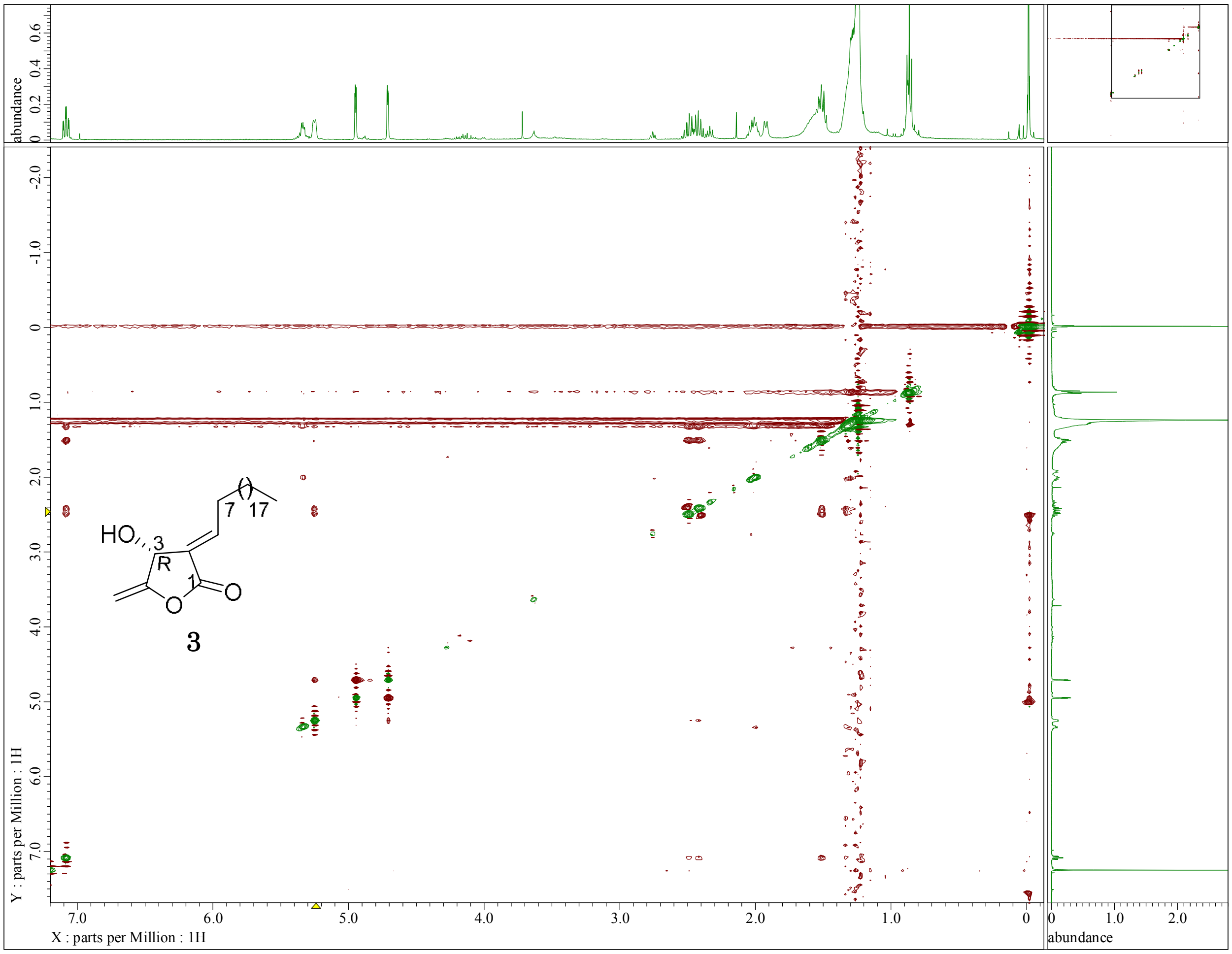

You created this PDF from an application that is not licensed to print to novaPDF printer (http://www.novapdf.com)

Figure S19. ^{1}H NMR spectrum of **4** (400MHz, in $CDCl_3$).

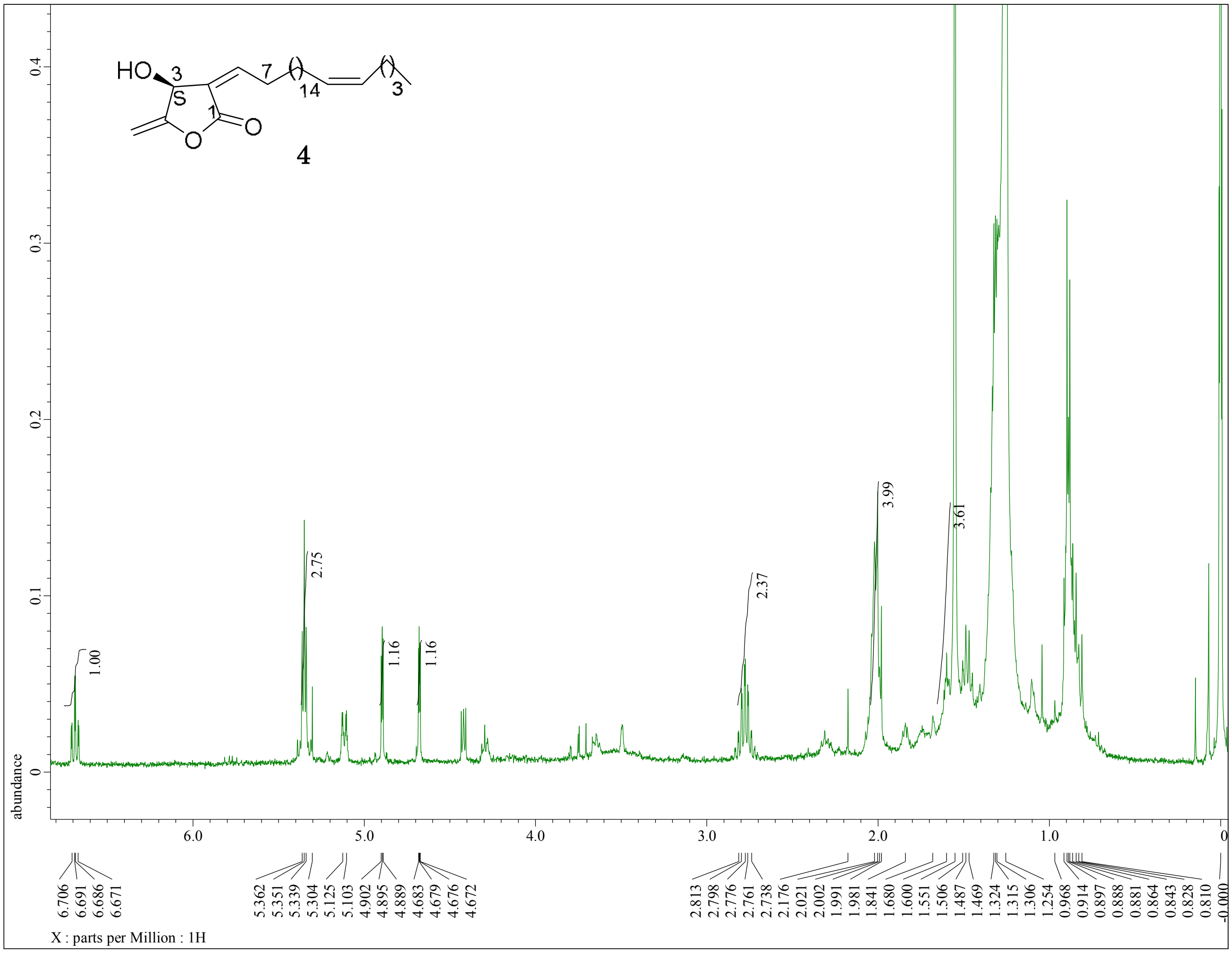

You created this PDF from an application that is not licensed to print to novaPDF printer (http://www.novapdf.com)

Figure S20.^{13}C NMR spectrum of **4** (100MHz, in $CDCl_3$).

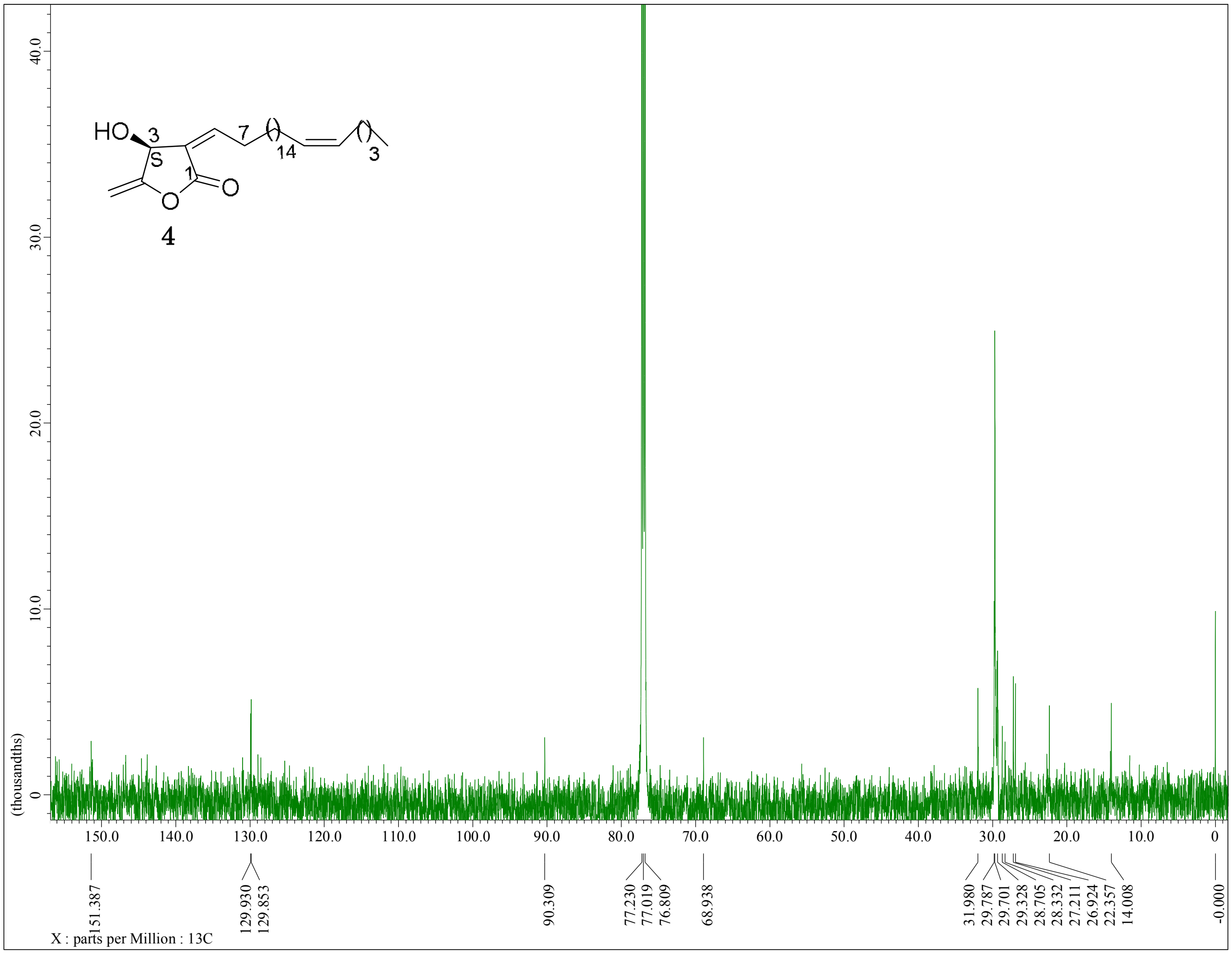

You created this PDF from an application that is not licensed to print to novaPDF printer (http://www.novapdf.com)

Figure S21. COSY experiment of **4** (400MHz, in $CDCl_3$).

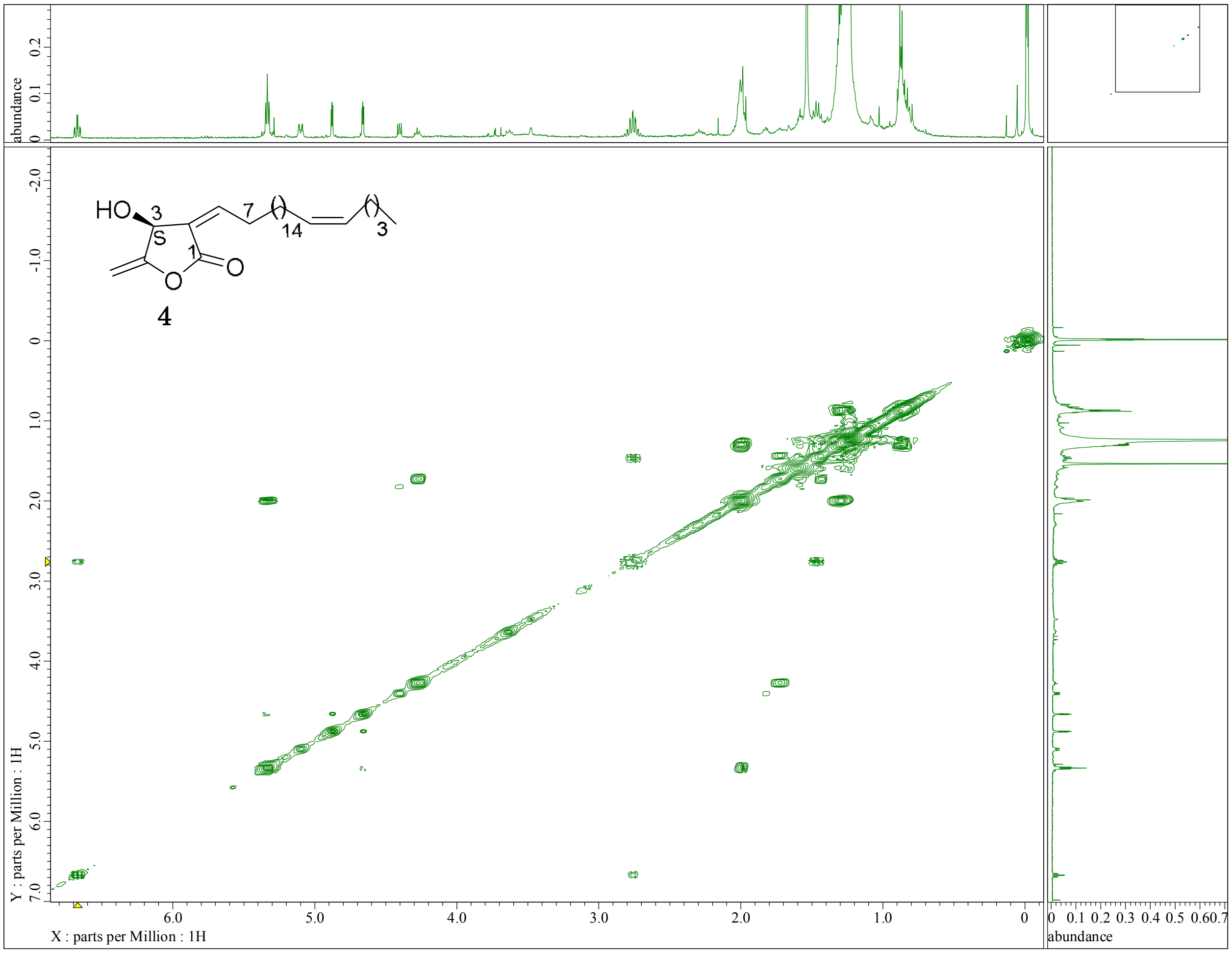

You created this PDF from an application that is not licensed to print to novaPDF printer (http://www.novapdf.com)

Figure S22 HMQC experiment of **4** (400MHz, in $CDCl_3$).

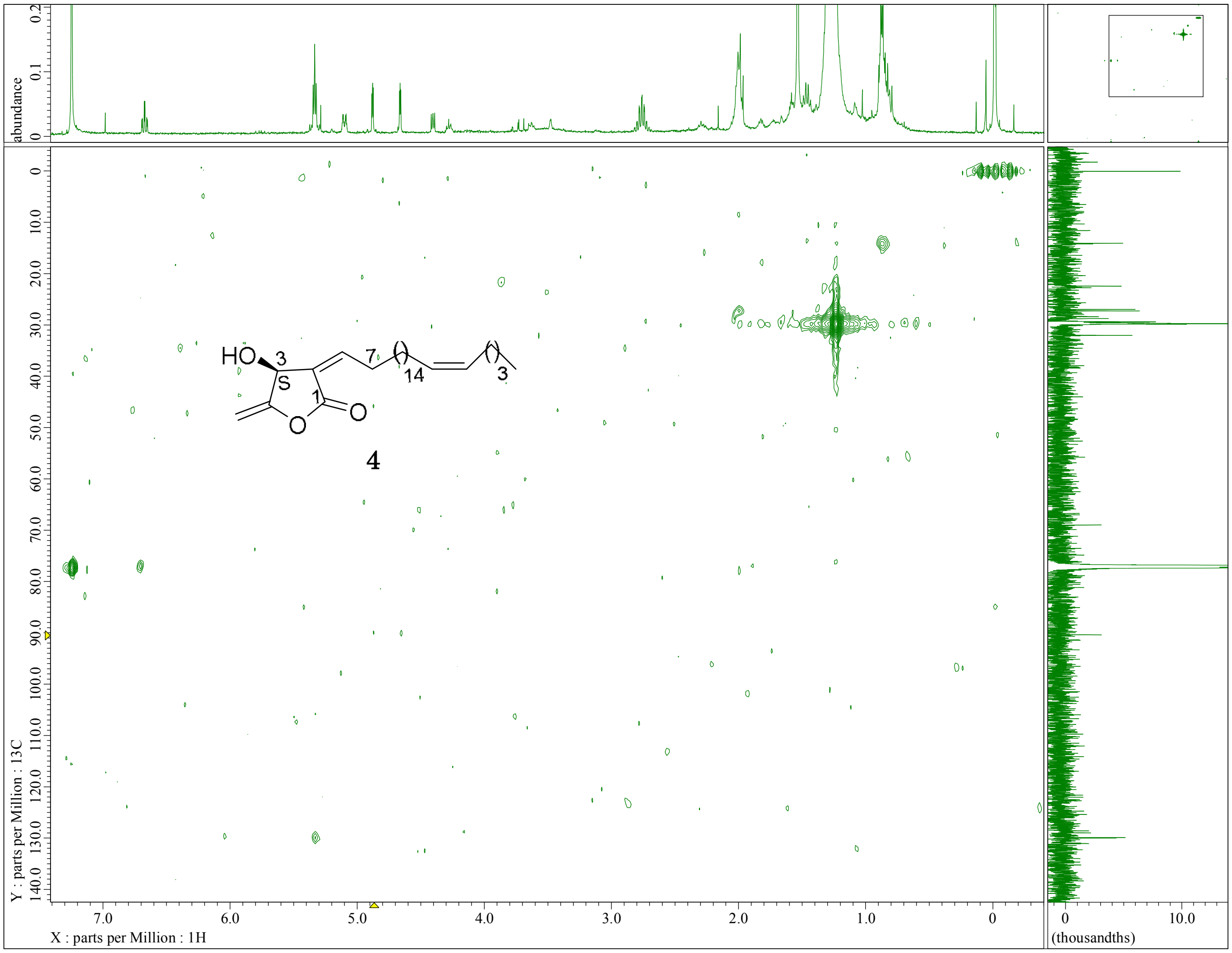

You created this PDF from an application that is not licensed to print to novaPDF printer (http://www.novapdf.com)

Figure S23. HMBC experiment of **4** (400MHz, in $CDCl_3$).

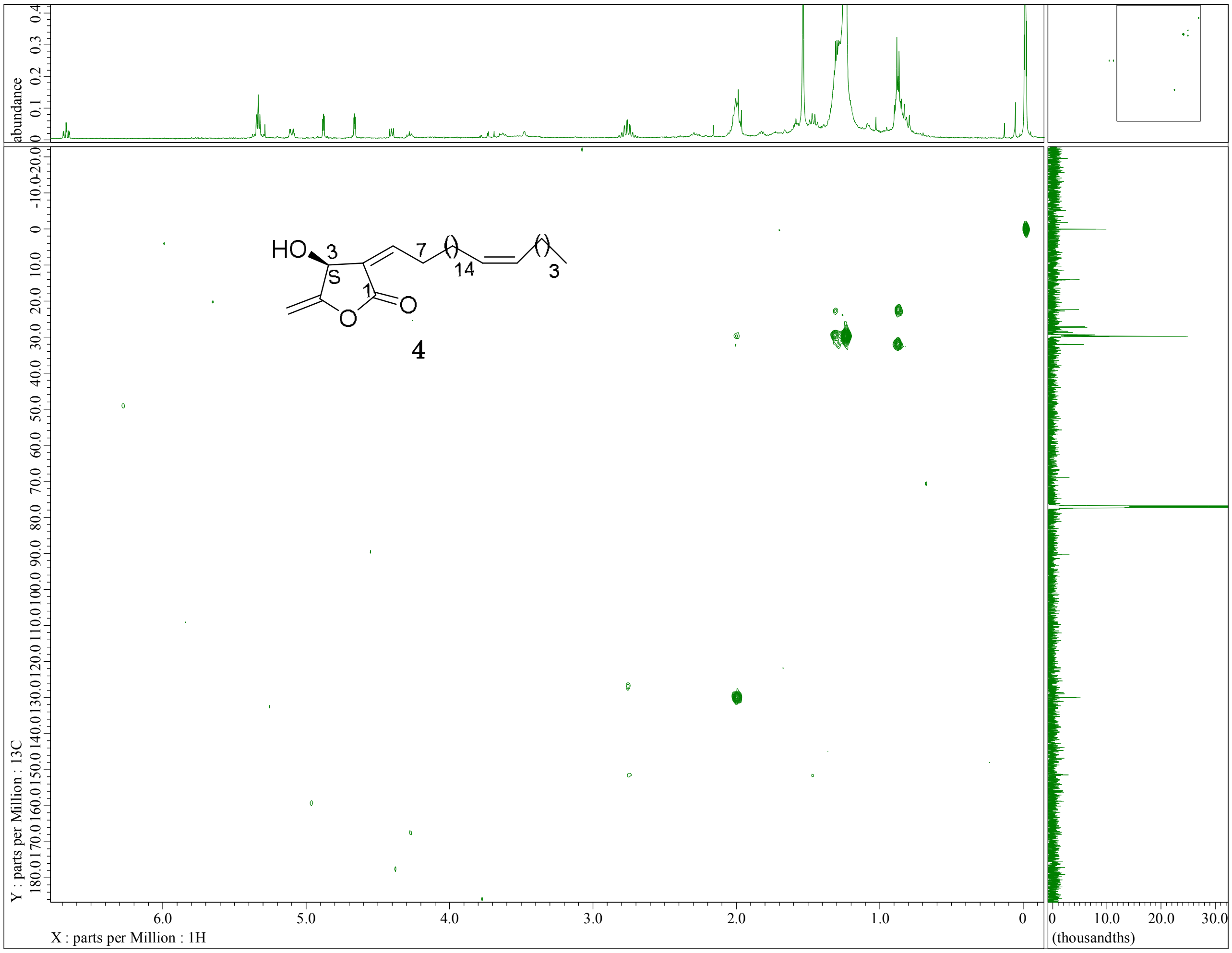

You created this PDF from an application that is not licensed to print to novaPDF printer (http://www.novapdf.com)

Figure S24. NOESY experiment of **4** (400MHz, in $CDCl_3$).

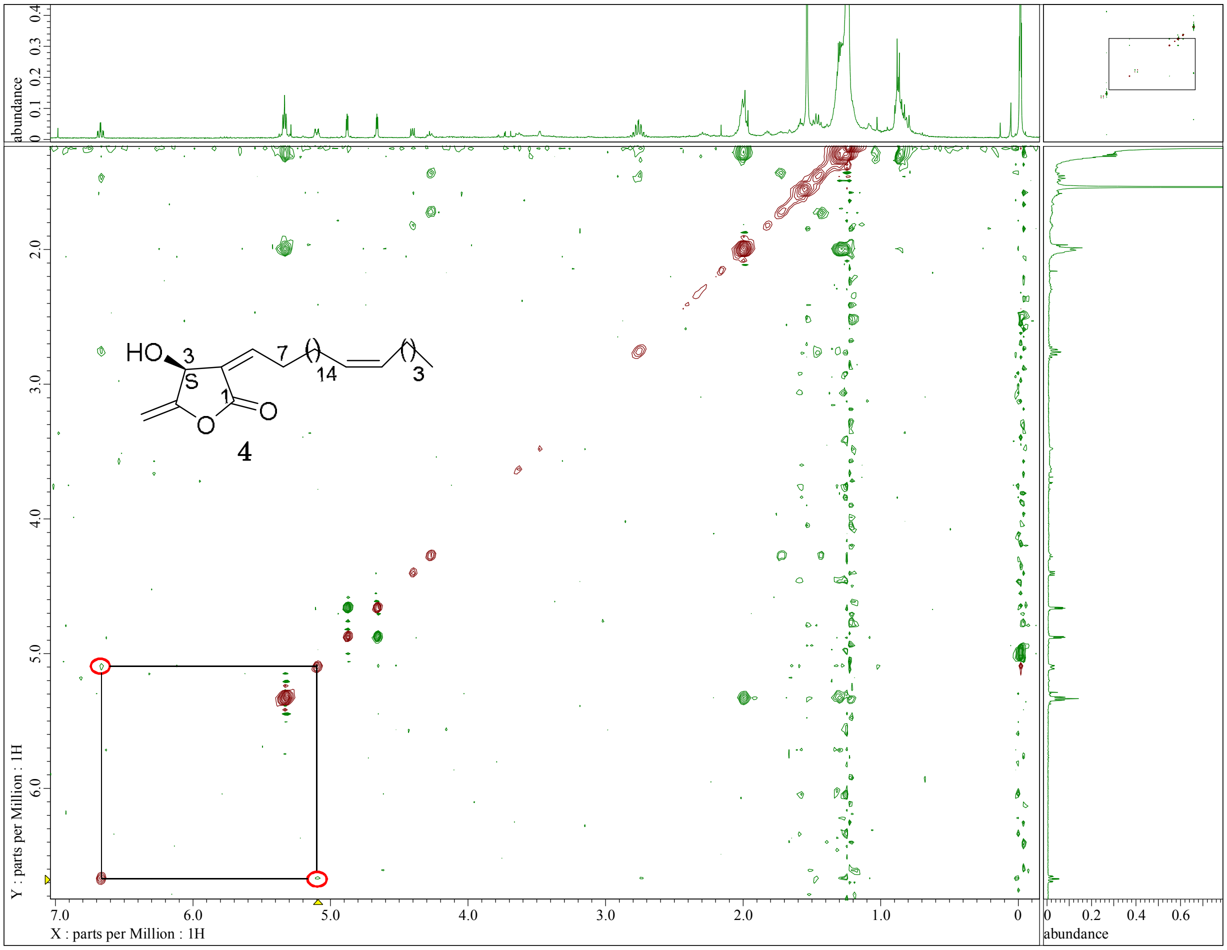

You created this PDF from an application that is not licensed to print to novaPDF printer (http://www.novapdf.com)

Figure S25. ^{1}H NMR spectrum of **5** (400MHz, in $CDCl_3$).

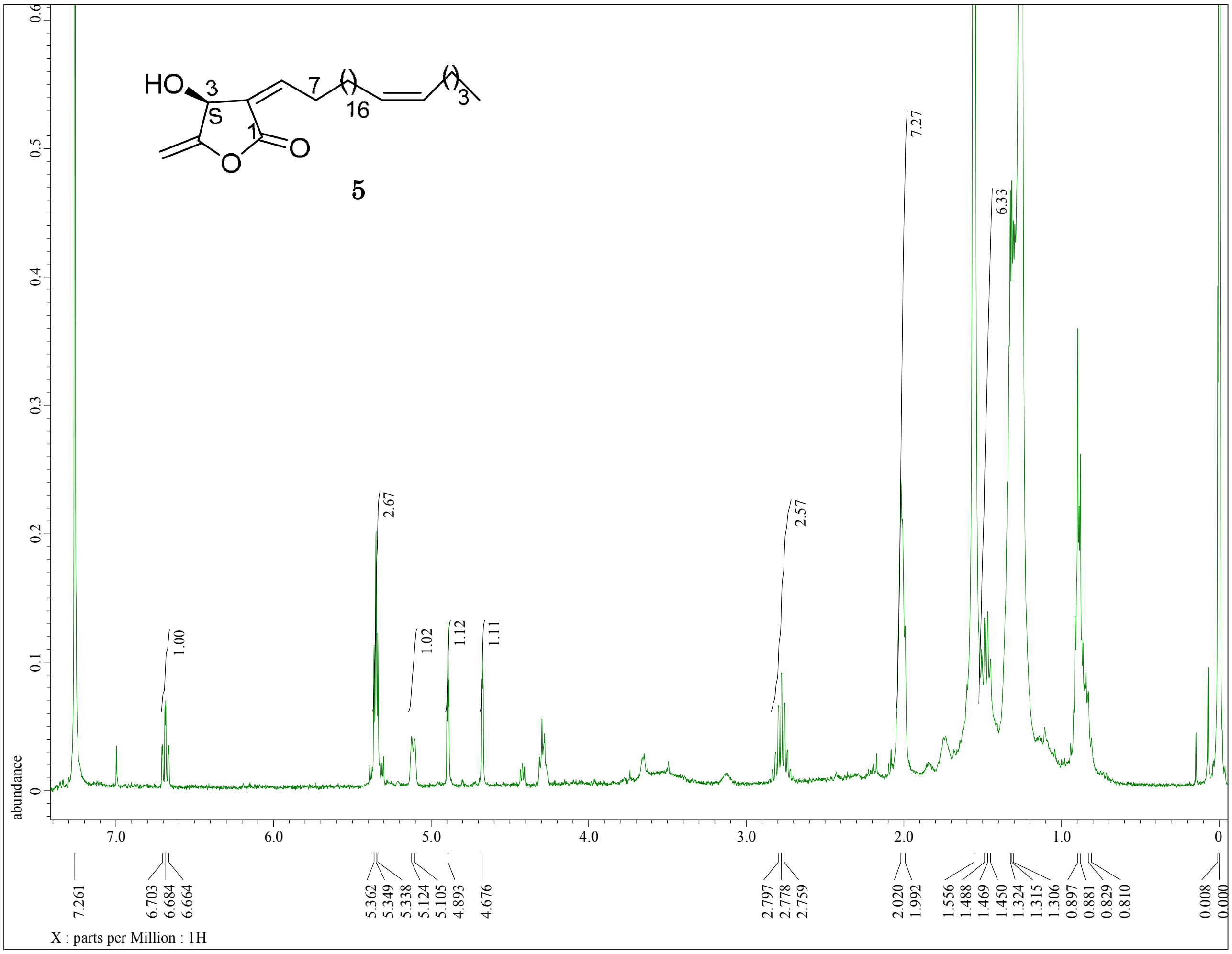

You created this PDF from an application that is not licensed to print to novaPDF printer (http://www.novapdf.com)

Figure S26. ^{13}C NMR spectrum of **5** (100MHz, in $CDCl_3$).

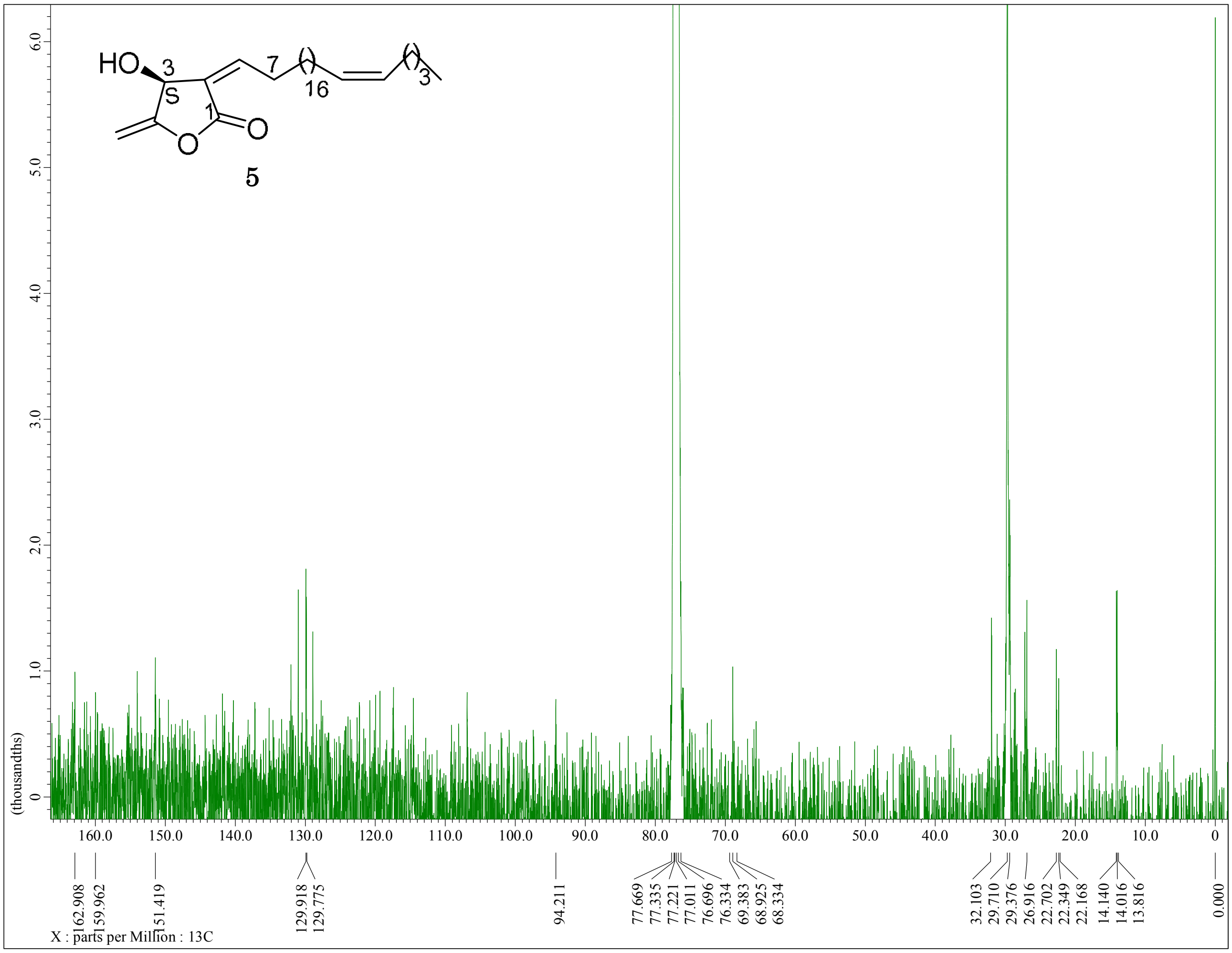

You created this PDF from an application that is not licensed to print to novaPDF printer (http://www.novapdf.com)

Figure S27. H-H COSY experiment of **5** (400MHz, in $CDCl_3$).

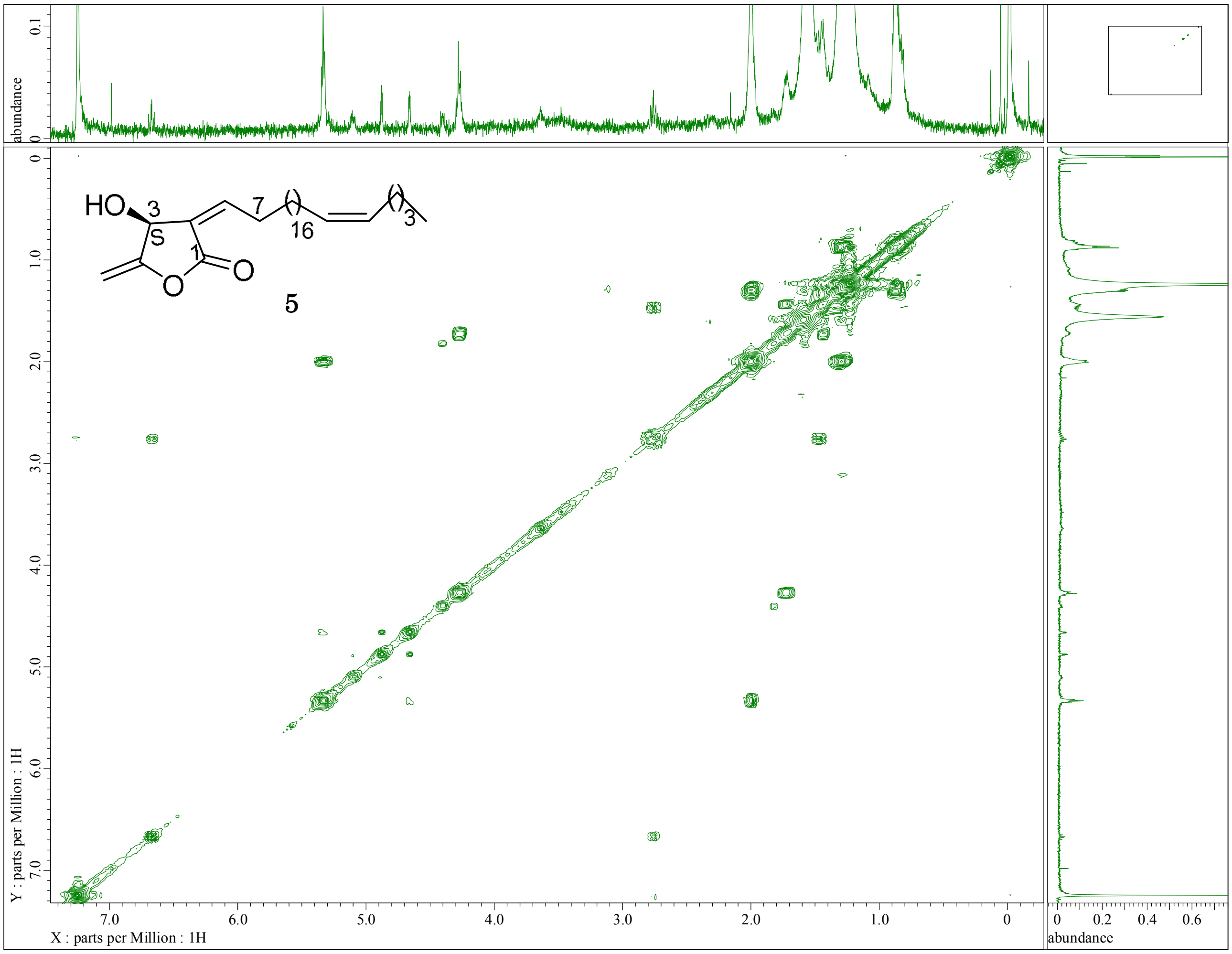

You created this PDF from an application that is not licensed to print to novaPDF printer (http://www.novapdf.com)

Figure S28. HMQC experiment of **5** (400MHz, in $CDCl_3$).

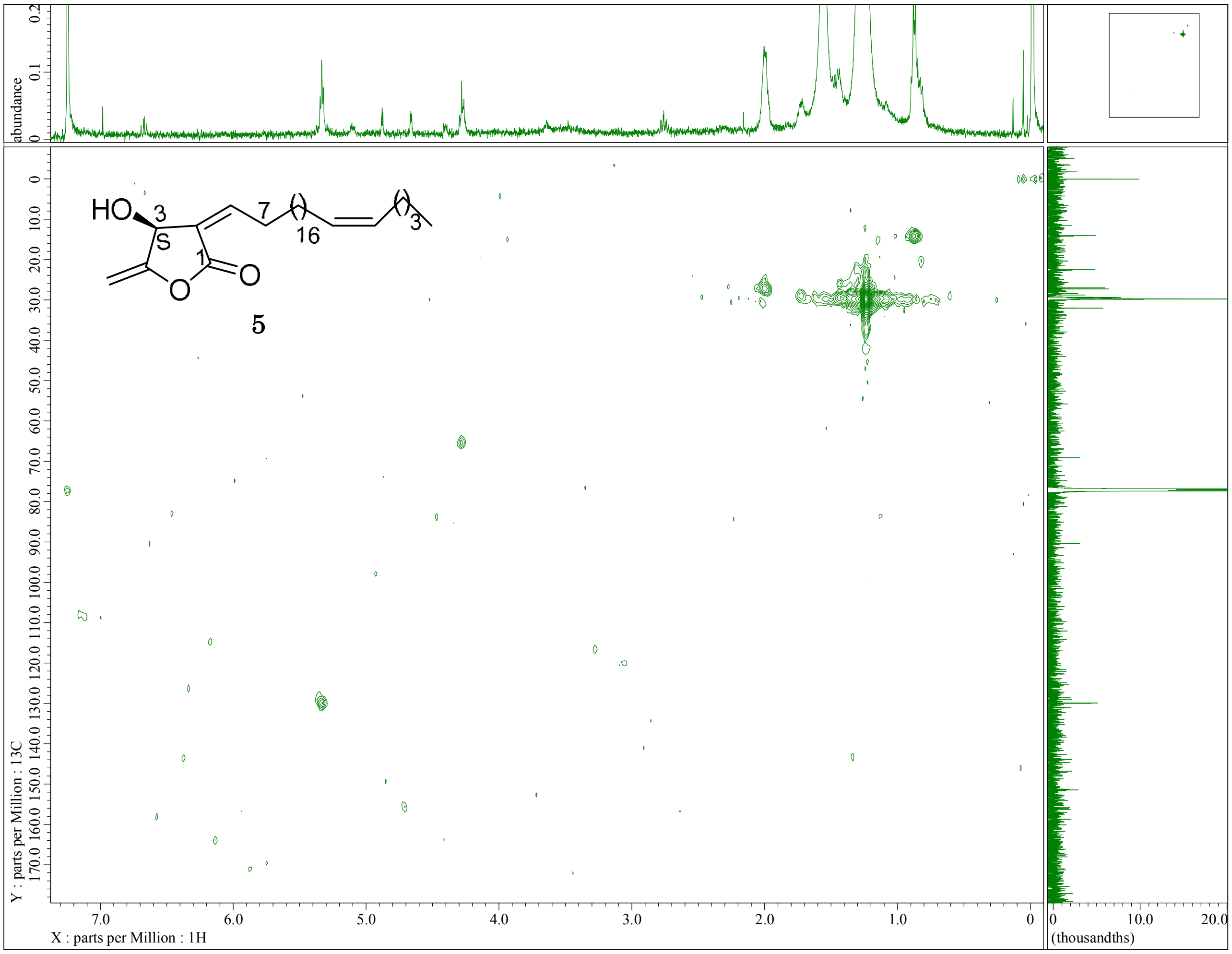

You created this PDF from an application that is not licensed to print to novaPDF printer (http://www.novapdf.com)

Figure S29. HMBC experiment of **5** (400MHz, in $CDCl_3$).

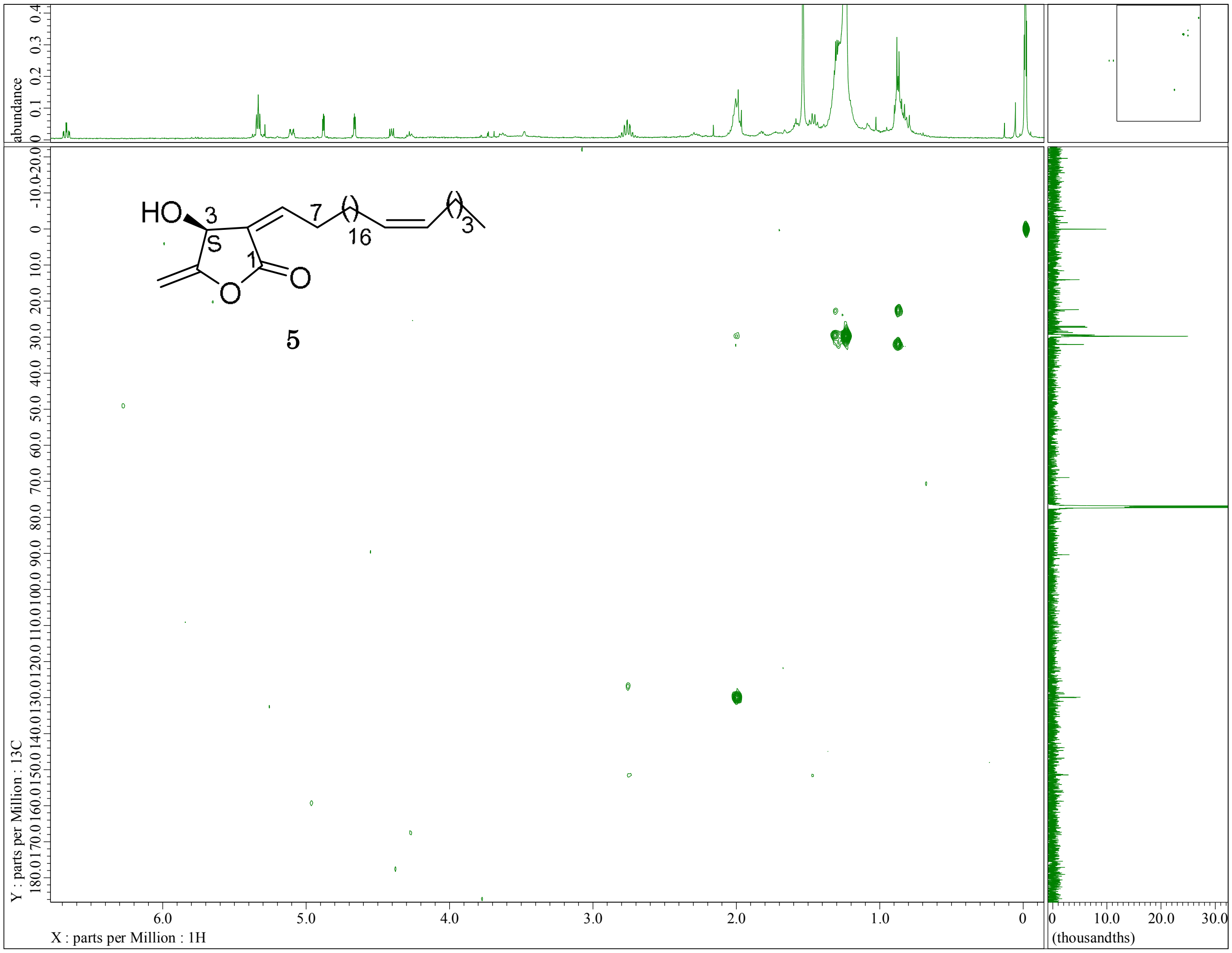

You created this PDF from an application that is not licensed to print to novaPDF printer (http://www.novapdf.com)

Figure S30. NOESY experiment of **5** (400MHz, in $CDCl_3$)

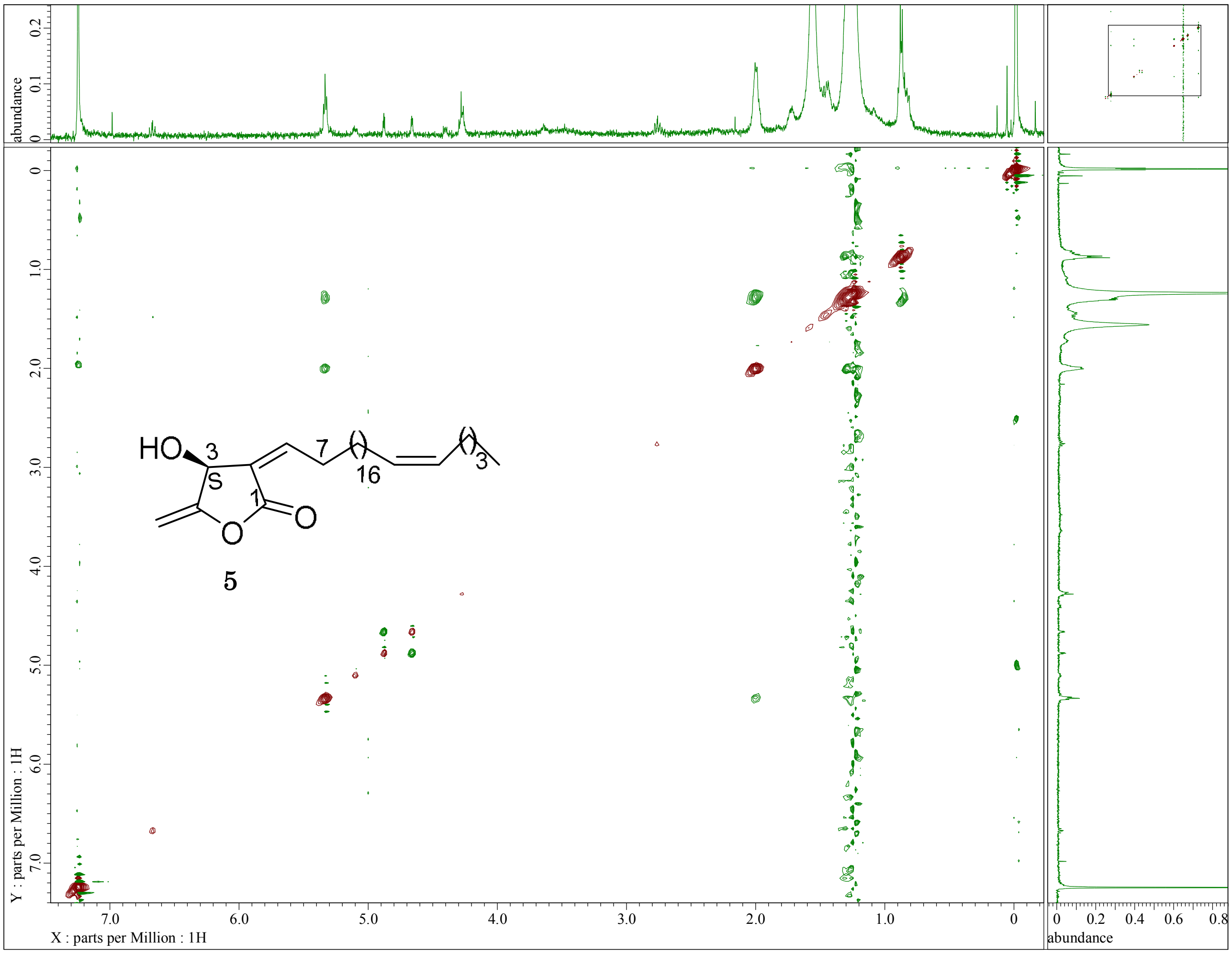

You created this PDF from an application that is not licensed to print to novaPDF printer (http://www.novapdf.com)

Figure S31. ^{1}H NMR spectrum of **6** (600MHz, in $CDCl_3$)

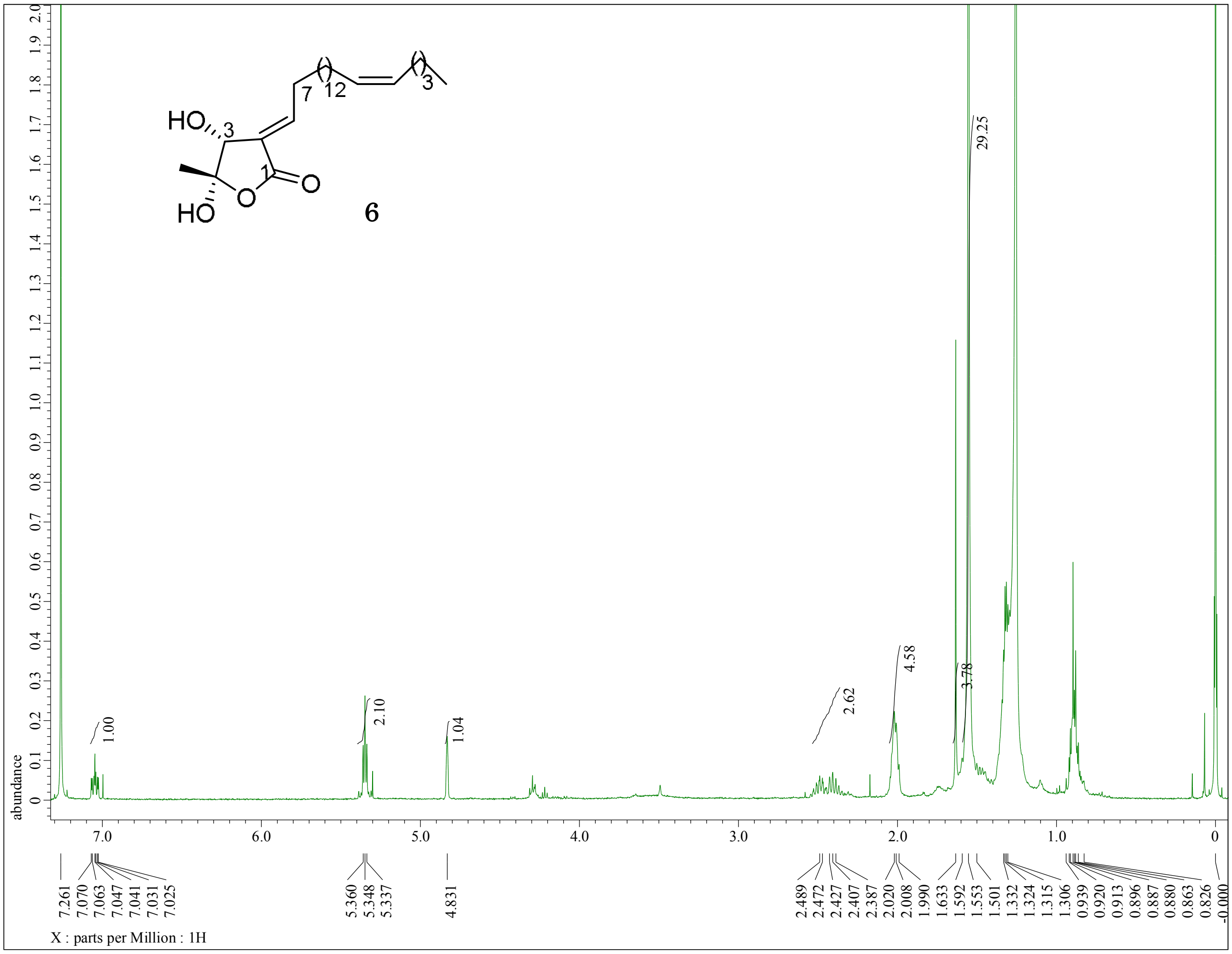

You created this PDF from an application that is not licensed to print to novaPDF printer (http://www.novapdf.com)

Figure S32. ^{13}C NMR spectrum of **6** (150MHz, in $CDCl_3$)

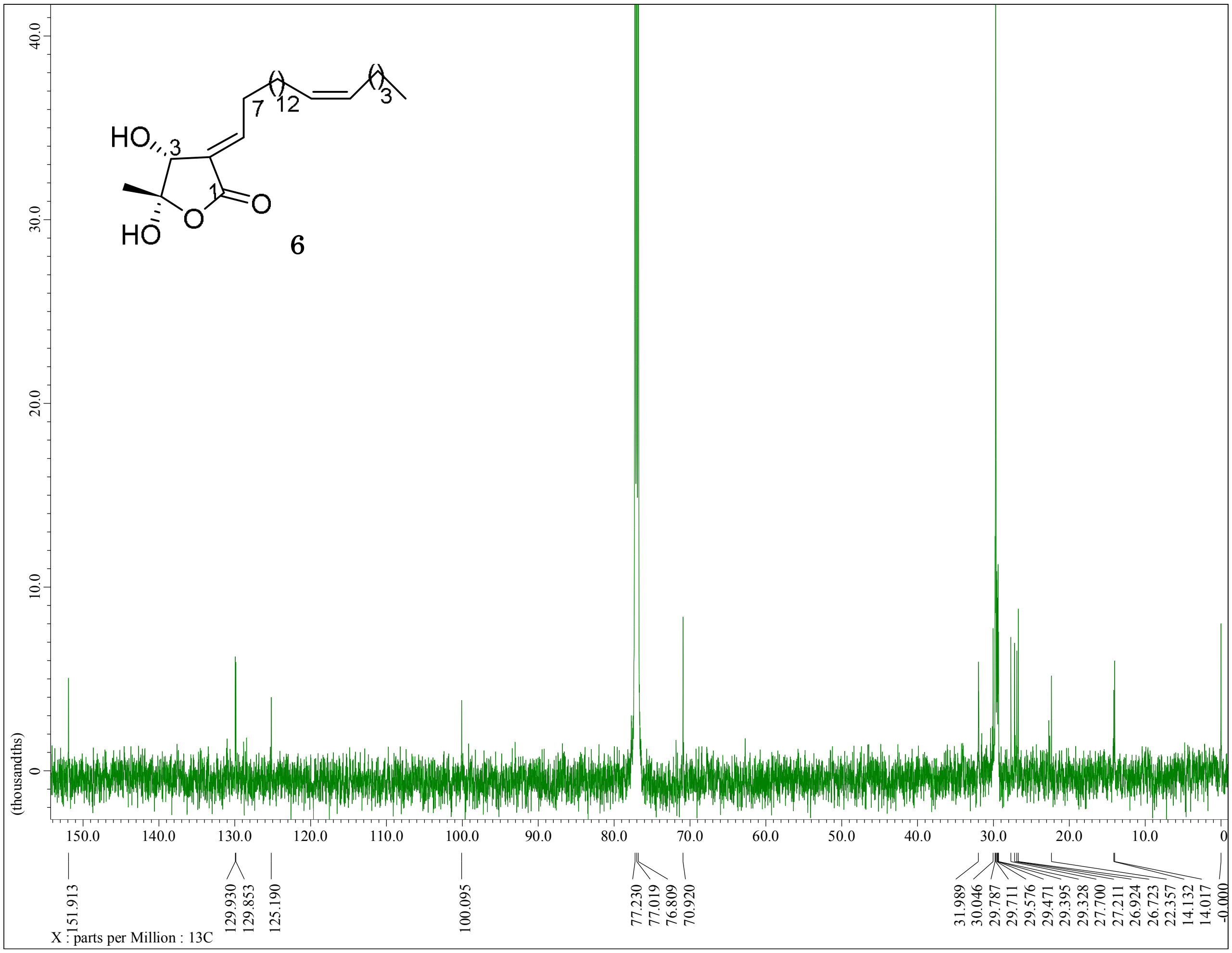

You created this PDF from an application that is not licensed to print to novaPDF printer (http://www.novapdf.com)

Figure S33. H-H COSY experiment of **6** (600MHz, in $CDCl_3$)

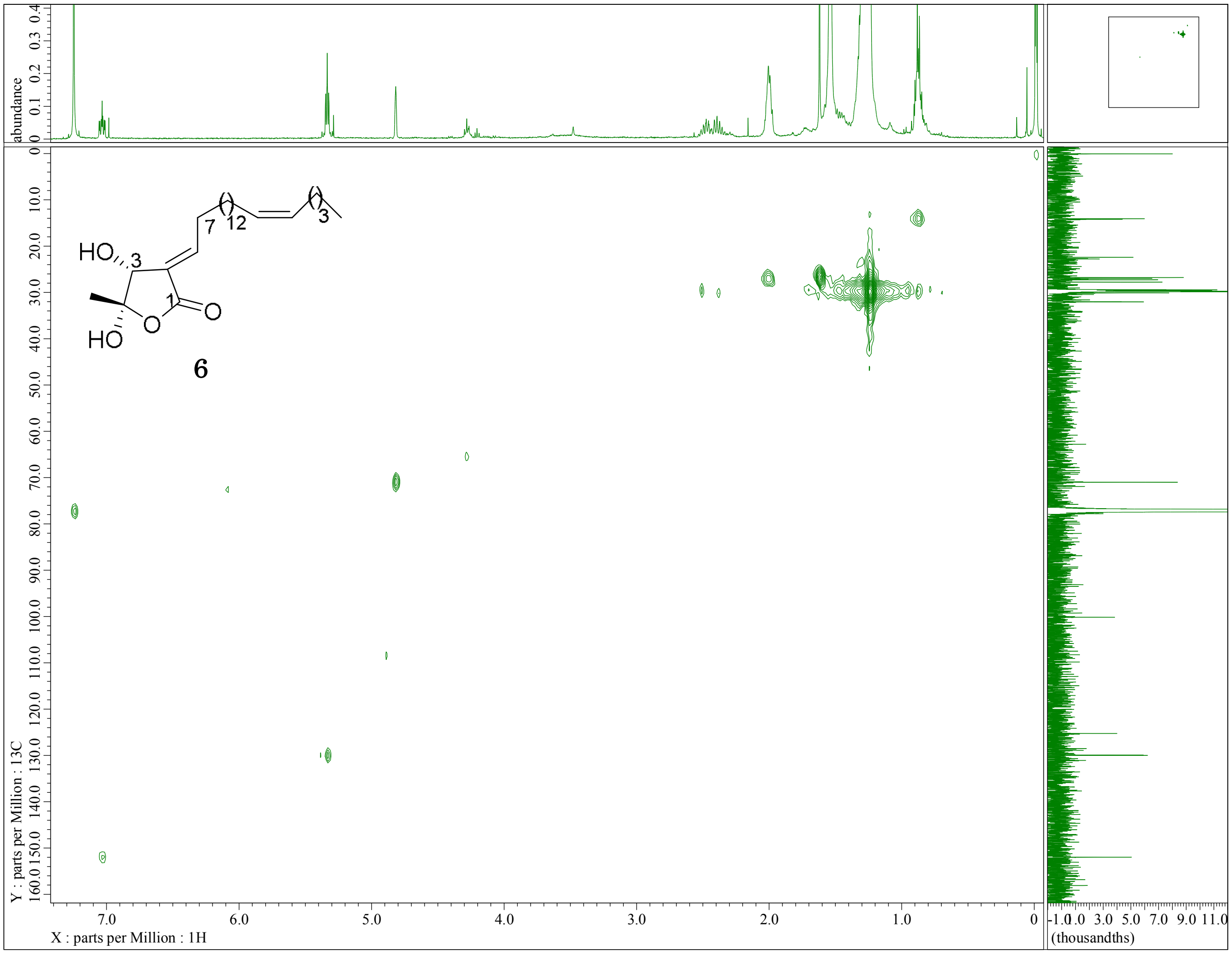

You created this PDF from an application that is not licensed to print to novaPDF printer (http://www.novapdf.com)

Figure S34. HMQC experiment of **6** (600MHz, in $CDCl_3$)

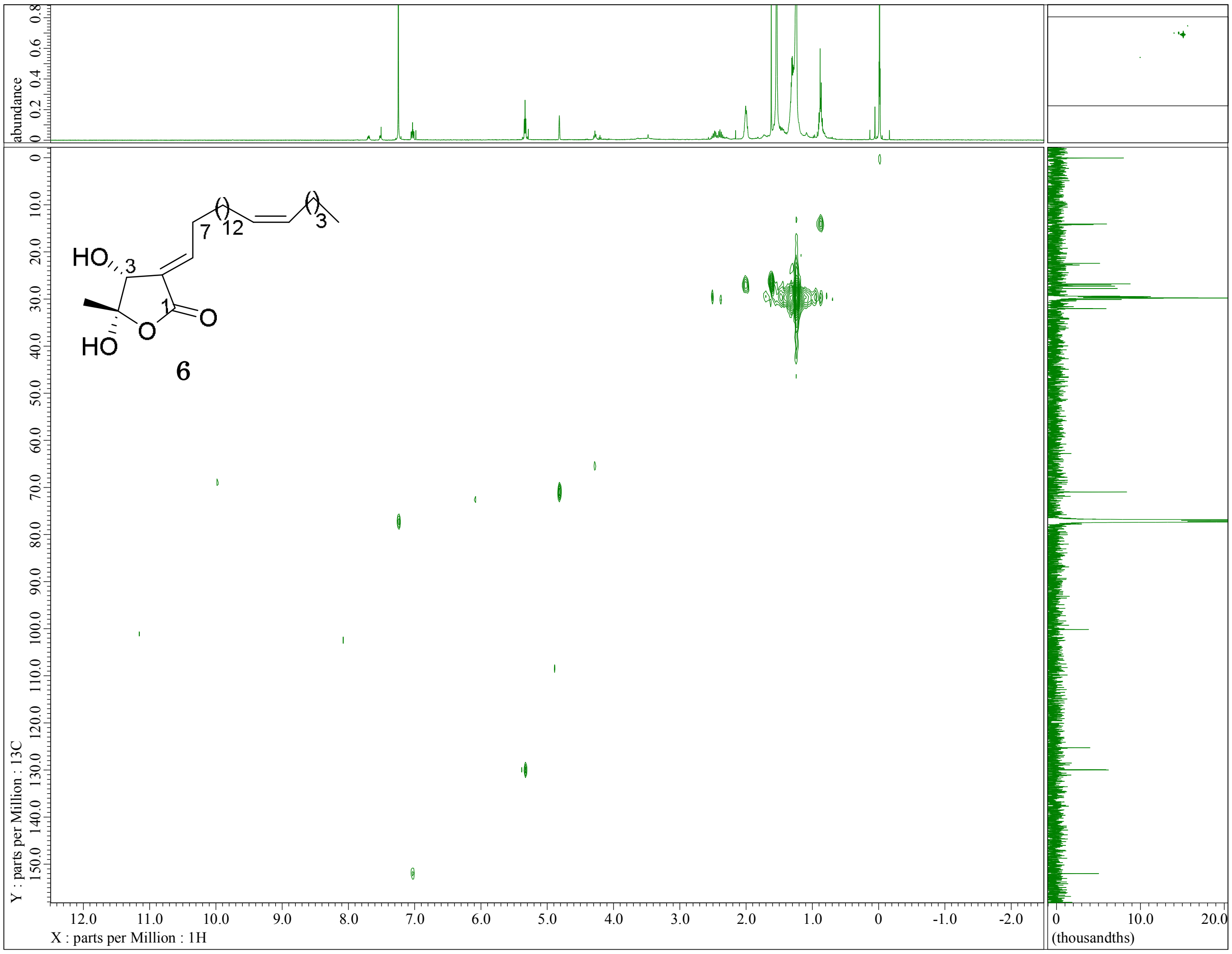

You created this PDF from an application that is not licensed to print to novaPDF printer (http://www.novapdf.com)

Figure S35. HMBC experiment of **6** (600MHz, in $CDCl_3$)

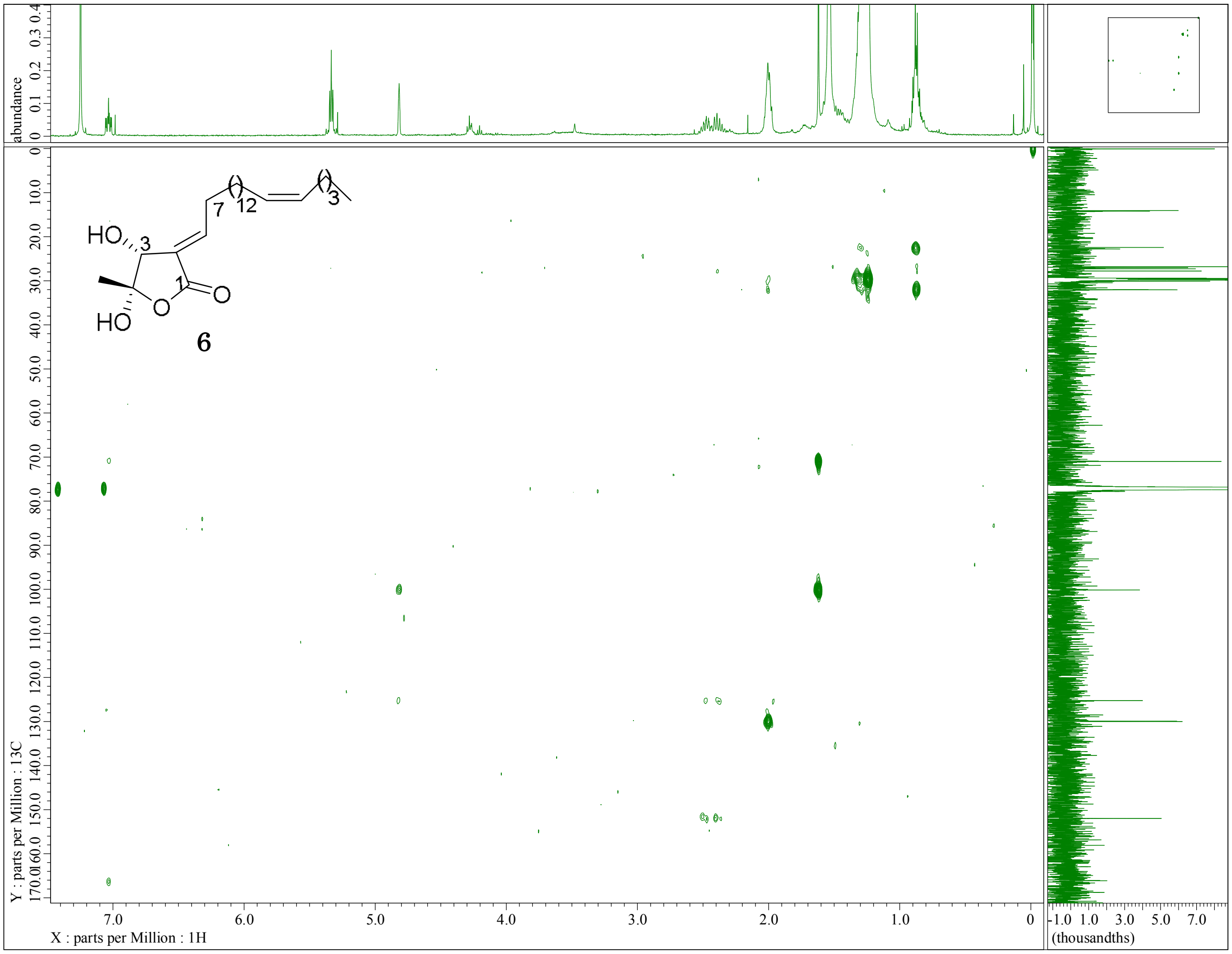

You created this PDF from an application that is not licensed to print to novaPDF printer (http://www.novapdf.com)

Figure S36. NOESY experiment of **6** (600MHz, in $CDCl_3$).

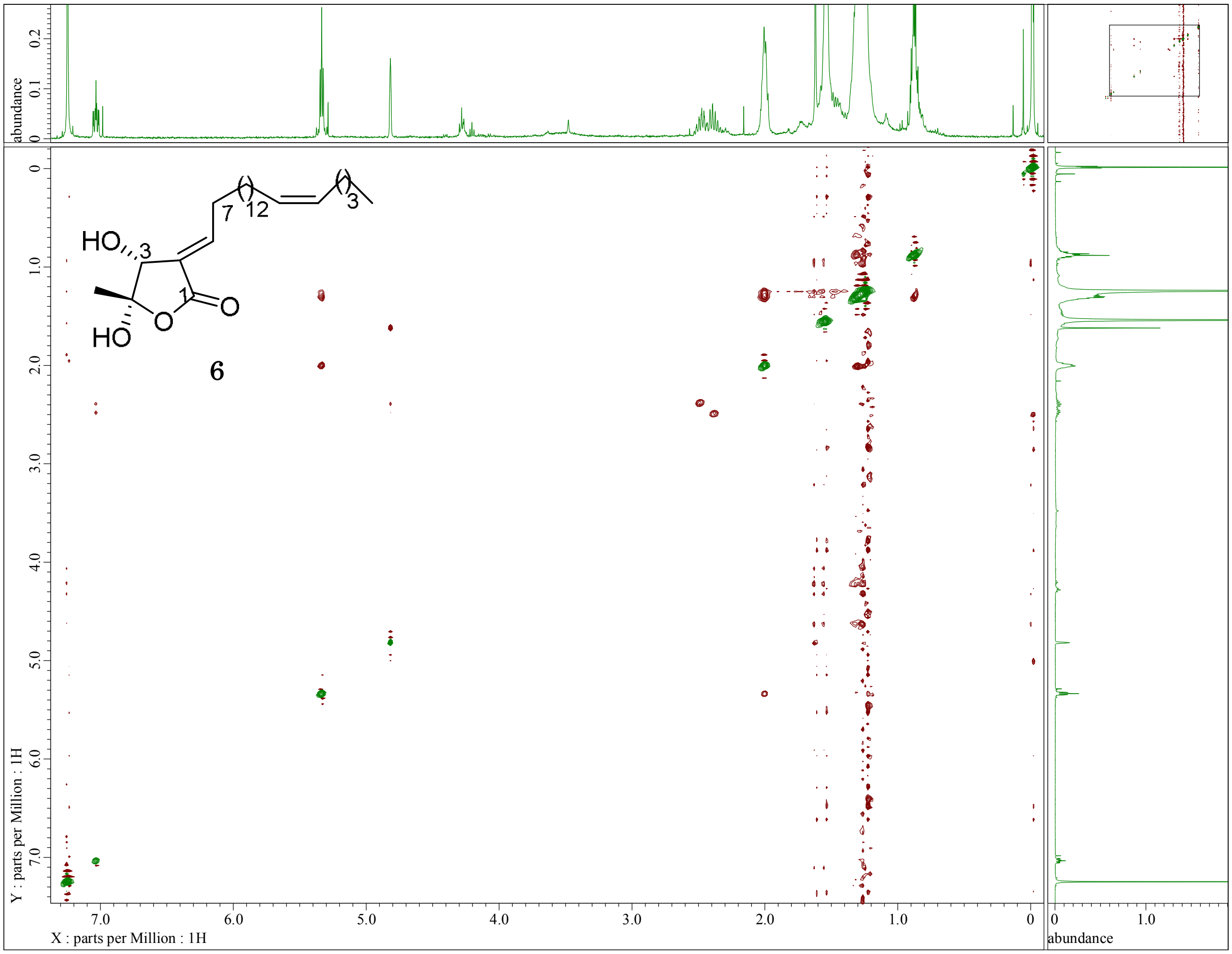

You created this PDF from an application that is not licensed to print to novaPDF printer (http://www.novapdf.com)

Figure S37. ^{1}H NMR spectrum of **7** (400MHz, in $CDCl_3$).

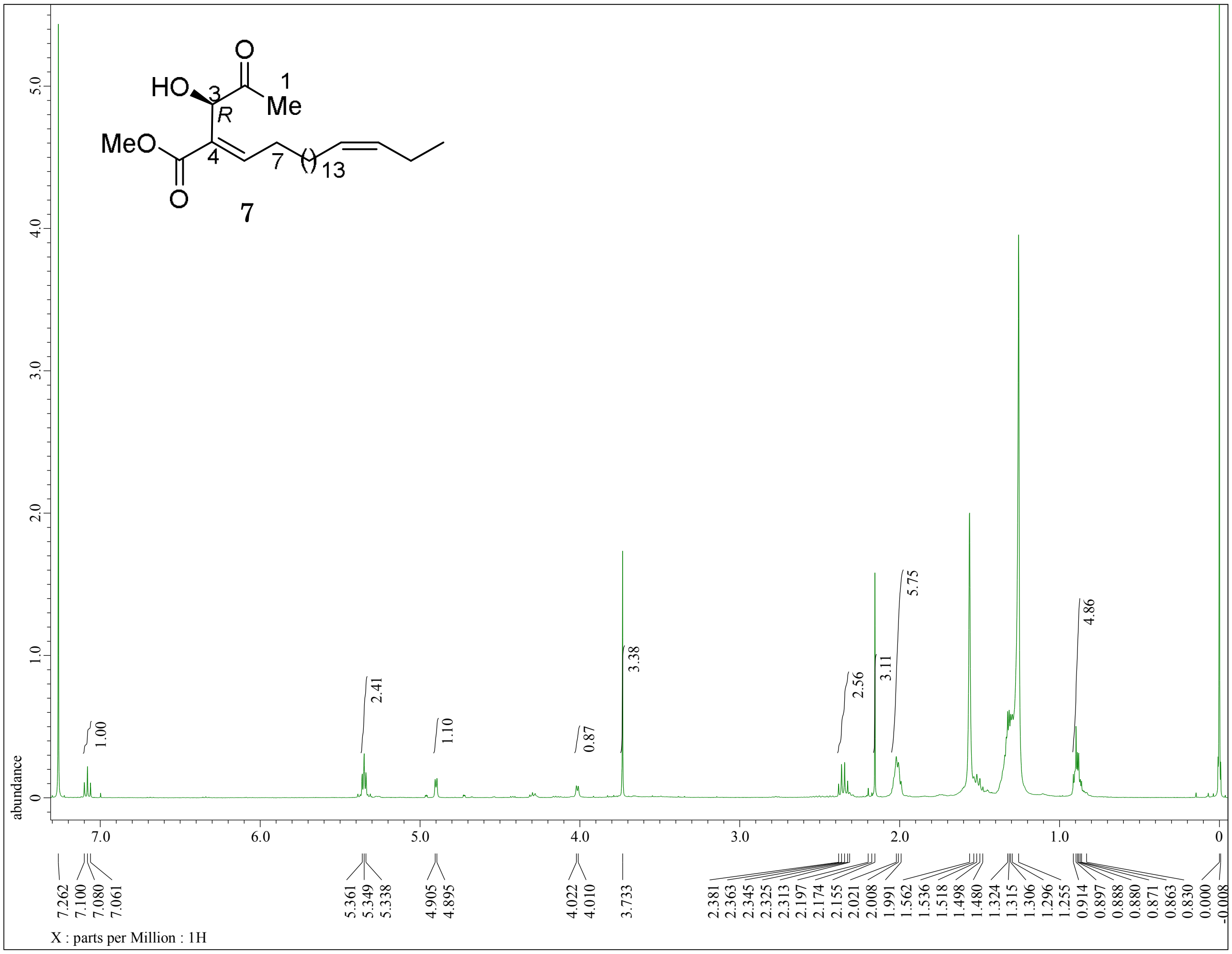

37

You created this PDF from an application that is not licensed to print to novaPDF printer (http://www.novapdf.com)

Figure S38. ^{13}C NMR spectrum of **7** (100MHz, in $CDCl_3$).

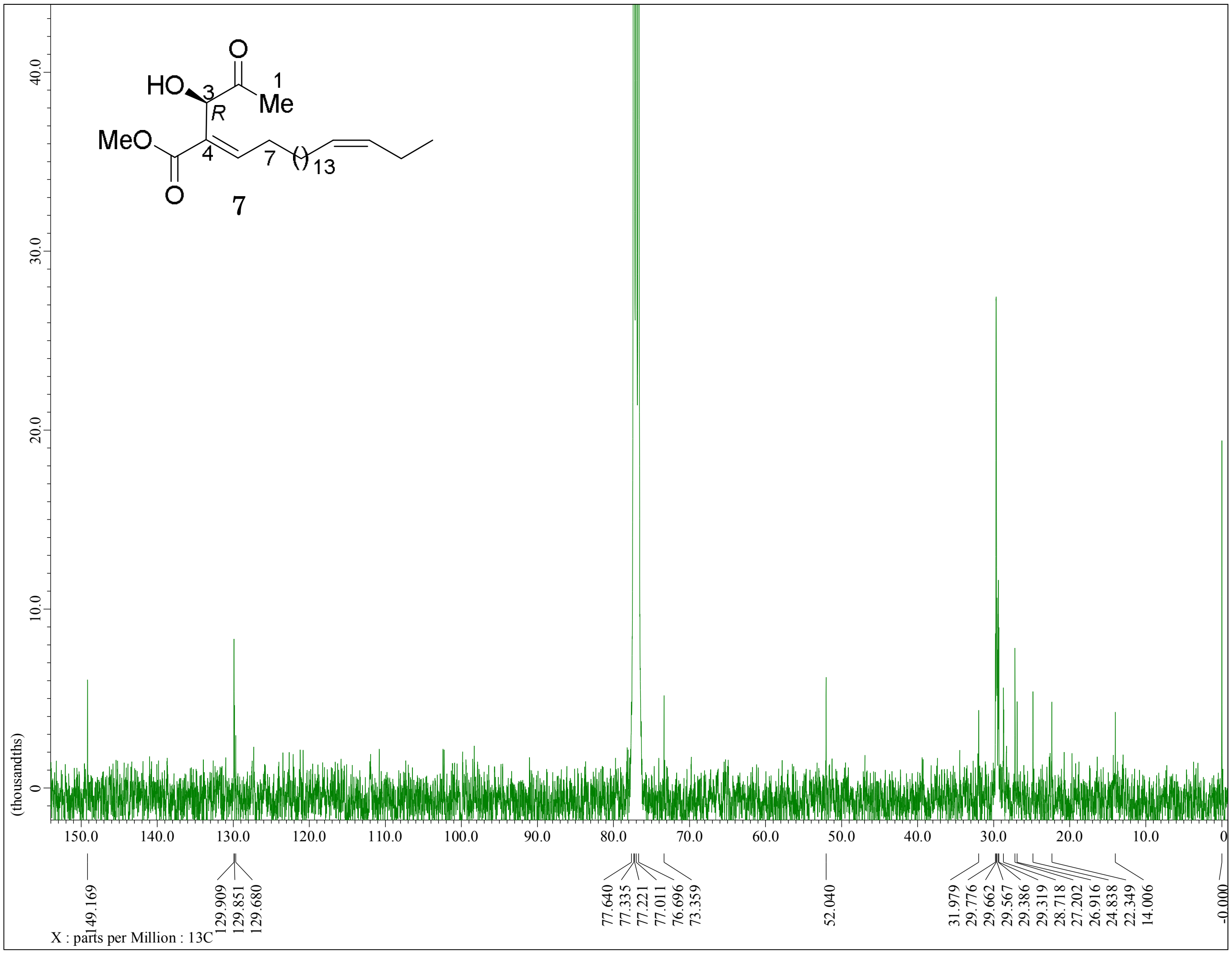

You created this PDF from an application that is not licensed to print to novaPDF printer (http://www.novapdf.com)

Figure S39. H-H COSY experiment of **7** (400MHz, in $CDCl_3$).

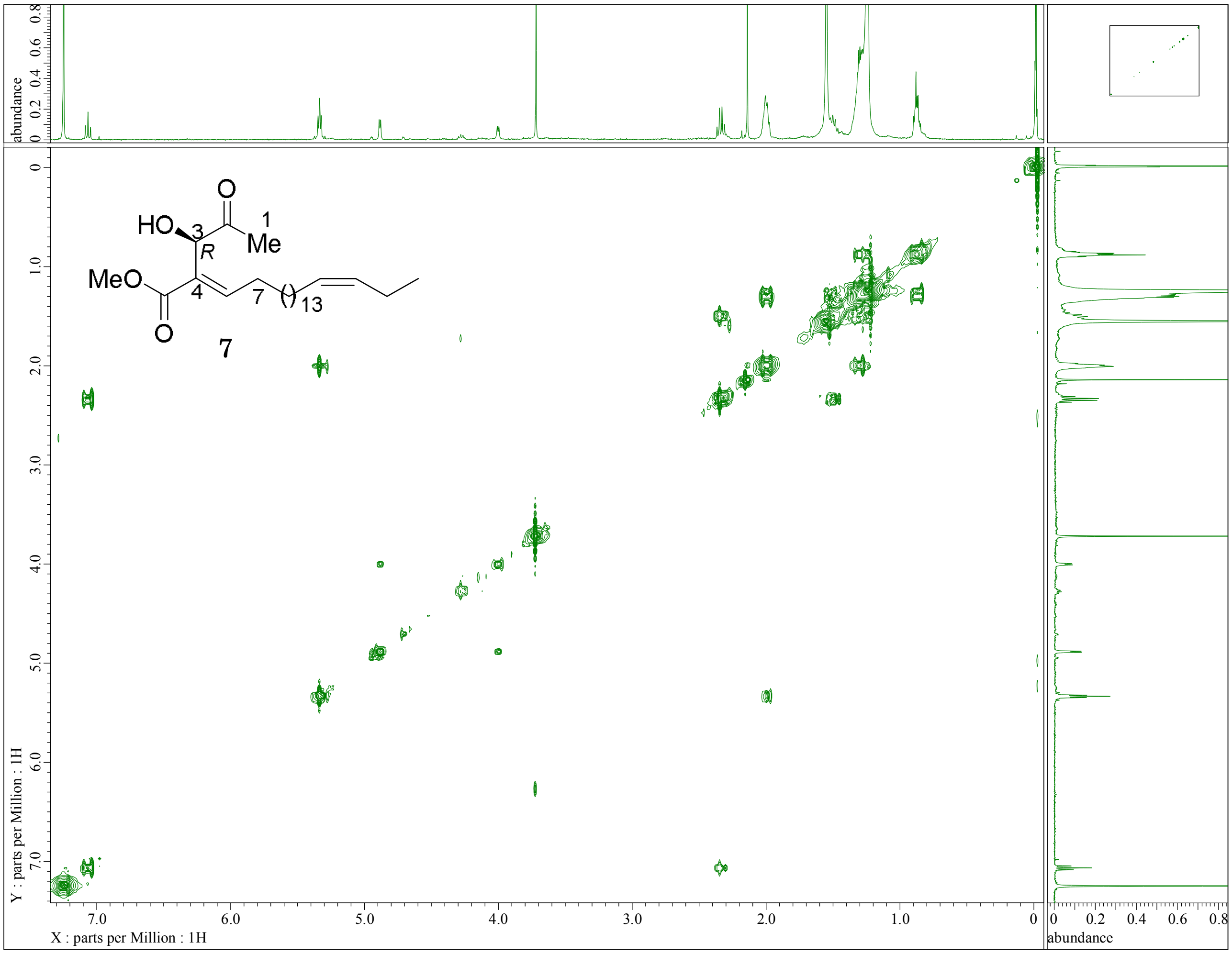

You created this PDF from an application that is not licensed to print to novaPDF printer (http://www.novapdf.com)

Figure S40. HMQC experiment of **7** (400MHz, in $CDCl_3$).

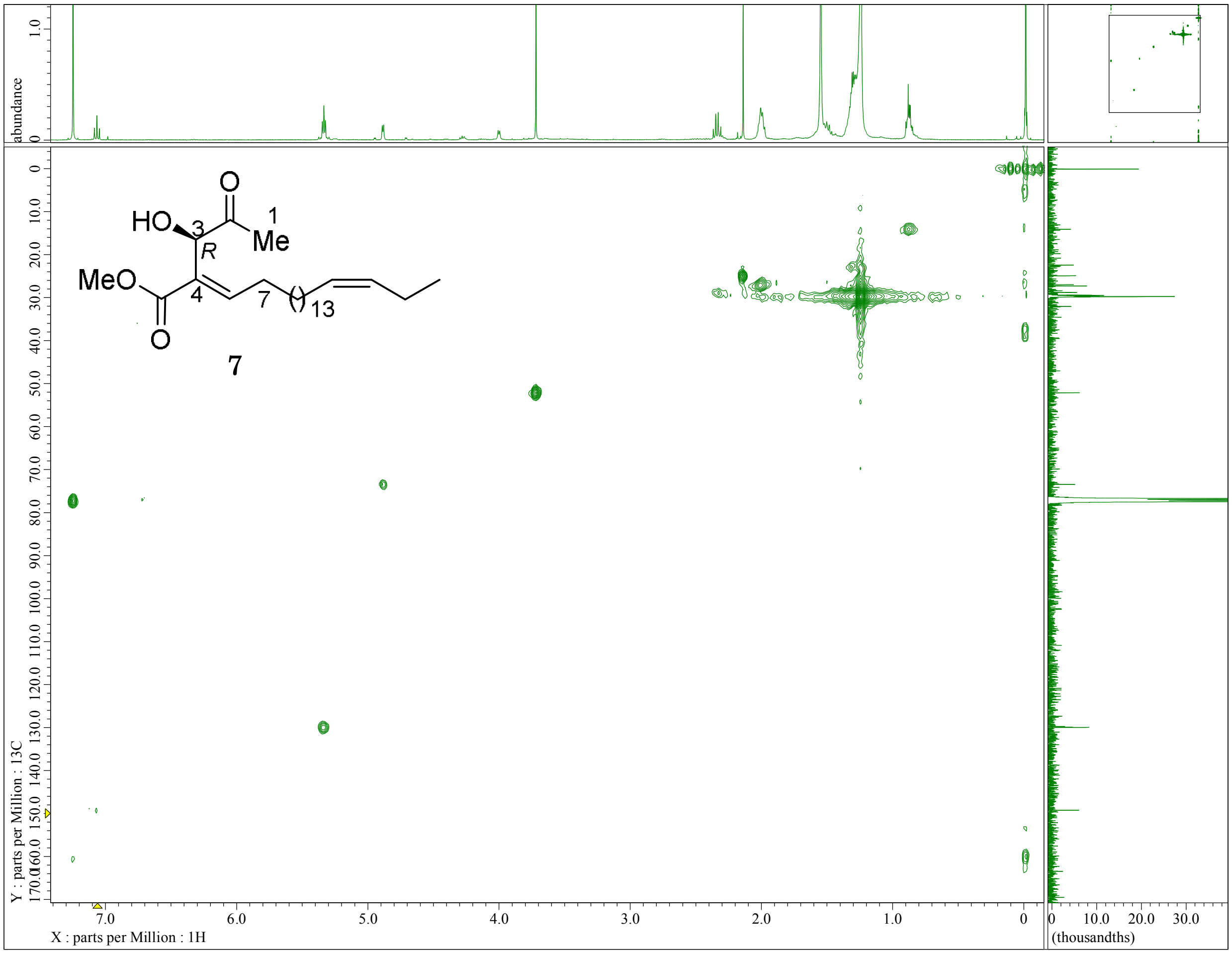

You created this PDF from an application that is not licensed to print to novaPDF printer (http://www.novapdf.com)

Figure S41. HMBC experiment of **7** (400MHz, in $CDCl_3$).

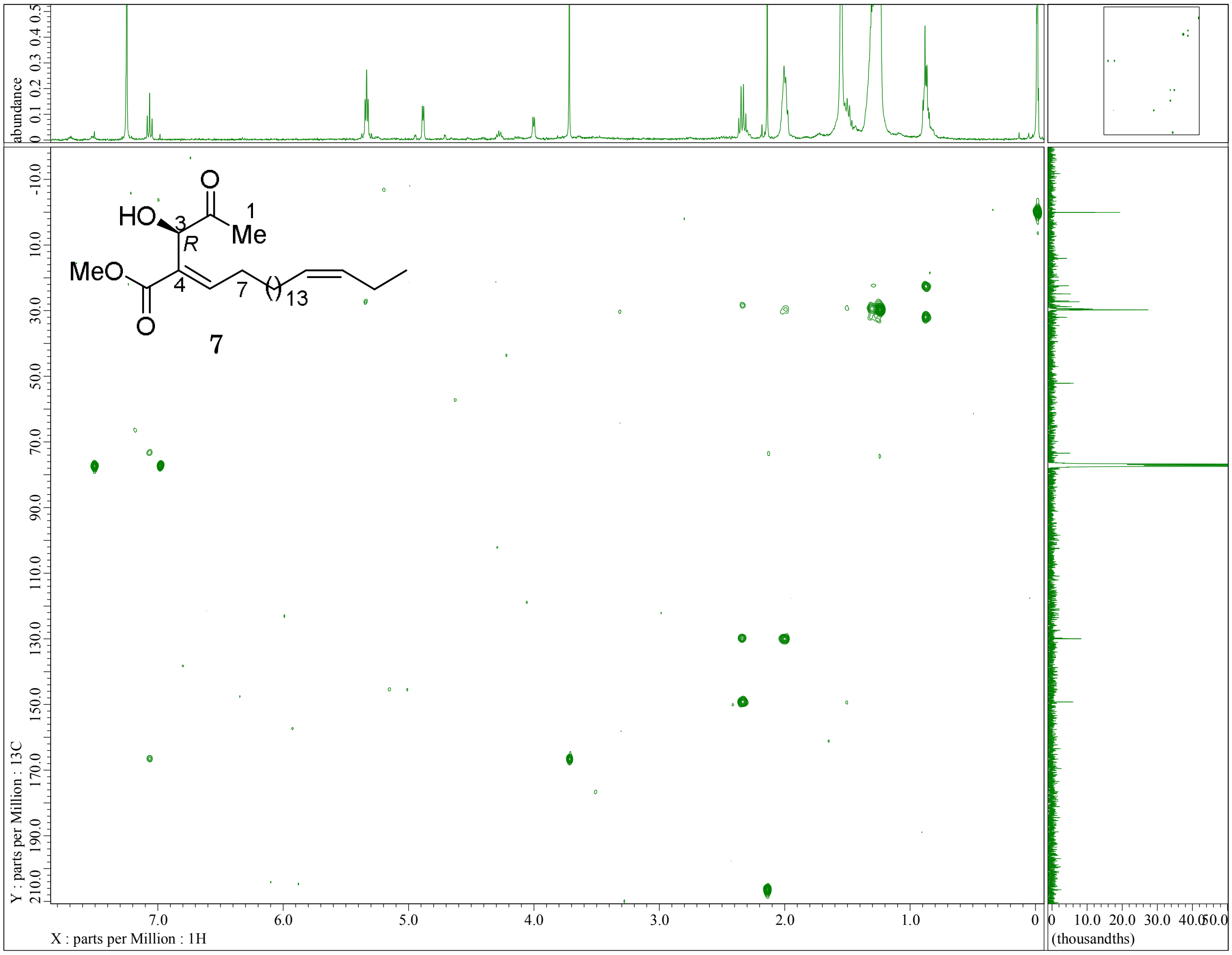

You created this PDF from an application that is not licensed to print to novaPDF printer (http://www.novapdf.com)

Figure S42. NOESY experiment of **7** (400MHz, in $CDCl_3$).

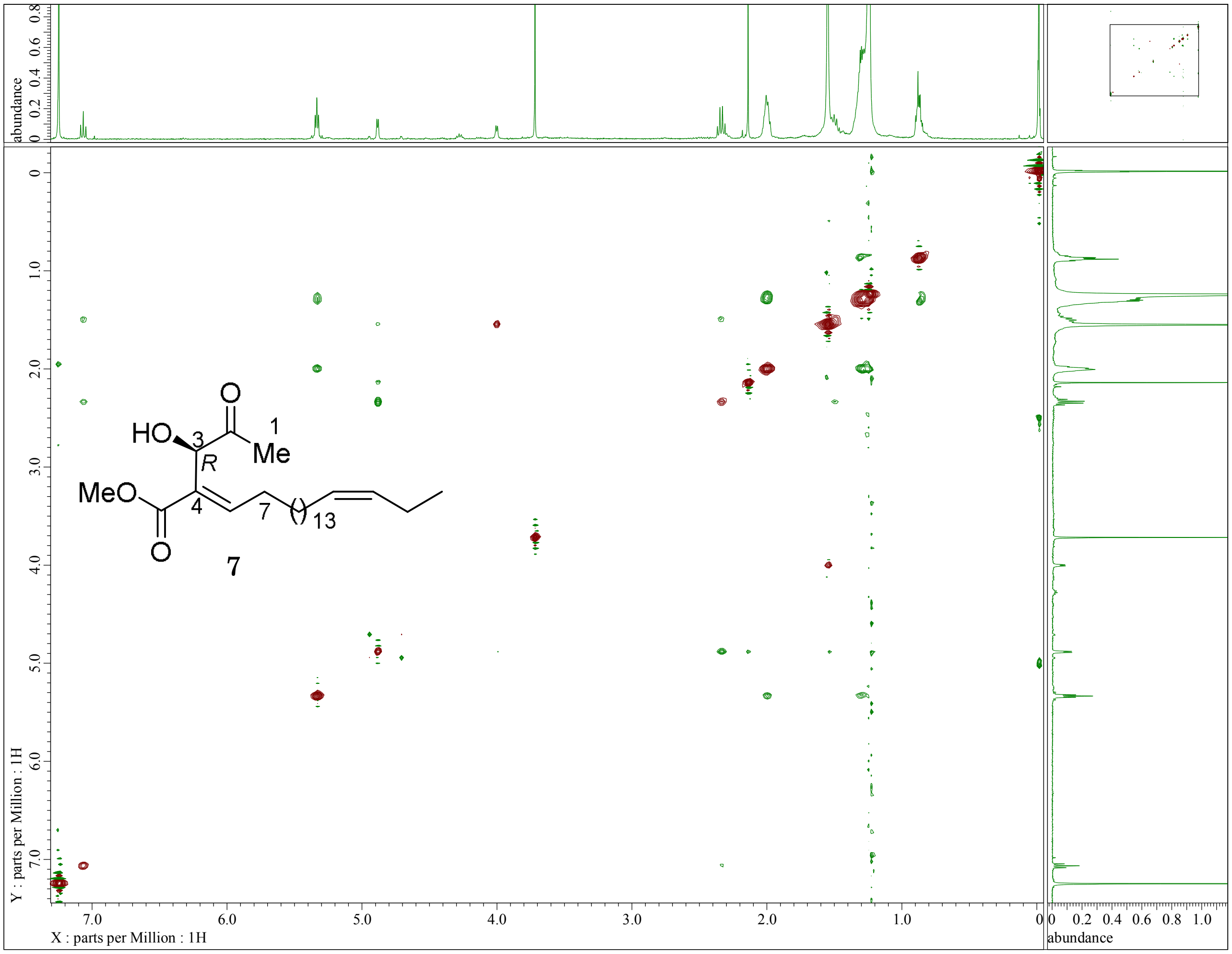

You created this PDF from an application that is not licensed to print to novaPDF printer (http://www.novapdf.com)

Figure S43. EIMS spectrum for **7**

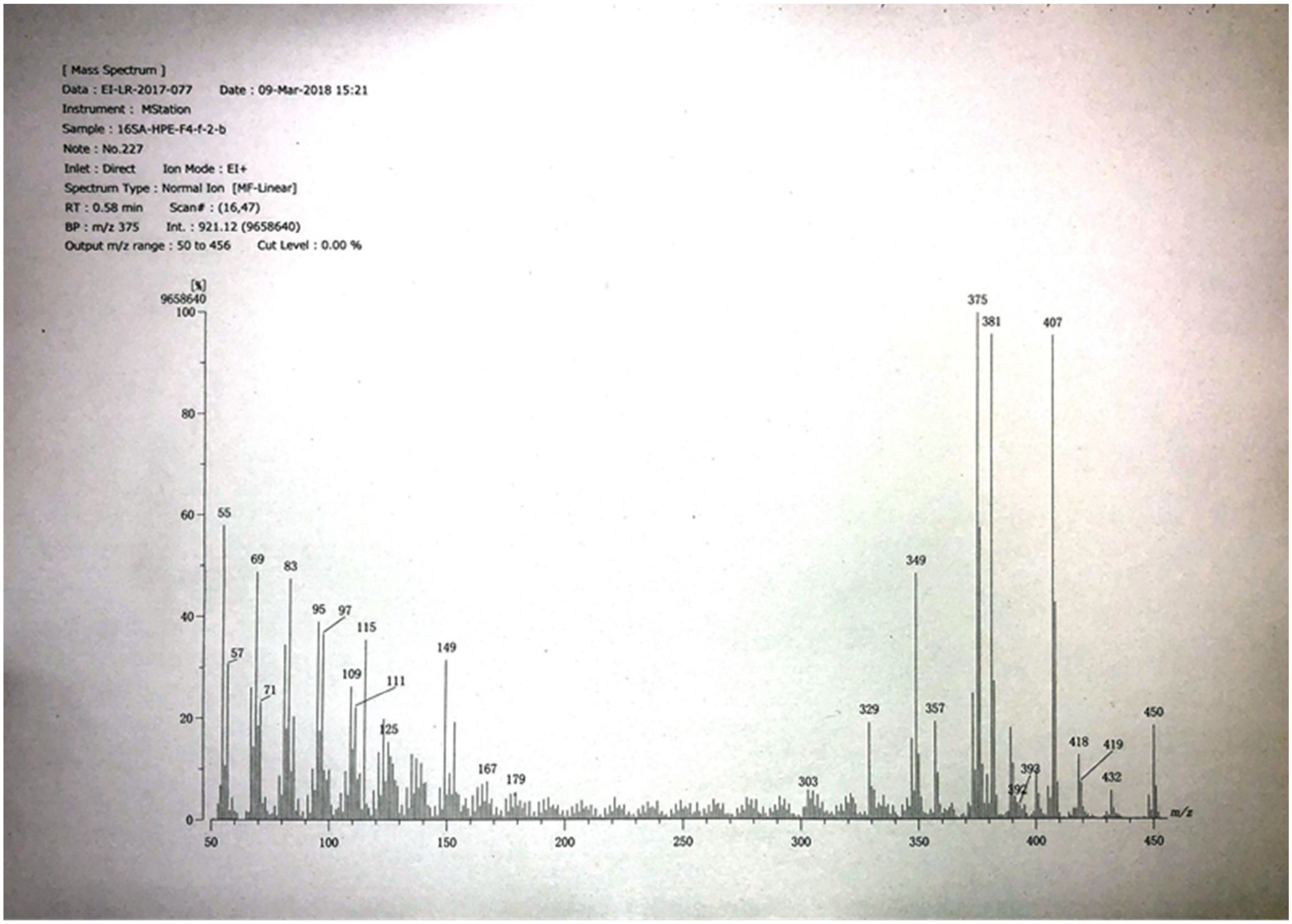

You created this PDF from an application that is not licensed to print to novaPDF printer (http://www.novapdf.com)

Figure S44. ^{1}H MNR spectrum of **8** (400MHz, in CD_3OD)

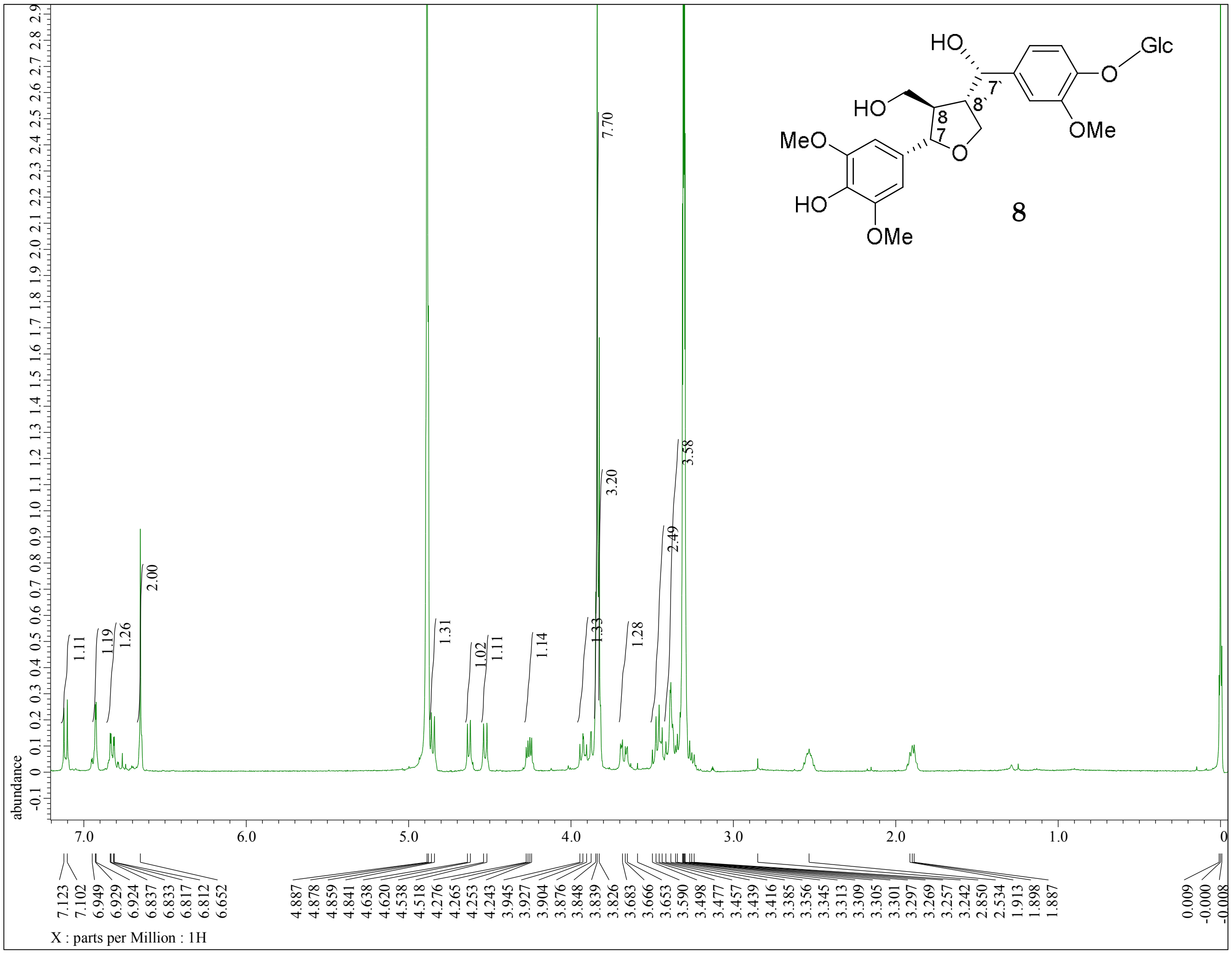

You created this PDF from an application that is not licensed to print to novaPDF printer (http://www.novapdf.com)

Figure S45. ^{13}C MNR spectrum of **8** (100MHz, in CD_3OD)

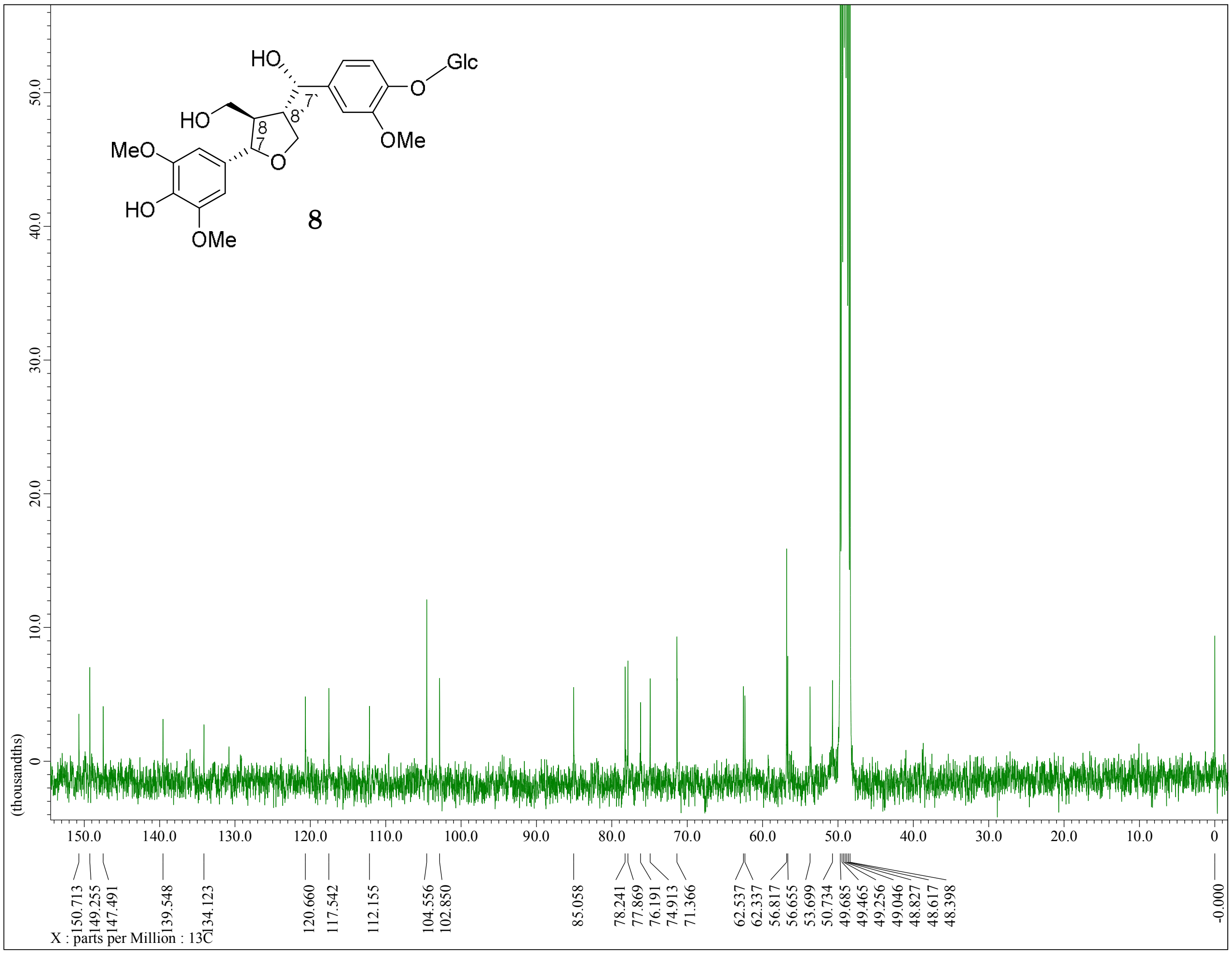

You created this PDF from an application that is not licensed to print to novaPDF printer (http://www.novapdf.com)

Figure S46. H-H COSY experiment of **8** (400MHz, in CD_3OD)

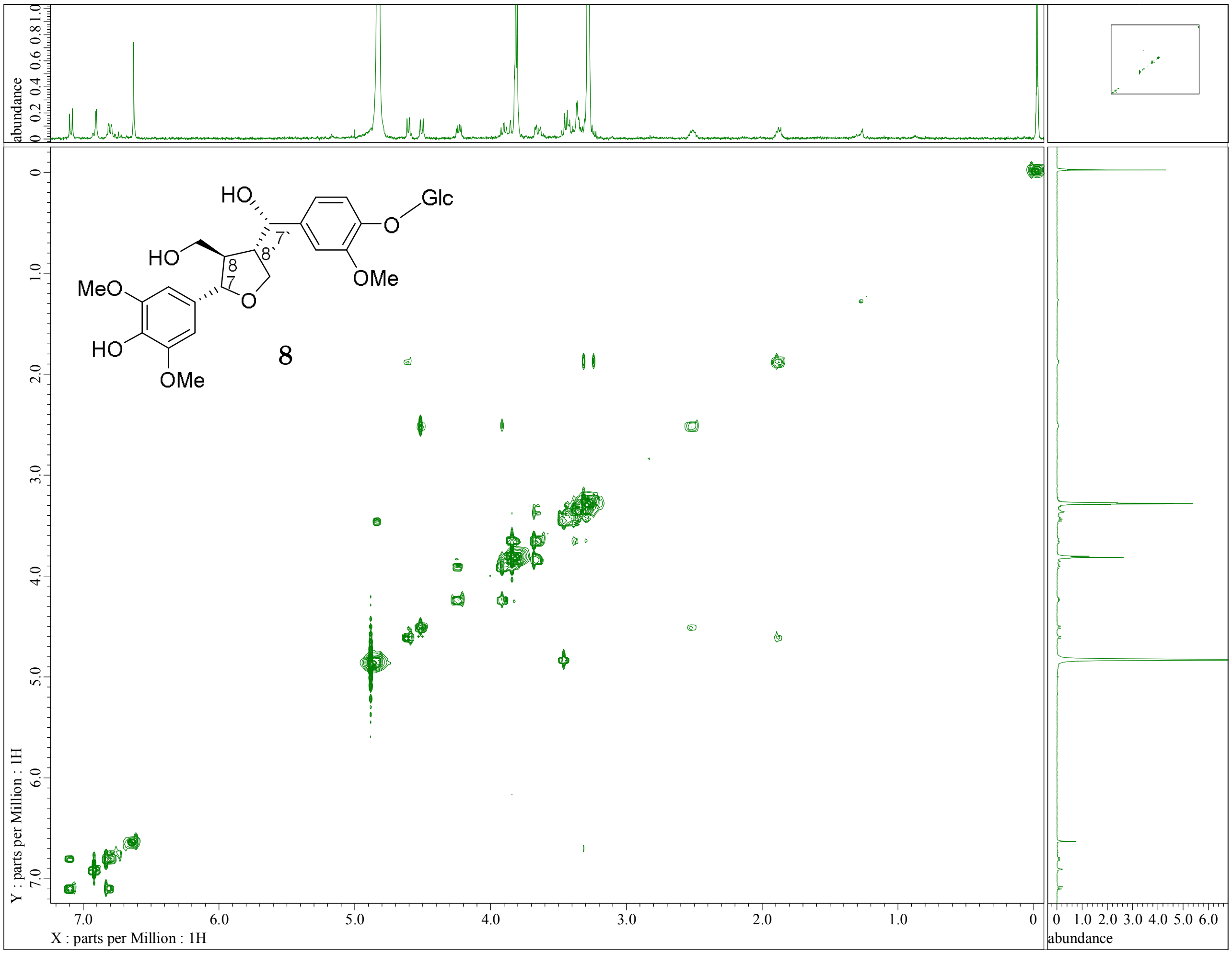

You created this PDF from an application that is not licensed to print to novaPDF printer (http://www.novapdf.com)

Figure S47. HMQC experiment of **8** (400MHz, in CD_3OD)

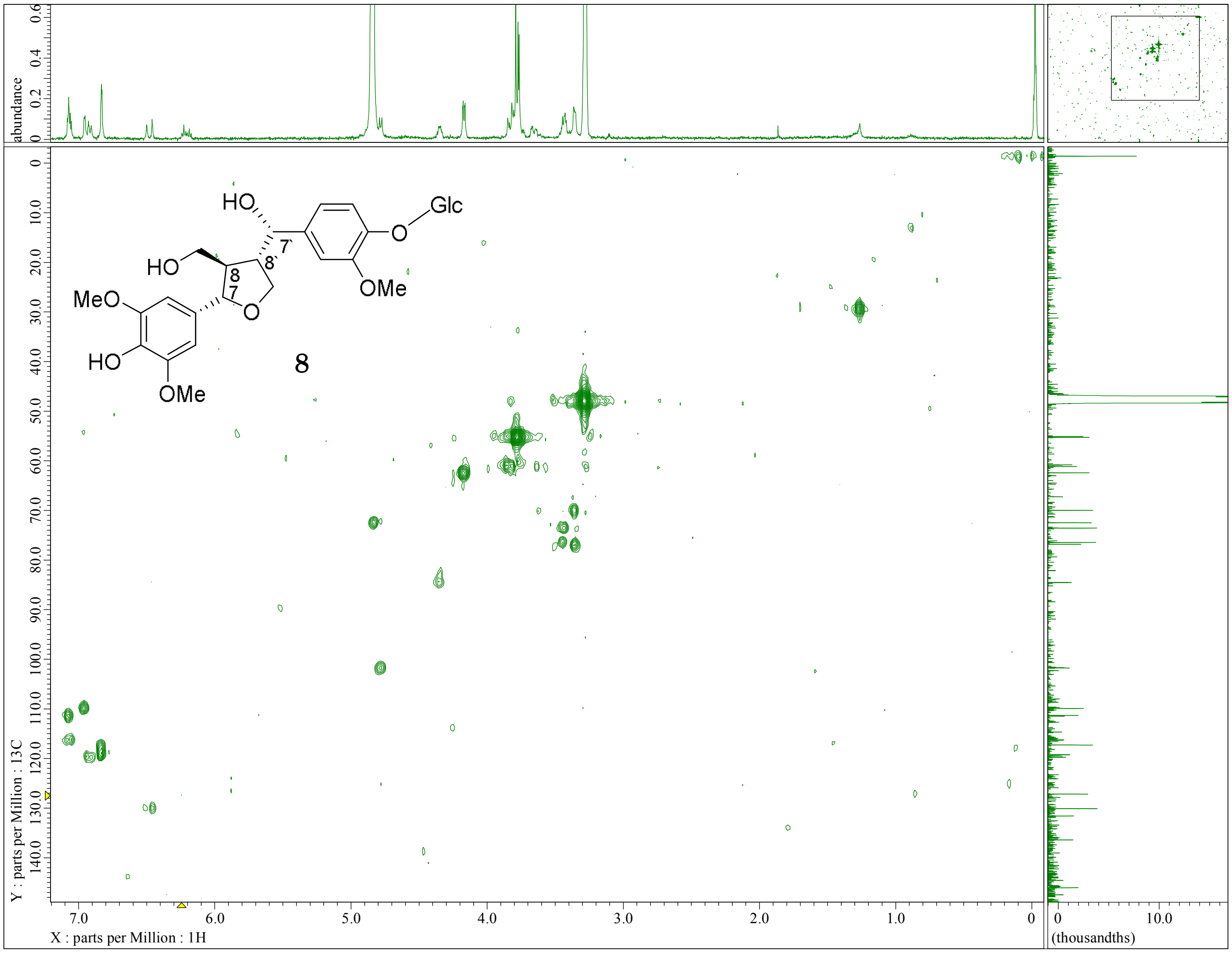

You created this PDF from an application that is not licensed to print to novaPDF printer (http://www.novapdf.com)

Figure S48. HMBC experiment of **8** (400MHz, in CD_3OD)

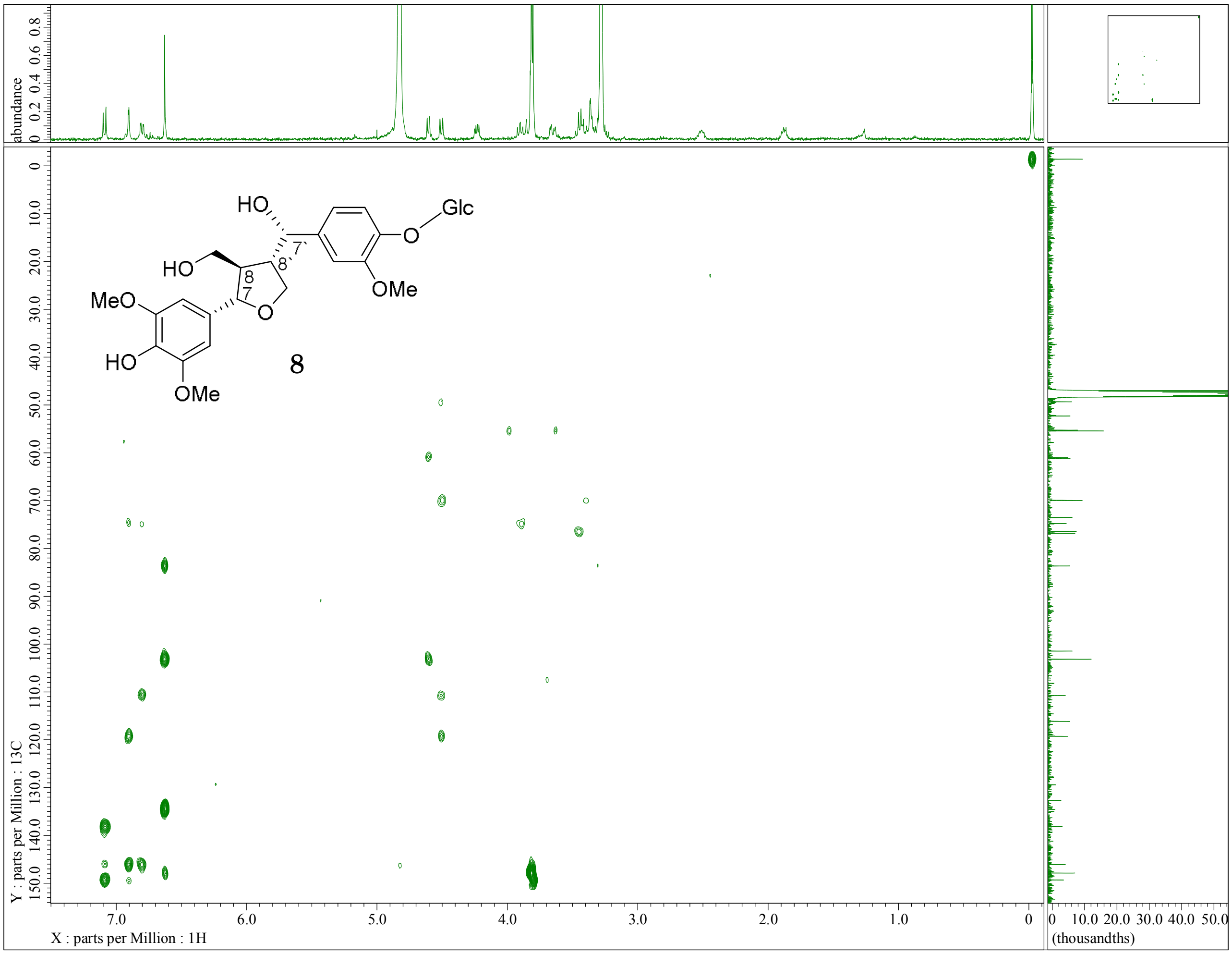

You created this PDF from an application that is not licensed to print to novaPDF printer (http://www.novapdf.com)

Figure S49. NOESY experiment of **8** (400MHz, in CD_3OD)

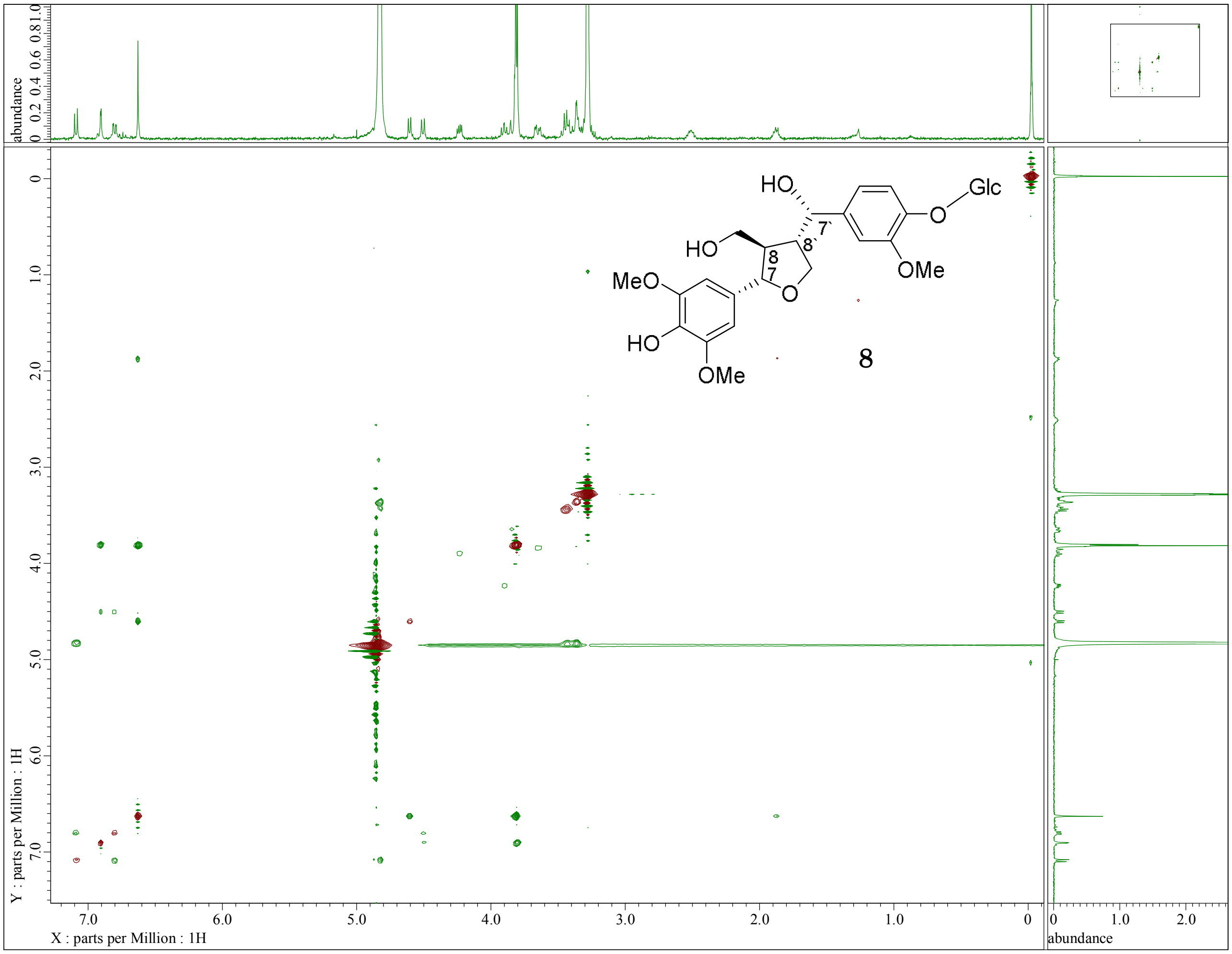

You created this PDF from an application that is not licensed to print to novaPDF printer (http://www.novapdf.com)

Figure S50. ROESY experiment of **8** (400MHz, in CD_3OD)

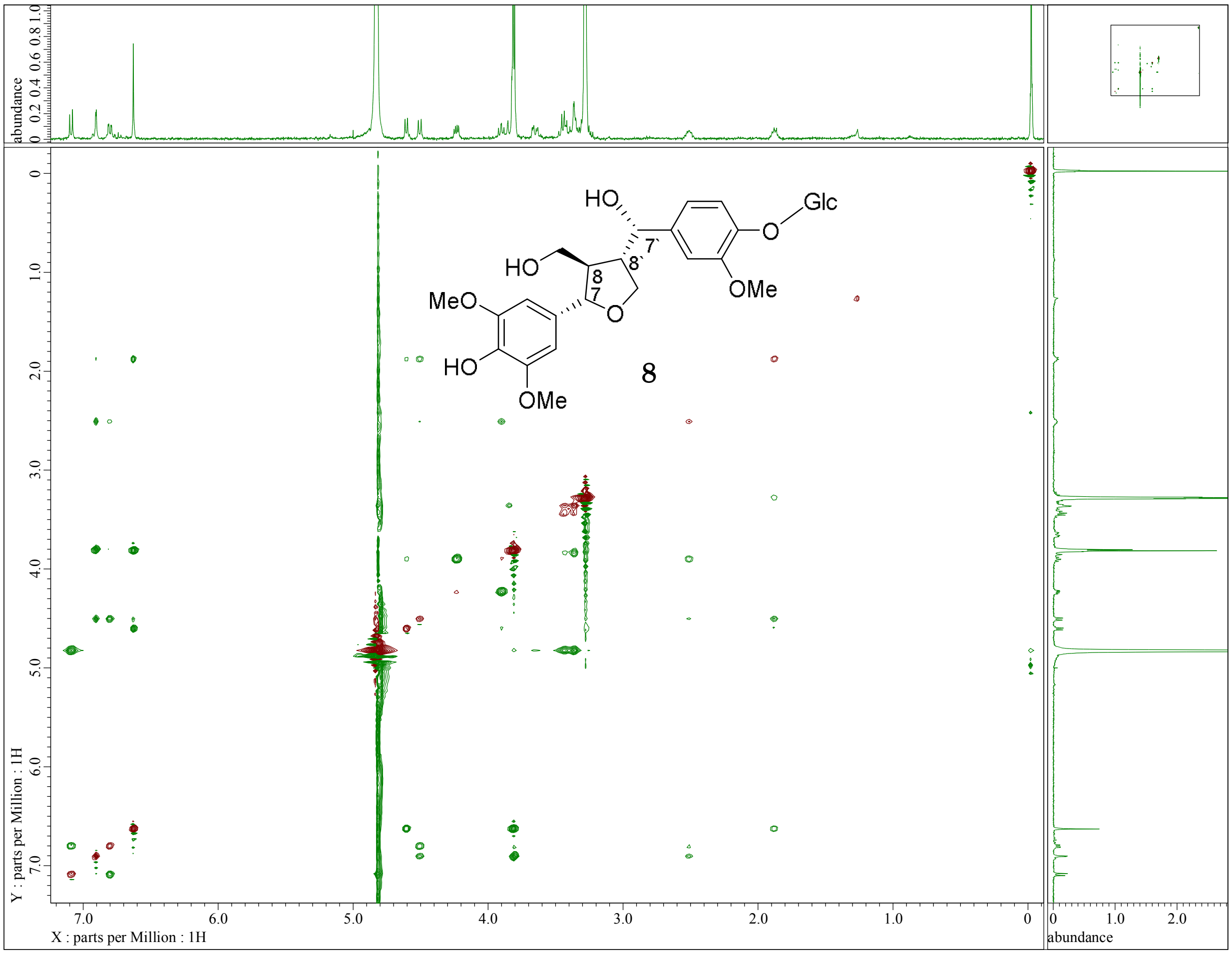

You created this PDF from an application that is not licensed to print to novaPDF printer (http://www.novapdf.com)

Figure S51. ^{1}H MNR spectrum of **9** (400MHz, in CD_3OD)

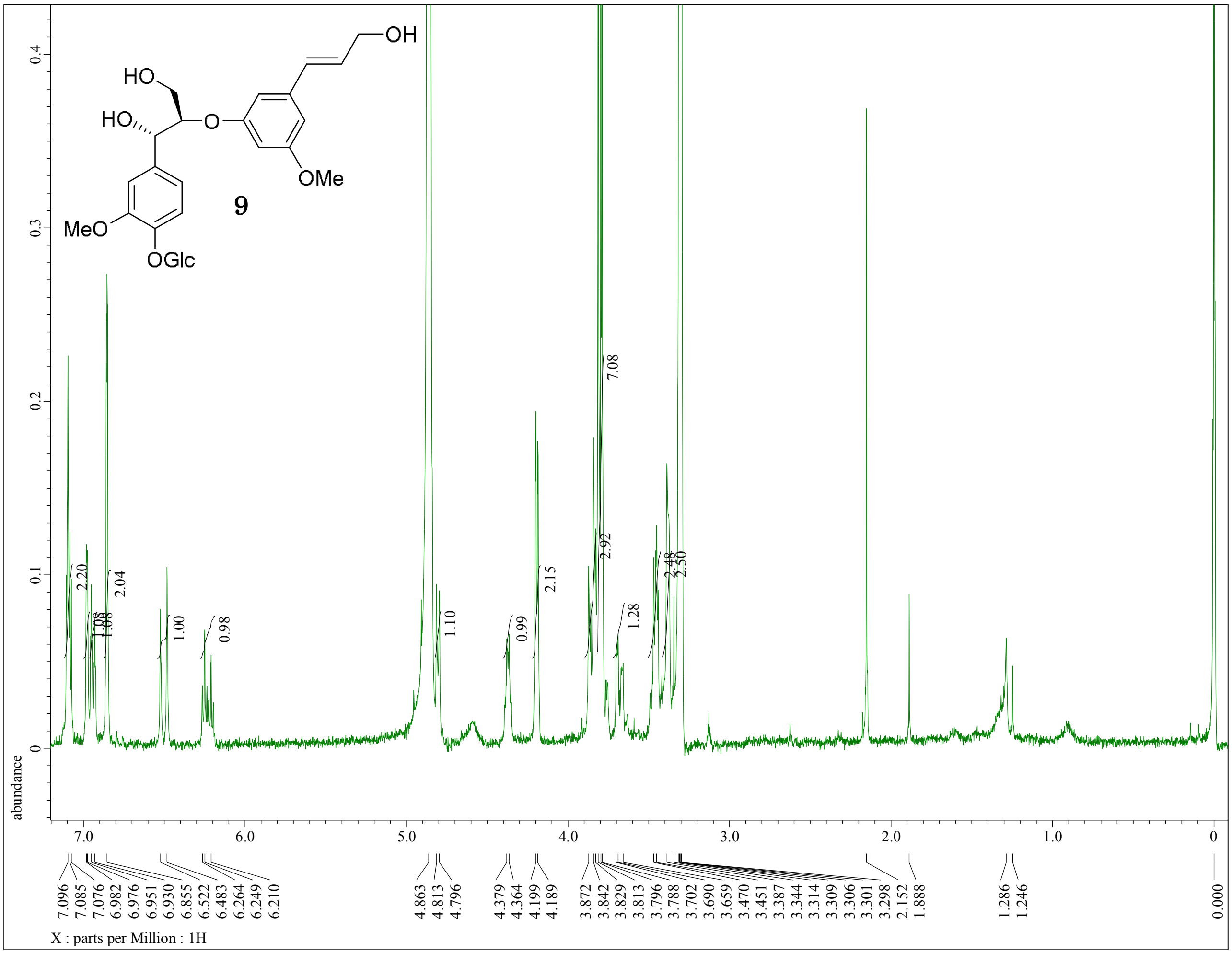

You created this PDF from an application that is not licensed to print to novaPDF printer (http://www.novapdf.com)

Figure S52. ^{13}C MNR spectrum of **9** (100MHz, in CD_3OD)

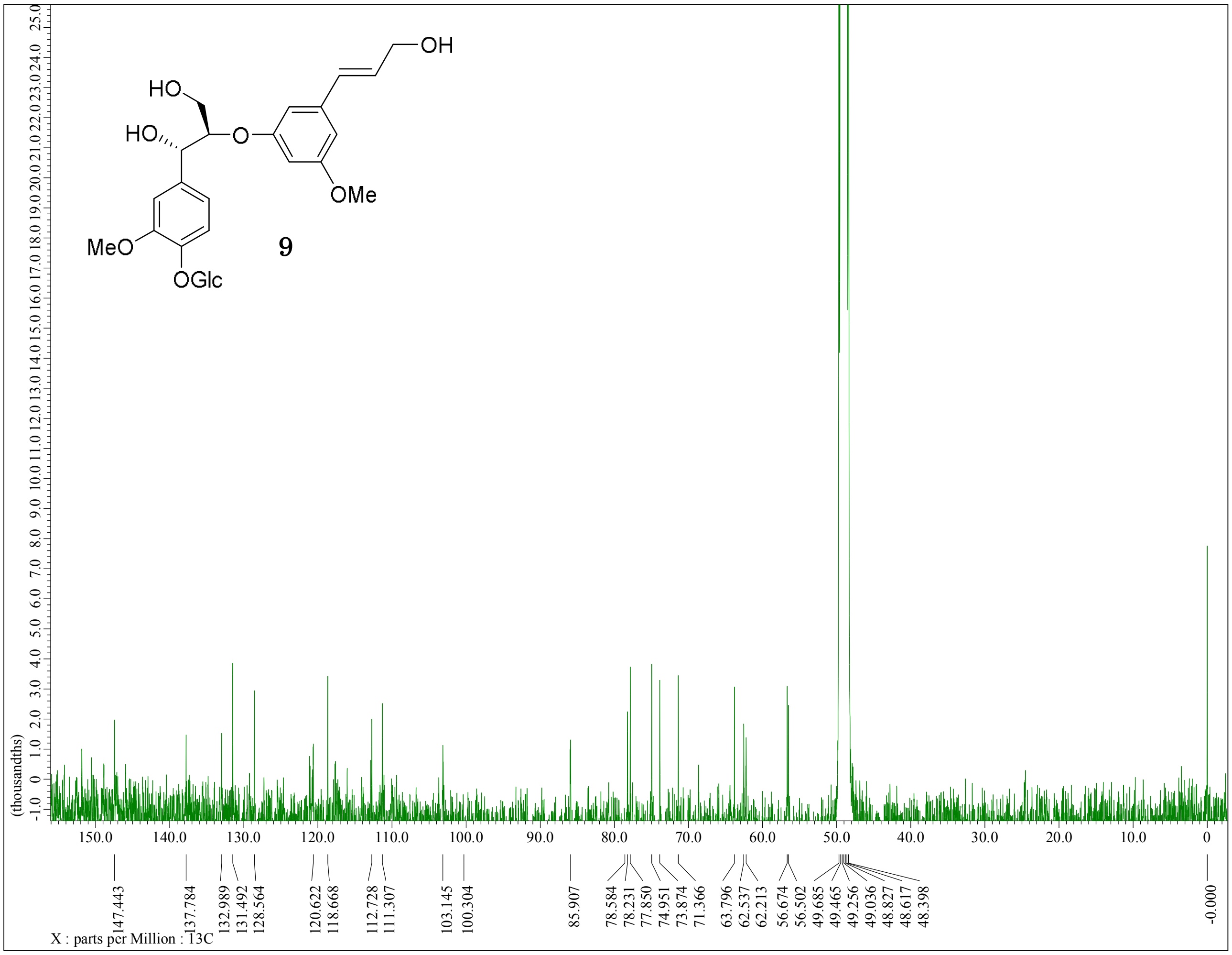

You created this PDF from an application that is not licensed to print to novaPDF printer (http://www.novapdf.com)

Figure S53. H-H COSY experiment of **9** (400MHz, in CD_3OD)

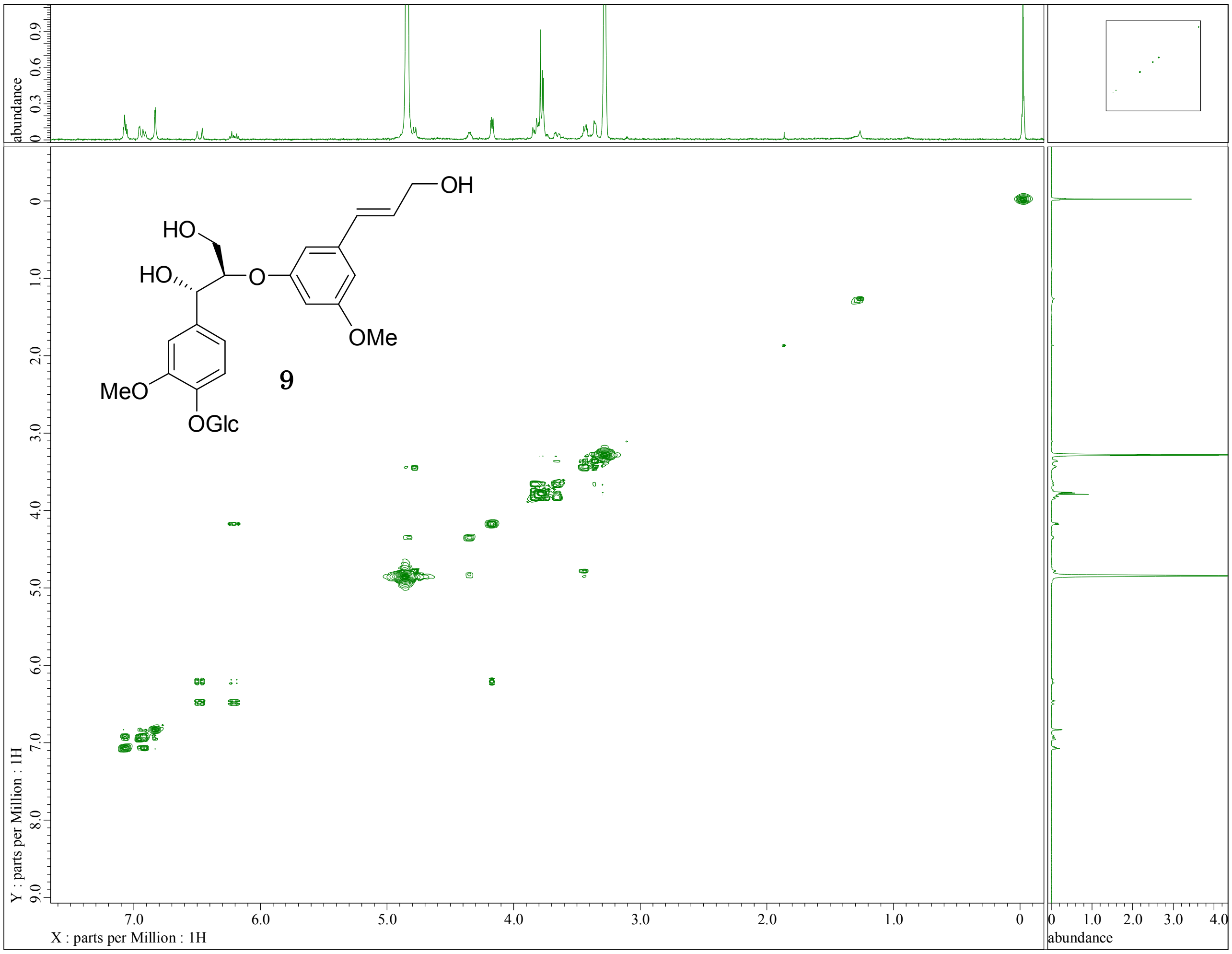

You created this PDF from an application that is not licensed to print to novaPDF printer (http://www.novapdf.com)

Figure S54. HMQC experiment of **9** (400MHz, in CD_3OD).

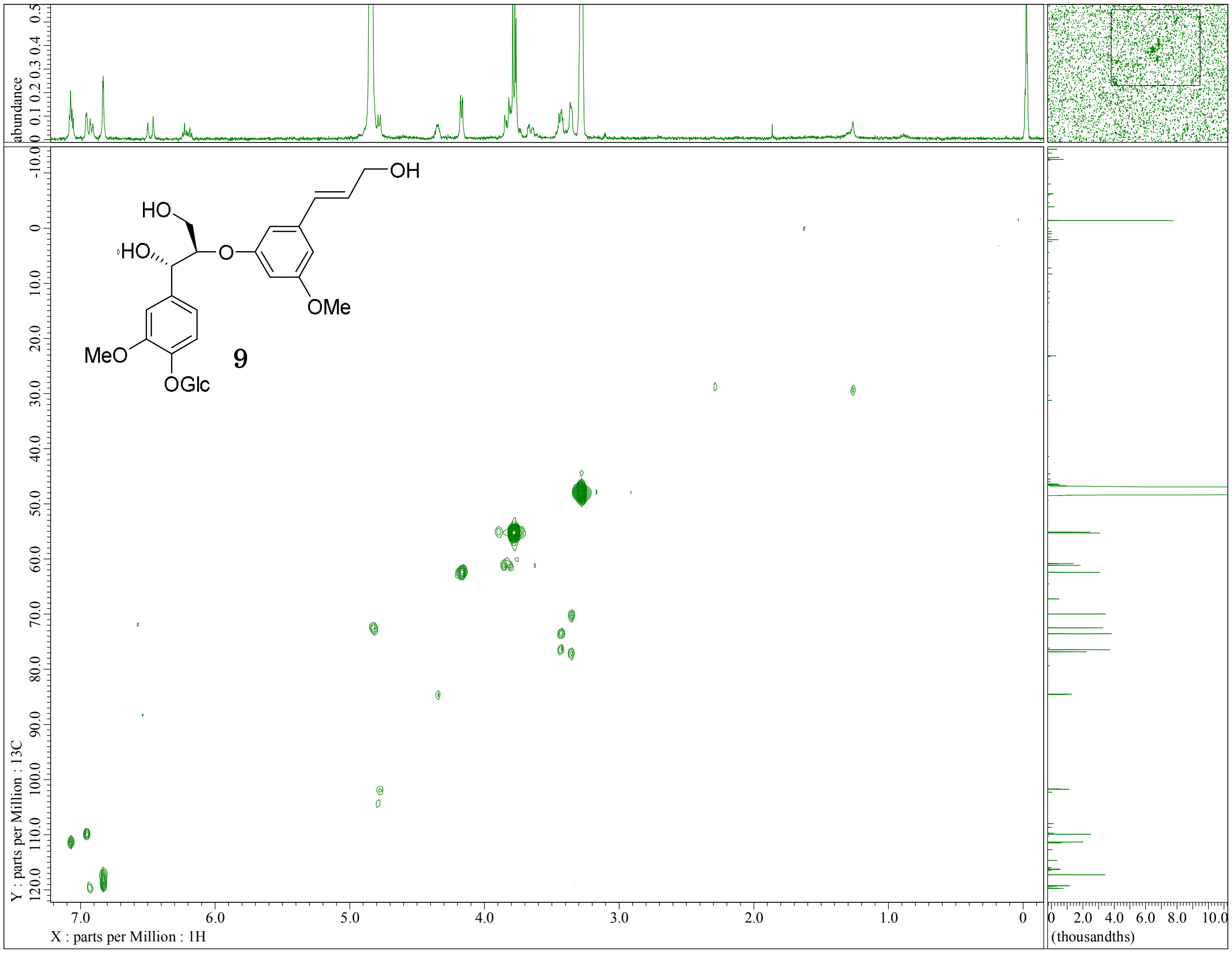

You created this PDF from an application that is not licensed to print to novaPDF printer (http://www.novapdf.com)

Figure S55. HMBC experiment of **9** (400MHz, in CD_3OD).

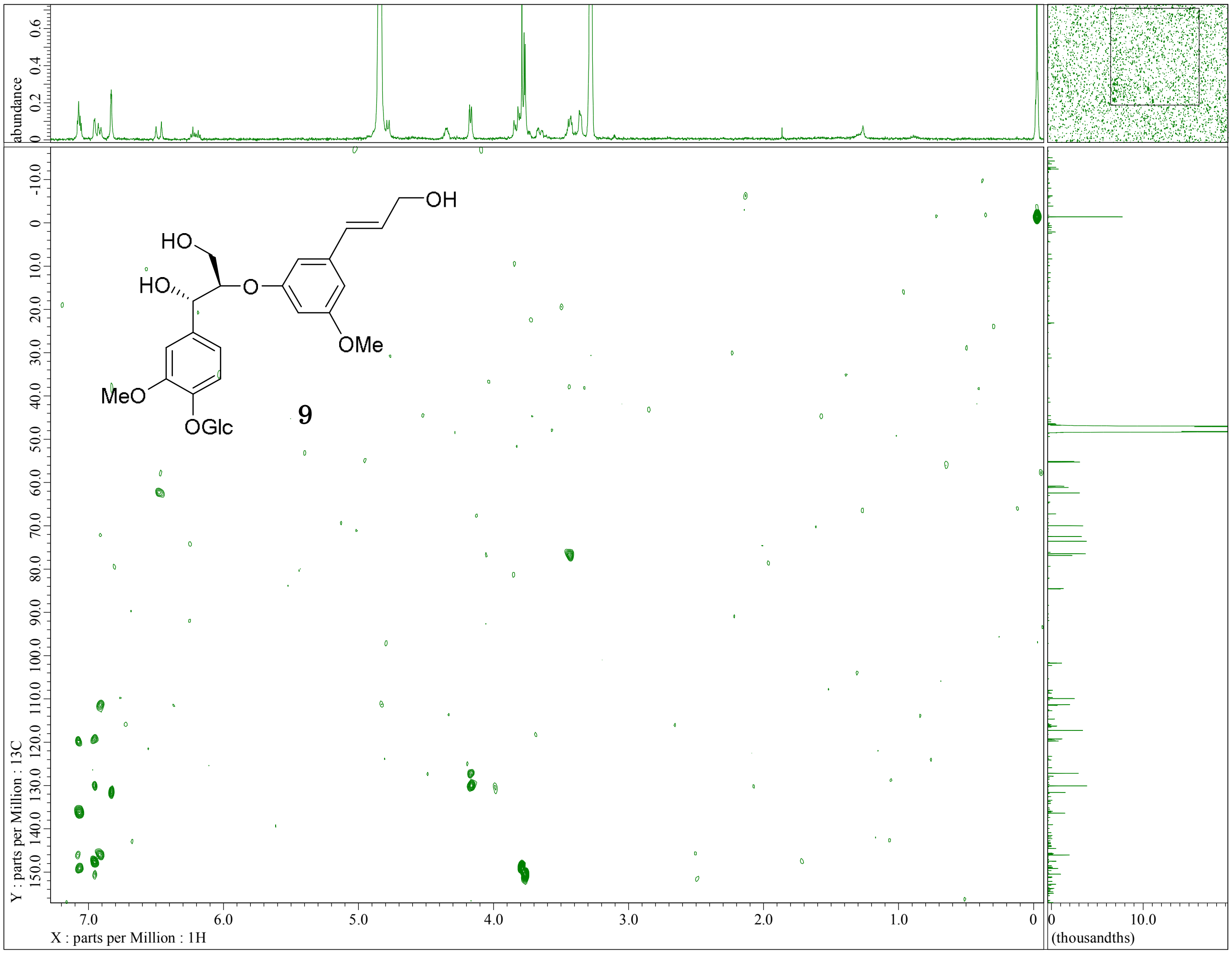

You created this PDF from an application that is not licensed to print to novaPDF printer (http://www.novapdf.com)

Figure S56. NOESY experiment of **9** (400MHz, in CD_3OD).

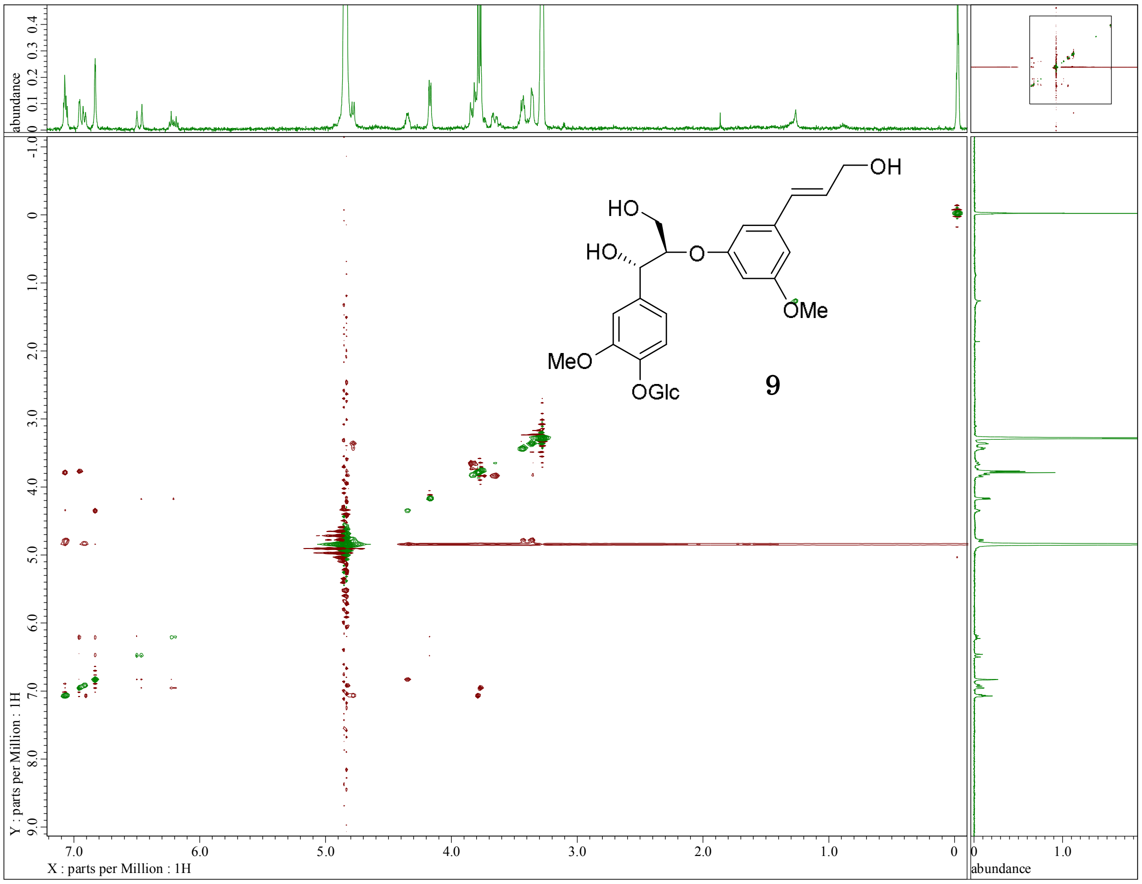

You created this PDF from an application that is not licensed to print to novaPDF printer (http://www.novapdf.com)

Figure S57. ^{1}H NMR spectrum of **9** (400MHz, in DMSO-d_6).

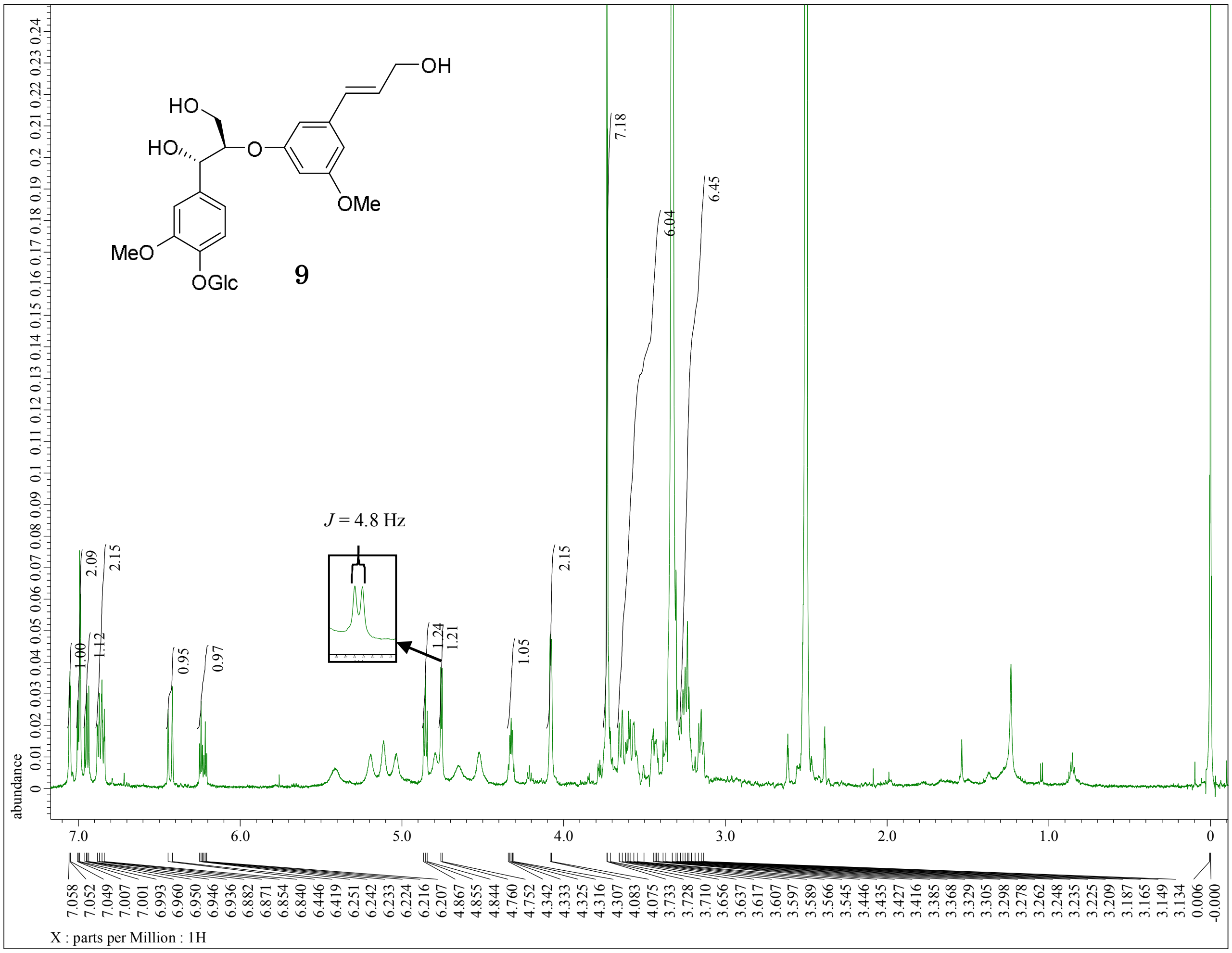

You created this PDF from an application that is not licensed to print to novaPDF printer (http://www.novapdf.com)

Figure S58. HRFABMS data of **1**.

Data : EI-HR-2017-066 Date : 09-Mar-2018 15:59
Instrument : MStation
Sample : 16SA-HPH-F13-e-1
Note : No.225
Inlet : Direct Ion Mode : EI+
RT : 2.50 min Scan# : 31
Elements : C 25/0, H 43/0, O 3/0
Mass Tolerance : 1000ppm, 5mmu if m/z < 5, 50mmu if m/z > 50
Unsaturation (U.S.) : -0.5 - 10.0

	Observed m/z	Int%	Err[ppm / mmu]	U.S.	Composition
1	390.3137	21.80	+0.8 / +0.3	5.0	C25 H42 O3

Figure S59. HRFABMS data of **2**.

Data : EI-HR-2017-008 Date : 27-Jun-2017 11:33
Instrument : MStation
Sample : 16SA-HPE-F3-b-4
Note : No.33
Inlet : Direct Ion Mode : EI+
RT : 1.50 min Scan# : 19
Elements : C 29/0, H 50/0, O 3/0
Mass Tolerance : 1000ppm, 5mmu if m/z < 5, 50mmu if m/z > 50
Unsaturation (U.S.) : -0.5 - 10.0

	Observed m/z	Int%	Err[ppm / mmu]	U.S.	Composition
1	446.3743	27.14	-3.8 / -1.7	5.0	C29 H50 O3

You created this PDF from an application that is not licensed to print to novaPDF printer (http://www.novapdf.com)

Figure S60. HRFABMS data of **3**.

Data : EI-HR-2017-065   Date : 09-Mar-2018 15:53
Instrument : MStation
Sample : 16SA-HPE-F3-b-2-a
Note : No.226
Inlet : Direct   Ion Mode : EI+
RT : 2.25 min   Scan# : 28
Elements : C 25/0, H 44/0, O 3/0
Mass Tolerance : 1000ppm, 5mmu if m/z < 5, 50mmu if m/z > 50
Unsaturation (U.S.) : -0.5 - 10.0

	Observed m/z	Int%	Err[ppm / mmu]	U.S.	Composition
1	392.3302	26.64	+2.9 / +1.2	4.0	C25 H44 O3

Figure S61. HRFABMS data of **4**.

Data : 分子生薬学_20180117108   Date : 03-Sep-2018 11:22
Instrument : MStation
Sample : 16SA_HPB_F1_e
Note : Nacl
Inlet : Direct   Ion Mode : FAB+
RT : 6.61 min   Scan# : 70
Elements : C 38/0, H 46/0, O 3/0, Na 1/0
Mass Tolerance : 1000ppm, 5mmu if m/z < 5, 50mmu if m/z > 50
Unsaturation (U.S.) : -0.5 - 5.0

	Observed m/z	Int%	Err[ppm / mmu]	U.S.	Composition
1	441.3357	100.00	+2.[illegible] / +1.2	4.5	C27 H46 O3 Na

You created this PDF from an application that is not licensed to print to novaPDF printer (http://www.novapdf.com)

Figure S62. HRFABMS data of **5**.

Data : EI-HR-2017-027 Date : 18-Oct-2017 13:51
Instrument : MStation
Sample : 16SA-HPE-F4-d-1
Note : No.91
Inlet : Direct Ion Mode : EI+
RT : 14.33 min Scan# : 173
Elements : C 29/0, H 50/0, O 3/0
Mass Tolerance : 1000ppm, 5mmu if m/z < 5, 500mmu if m/z > 500
Unsaturation (U.S.) : -0.5 - 20.0

	Observed m/z	Int%	Err[ppm / mmu]	U.S.	Composition
1	446.3749	25.71	-2.5 / -1.1	5.0	C29 H50 O3

Figure S63. HRFABMS data of **6**.

Data : EI-HR-2017-025 Date : 18-Oct-2017 10:28
Instrument : MStation
Sample : 16SA-HP-LHe-F6-5-e-1
Note : No.93
Inlet : Direct Ion Mode : EI+
RT : 10.58 min Scan# : 128
Elements : C 25/0, H 44/0, O 4/0
Mass Tolerance : 1000ppm, 5mmu if m/z < 5, 500mmu if m/z > 500
Unsaturation (U.S.) : -0.5 - 10.0

	Observed m/z	Int%	Err[ppm / mmu]	U.S.	Composition
1	408.3230	26.36	-2.4 / -1.0	4.0	C25 H44 O4

You created this PDF from an application that is not licensed to print to novaPDF printer (http://www.novapdf.com)

Figure S64. HRFABMS data of **7**.

Data : EI-HR-2017-064 Date : 09-Mar-2018 15:46
Instrument : MStation
Sample : 16SA-HPE-F4-f-2-b
Note : No.227
Inlet : Direct Ion Mode : EI+
RT : 3.84 min Scan# : 47
Elements : C 28/0, H 50/0, O 4/0
Mass Tolerance : 1000ppm, 5mmu if m/z < 5, 50mmu if m/z > 50
Unsaturation (U.S.) : -0.5 - 10.0

	Observed m/z	Int%	Err[ppm / mmu]	U.S.	Composition
1	450.3702	16.95	-1.6 / -0.7	4.0	C28 H50 O4

Figure S65. HRFABMS data of **8**.

Data : 分子生薬学_20180117330 Date : 23-Oct-2019 16:50
Instrument : MStation
Sample : 16SA_HPB_F5_a_4_b
Note : NBA+NaCl
Inlet : Direct Ion Mode : FAB+
RT : 2.22 min Scan# : 20
Elements : C 27/0, H 36/0, O 13/0, Na 1/0
Mass Tolerance : 1000ppm, 5mmu if m/z < 5, 50mmu if m/z > 50
Unsaturation (U.S.) : -0.5 - 10.0

	Observed m/z	Int%	Err[ppm / mmu]	U.S.	Composition
1	591.2022	34.41	-5.3 / -3.2	9.5	C27 H36 O13 Na

You created this PDF from an application that is not licensed to print to novaPDF printer (http://www.novapdf.com)

Figure S66. HRFABMS data of **9**.

Data : 分子生薬学_20180117331   Date : 23-Oct-2019 17:12
Instrument : MStation
Sample : 16SA_HPB_F5_d_1_c_1_b
Note : NBA+NaCl
Inlet : Direct   Ion Mode : FAB+
RT : 2.69 min   Scan# : 24
Elements : C 26/0, H 34/0, O 12/0, Na 1/0
Mass Tolerance   : 1000ppm, 5mmu if m/z < 5, 50mmu if m/z > 50
Unsaturation (U.S.) : -0.5 - 10.0

	Observed m/z	Int%	Err[ppm / mmu]	U.S.	Composition
1	561.1958	7.66	+1.8 / +1.0	9.5	C26 H34 O12 Na

You created this PDF from an application that is not licensed to print to novaPDF printer (http://www.novapdf.com)
